# Obesity-associated meta-inflammation alters influenza A antiviral responses in Göttingen minipigs

**DOI:** 10.1016/j.isci.2026.117056

**Published:** 2026-08-24

**Authors:** Betina Lyngfeldt Henriksen, Sofie Maiken Riisgård Starbæk, Louise Brogaard, Charlotte Kristensen, Siri Wolthoorn, Louise Lohse, Anders Stockmarr, Henrik Elvang Jensen, Lars E. Larsen, Peter Mikael Helweg Heegaard, Kerstin Skovgaard

**Affiliations:** 1 Department of Biotechnology and Biomedicine, DTU Bioengineering, Technical University of Denmark, Kongens Lyngby, Denmark; 2 Department of Veterinary and Animal Sciences, University of Copenhagen, Frederiksberg, Denmark; 3 Virologi og Mikrobiologisk Beredskab, Statens Serum Institut, Copenhagen, Denmark; 4 Department of Applied Mathematics and Computer Science, Technical University of Denmark, Kongens Lyngby, Denmark; 5 Department of Health Technology, Technical University of Denmark, Kongens Lyngby, Denmark

**Keywords:** influenza A virus, diet-induced obesity, meta-inflammation, innate antiviral immune response

## Abstract

Obesity is a well-recognized risk factor for increased severity following influenza A virus (IAV) infection, likely by promoting meta-inflammation and immune dysregulation, but its effects on antiviral and inflammatory responses remain poorly understood. Using a Göttingen minipig model of diet-induced obesity, we compared antiviral and inflammatory responses before and after IAV infection. Respiratory tract transcriptomics and histopathology revealed no clear obesity-associated immune or inflammatory changes in uninfected minipigs. In contrast, obese pigs showed reduced viral clearance in nasal mucosal tissue accompanied by altered antiviral gene expression pattern 4 days post-infection. Furthermore, cellular infiltration of the respiratory tract was observed in obese pigs after infection. Finally, C-reactive protein concentrations increased significantly in obese pigs, indicating enhanced systemic inflammation in response to respiratory infection. These findings demonstrate that diet-induced obesity in Göttingen minipigs is associated with altered antiviral and inflammatory responses following IAV infection and support the translational value of the obese minipig model.

## Highlights

• Diet-induced obesity impaired influenza A virus clearance in minipigs
• Obese pigs showed altered antiviral and interferon-stimulated responses
• Influenza induced increased respiratory and systemic inflammation in obesity
• Obese Göttingen minipigs support translational influenza research

## Graphical abstract

**Figure:**
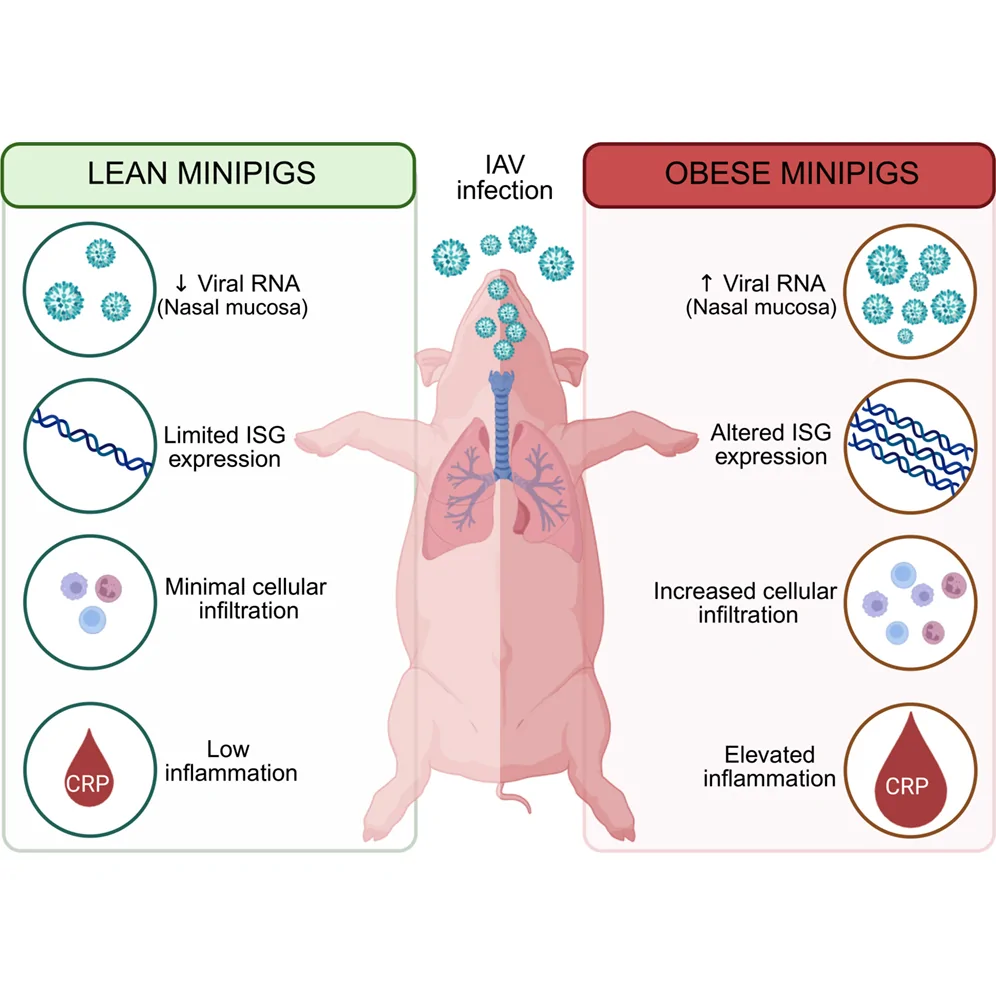

## Teaser

Immune response; Porcine immunology; Virology

## Introduction

Influenza A virus (IAV) poses a constant pandemic threat and represents a significant global health challenge with IAV infections causing 3–5 million cases of severe illness and up to 650,000 deaths annually.^1^^,^^2^ Individuals living with obesity (BMI ≥ 30 kg/m^2^) are more susceptible to respiratory diseases such as influenza and COVID-19, and the risk of severe disease progression and hospitalization is increased.^3^^,^^4^^,^^5^^,^^6^ With obesity reaching epidemic proportions worldwide, this represents an escalating public health challenge. Meta-inflammation, the low-grade inflammatory state associated with obesity, is considered as centrally involved in the adverse outcomes of influenza and COVID-19 infections, though the precise mechanisms remain to be elucidated.^7^^,^^8^ However, understanding these mechanisms is crucial if we are to tailor antiviral drugs and vaccines for protecting this increasing high-risk population.

The immune response to IAV infection involves pattern recognition receptors (PRRs) and downstream effectors like interferon regulatory factor 7 (*IRF7*) that trigger interferon (IFN) production and subsequent induction of antiviral interferon-stimulated gene (ISG) and chemokine expression, the latter causing immune cell infiltration in the affected tissues. These responses are balanced by inflammatory mediators to control viral replication and minimize tissue damage.^9^^,^^10^^,^^11^ Obesity induced meta-inflammation may alter these defense processes, dysregulate antiviral gene expression, and impair immune cell function.^12^^,^^13^^,^^14^ Such disruptions in immune regulation may contribute to a weakened antiviral responses and more severe disease outcomes observed in individuals living with obesity.^4^^,^^10^ This was demonstrated by studies in obese mice and ferrets, which have shown impaired viral clearance and delayed type I IFN responses, with compromised immune cell function and increased disease severity.^13^^,^^14^^,^^15^

Here, we employ the Göttingen minipig as a large animal model with high translational validity for the study of IAV infections, obesity, and meta-inflammation.^16^ Pigs are naturally susceptible to the same IAV subtypes as humans and closely mirror human clinical manifestations, respiratory physiology, and immune responses.^11^ Moreover, they exhibit similar metabolic and inflammatory responses to diet-induced obesity,^16^^,^^17^^,^^18^ making them invaluable models for understanding host-pathogen interactions in the context of obesity.

In this study, we employed the same cohort of Göttingen minipigs from our previously described model of diet-induced obesity and meta-inflammation,^16^ evidenced by long-term increases in the inflammatory acute-phase protein, C-reactive protein (CRP), over a period of more than 11 weeks on a high fat, high fructose, and high cholesterol (HFFC) diet.^16^ Further, we showed that obese pigs developed neutrophilia and increased gene expression of acute-phase proteins and inflammatory cytokines in circulating leukocytes, visceral adipose tissue, and liver.^16^ Building on this cohort of minipigs, the present study examines antiviral immune responses in nasal mucosa, upper and lower trachea, and lung before and after IAV infection. Using our established minipig model of diet-induced obesity associated meta-inflammation,^16^ this study aimed to investigate how obesity modulates the dynamics of antiviral and inflammatory responses to IAV infection, thereby providing a translational bridge between existing knowledge and clinically relevant large-animal models.

## Results

### Obesity was associated with increased viral load in respiratory tissues

**Figure 1:**
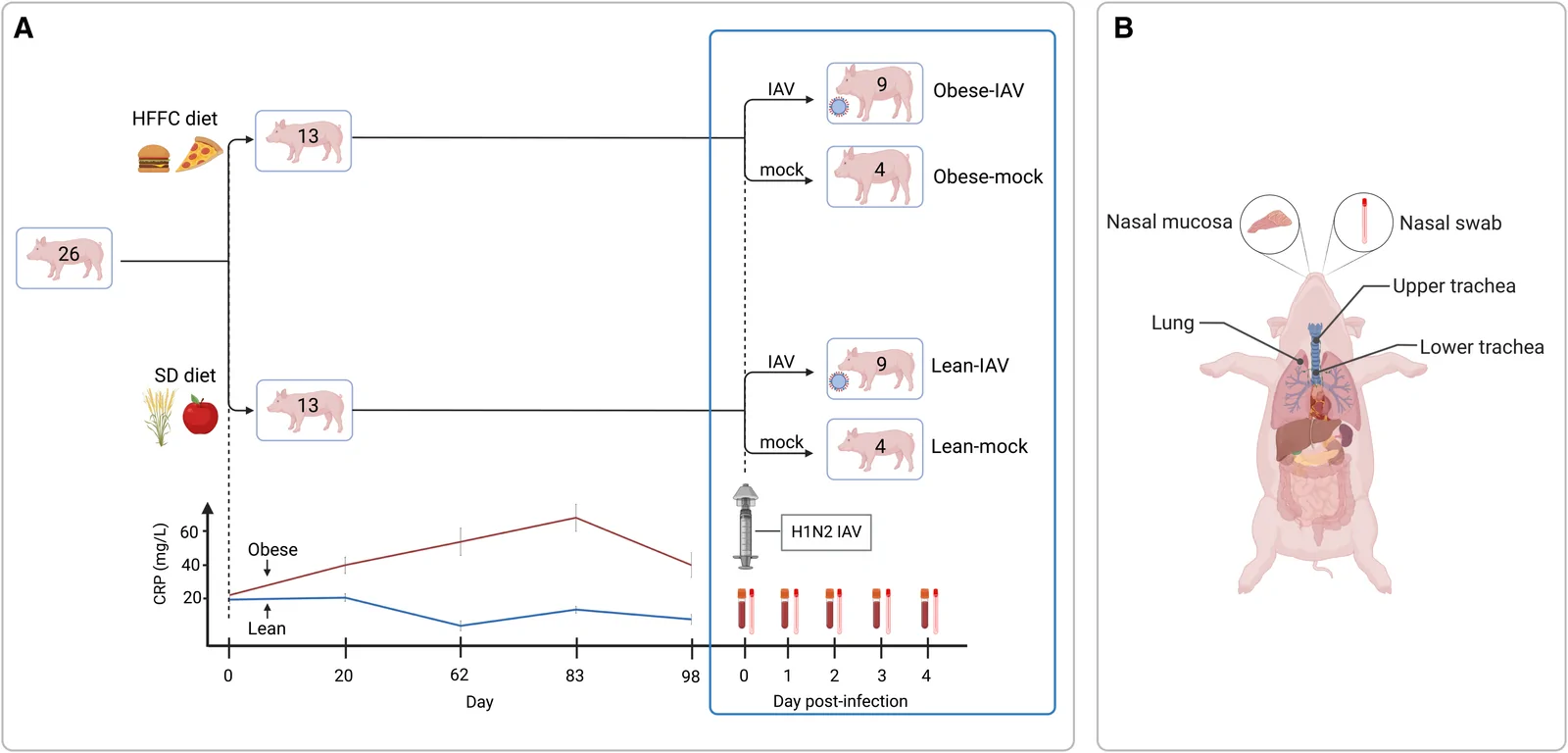
Experimental setup (A) Göttingen minipigs (*n* = 26) were fed either a high fat, high fructose, and high cholesterol (HFFC) diet (obese, *n* = 13) or a standard diet (SD) (lean, *n* = 13) for 103 days. On day 99, pigs from each dietary group were either inoculated with IAV (A/swine/Denmark/12687/2003) (obese-IAV, *n* = 9; lean-IAV, *n* = 9) or mock inoculated with virus-free virus growth medium (obese-mock, *n* = 4; lean-mock, *n* = 4). Serum C-reactive protein data from the diet period (red: obese diet; blue: lean) has been previously published.^16^ The blue square indicates the part of the study presented in this paper. (B) Sample sites used for studying the local antiviral immune response. Created with BioRender.com.

**Figure 2:**
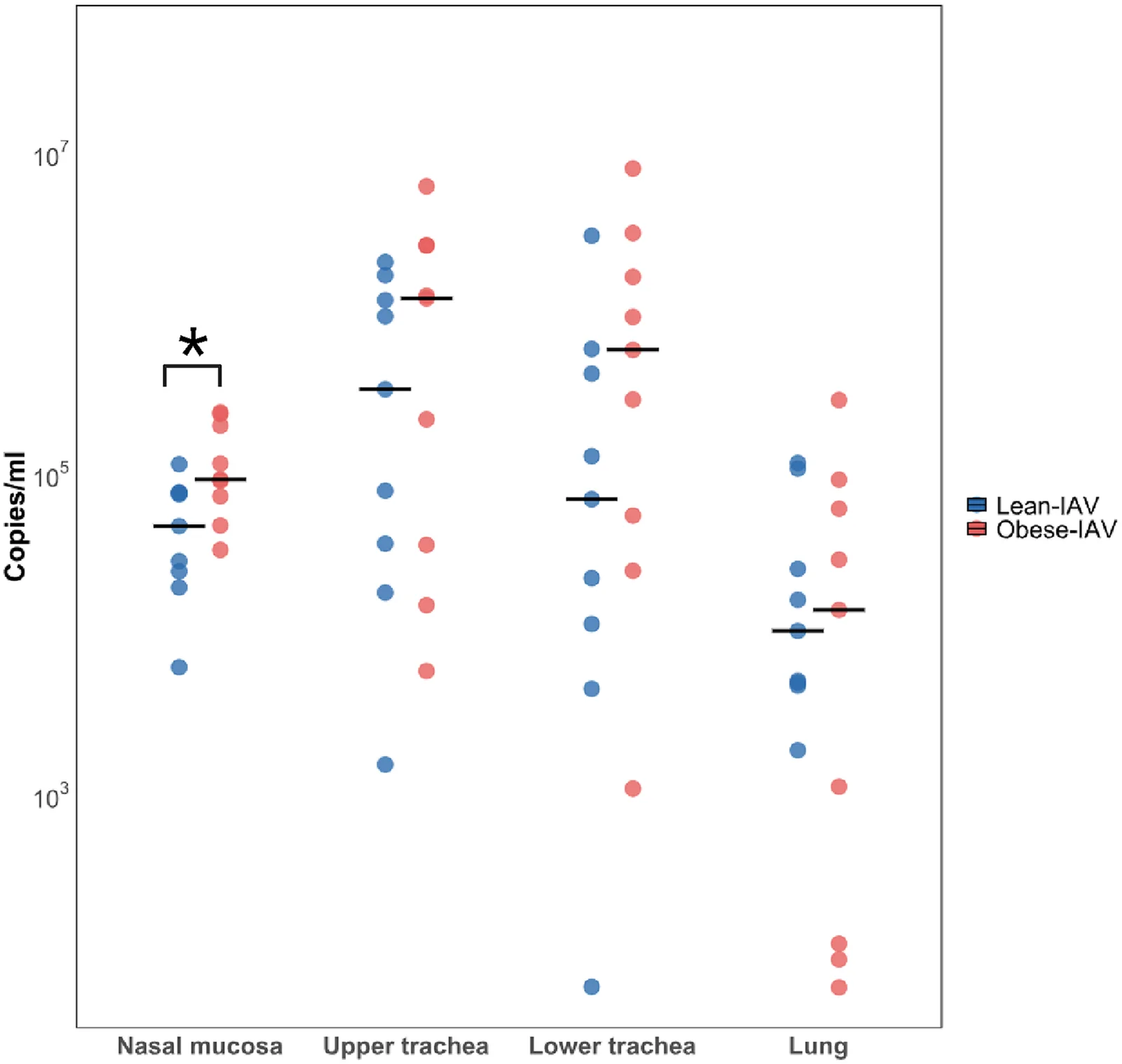
Viral RNA load was increased in the upper airways at 4 days post-inoculation in obese pigs Each pig is represented as a dot colored according to their respective group. The black lines represent median viral load. All mock inoculated pigs were tested negative for influenza. *n* = 4 (lean-mock), *n* = 4 (obese-mock), *n* = 9 (lean-IAV), and *n* = 9 (obese-IAV) individual animals. Statistics were performed using an independent two-sample *t* test. ∗*p* < 0.05.

An overview of the experimental design, including the infection groups, sampling time point, and tissues analyzed, is provided in Figure 1. Viral RNA load was assessed at four different sites in the respiratory tract: nasal mucosa, upper trachea, lower trachea, and lung. Significantly higher levels of viral RNA were found in the nasal mucosal tissue in obese pigs (obese-IAV) compared to the lean (lean-IAV) group at 4 days post-inoculation (dpi) (*p* = 0.02, independent two-sample *t* test) (Figure 2). Median viral RNA levels were 9.24 × 10^4^ copies/mL in obese-IAV pigs and 4.73 × 10^4^ copies/mL in lean-IAV pigs in nasal mucosal tissue. In the upper trachea, median viral RNA levels were 1.24 × 10^6^ copies/mL in the obese-IAV group and 3.36 × 10^5^ copies/mL in the lean-IAV group. In the lower trachea, median viral RNA levels were 5.92 × 10^5^ and 6.96 × 10^4^ copies/mL, respectively. Although the median viral RNA levels were higher in the obese-IAV group than in the lean-IAV group in both the upper and lower tracheal tissues these differences were not statistically significant (Figure 2). The obese-IAV and lean-IAV groups exhibited similar median values in the lung with no significant differences.

### Virus-induced inflammation was enhanced in obese minipigs

**Figure 3:**
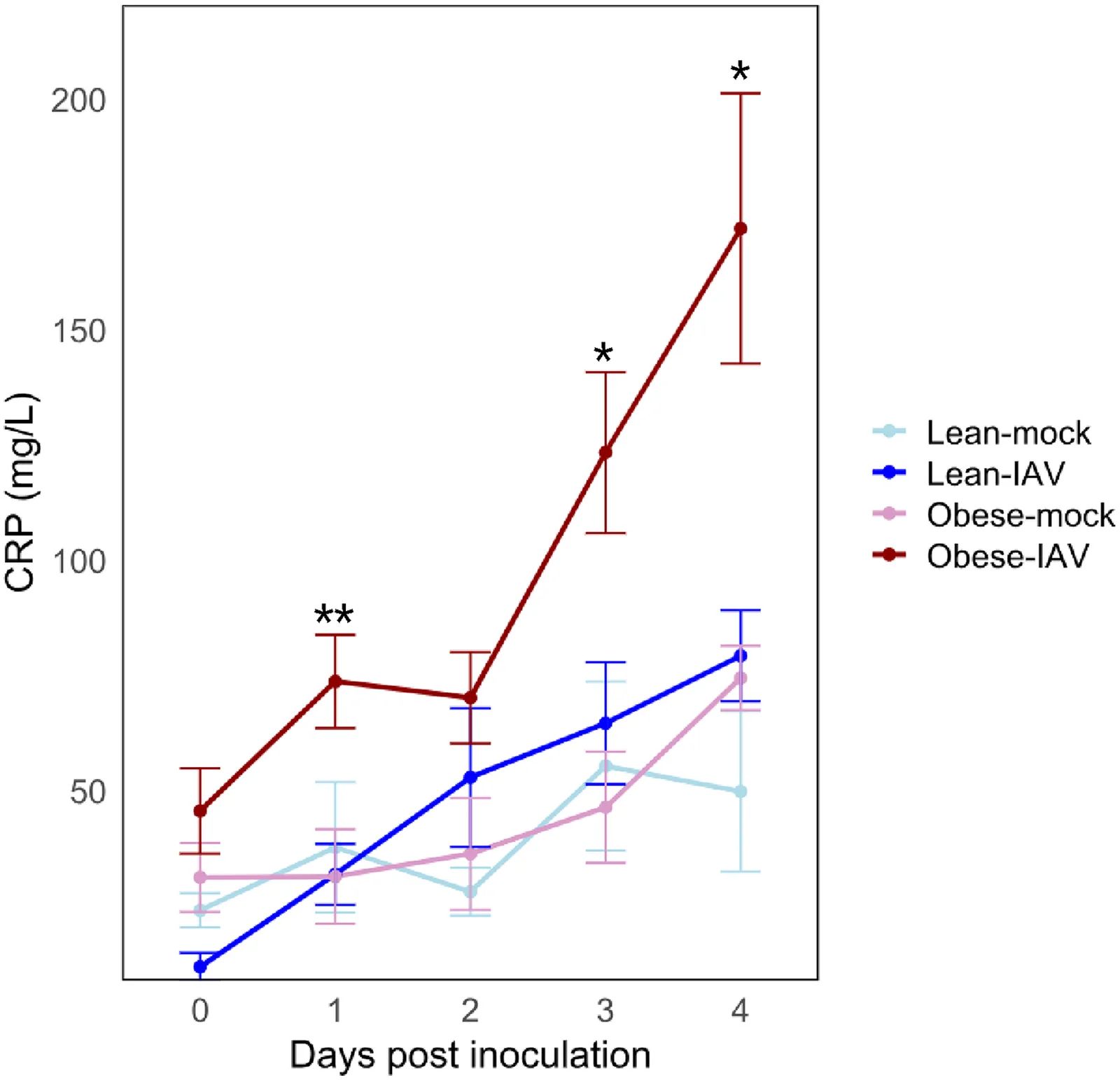
The serum C-reactive protein response to IAV infection was amplified in obese pigs CRP in the obese-IAV group, lean-IAV group, obese-mock group, and lean-mock group. Mean value ±SEM. One animal of the lean-IAV group was excluded at two time points (2 and 3 dpi) due to missing samples. *n* = 4 (lean-mock), *n* = 4 (obese-mock), *n* = 9 (lean-IAV), and *n* = 9 (obese-IAV) individual animals. Statistics were performed using a mixed ANOVA followed by two-sided Welch’s *t* tests, with *p* values adjusted using the Benjamini-Hochberg procedure. ∗*p* ≤ 0.05 and ∗∗*p* ≤ 0.01.

The HFFC 14-week diet regimen induced obesity and meta-inflammation prior to infection in contrast to the control group fed a standard diet. This was evidenced by a significant increase in CRP observed from week 3 onwards, reaching levels approximately 3–5 times higher than typical baseline reported for healthy male Göttingen minipigs, followed by a significant increase in body weight and abdominal circumference from week 7 onwards.^16^^,^^19^ At 0 dpi, the obese-IAV group had a serum CRP concentration of ∼45 mg/L compared with ∼10 mg/L in the lean-IAV group. Four dpi, the average serum CRP concentration in the obese-IAV group was significantly increased by proximately 100 mg/L relative to the obese-mock controls (*p* = 0.01, mixed ANOVA with two-sided Welch’s *t* tests). In contrast, the lean-IAV group showed an increase of ∼25 mg/L by 4 dpi and did not differ significantly from its own uninfected control group (lean-mock). Although the relative increase from day 0 was greater in the lean animals, CRP levels in the lean-IAV group did not differ significantly from their uninfected controls 4 dpi. In contrast, the obese-IAV group showed significantly increased CRP at all time points after infection except for day 2. Together, these findings indicate an increased systemic inflammatory response to IAV infection in the obese pigs (Figure 3).

### Transcriptomic analysis reveals enhanced innate immune activation in the lower trachea of obese minipigs following IAV infection

As the largest difference in viral RNA load between obese-IAV and lean-IAV was observed in the lower trachea at 4 dpi (Figure 2), this site of the respiratory tract was chosen for comprehensive gene expression profiling using RNA sequencing (RNA-seq).

**Figure 4:**
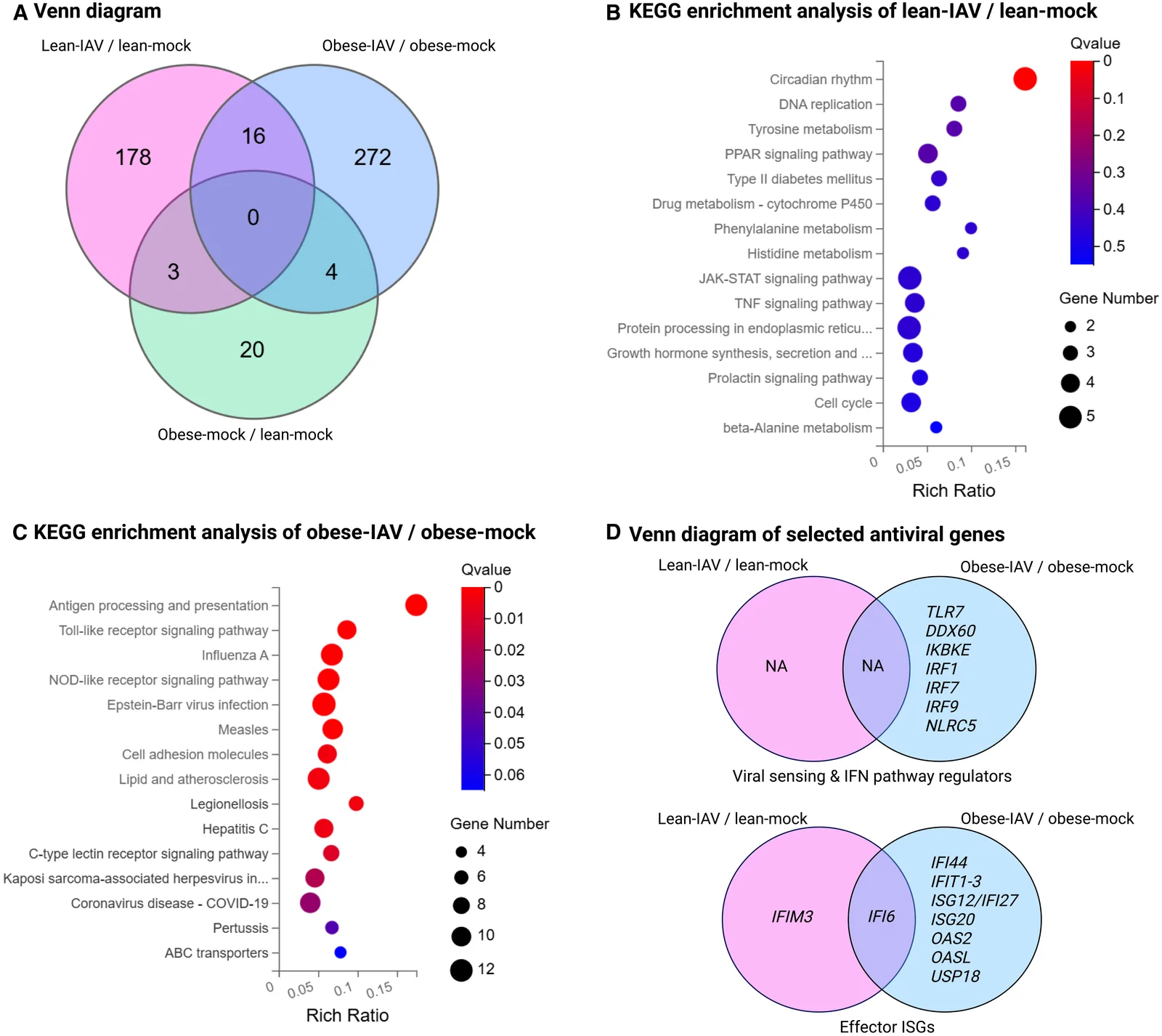
Only pigs with obesity showed enrichment of KEGG pathways associated with innate antiviral immunity in lower tracheal tissue 4 days after influenza A virus infection (A) Venn diagram illustrates the distribution of the significantly differently expressed genes in lower trachea after IAV infection. (B and C) (B) KEGG enrichment analysis identified biological pathways associated with the differentially expressed genes (DEGs) comparing lean-mock with lean-IAV and (C) obese-mock with obese-IAV. The bubble diagram indicates the ratio of enriched DEGs to the total number of identified genes in a certain pathway. Circles indicate the number of genes in the corresponding pathway, and color depicts the *q* value. (D) Venn diagrams of selected significantly expressed antiviral genes for viral sensing and IFN pathway regulators and effector ISGs. *n* = 4 (lean-mock), *n* = 4 (obese-mock), *n* = 9 (lean-IAV), and *n* = 9 (obese-IAV) individual animals. RNA-seq data were normalized and DEGs were identified using DESeq2 and corrected for multiple testing using Benjamini-Hochberg, with DEGs defined by *q* values <0.05 and fold changes of ±1.5.

A total of 493 differentially expressed genes (DEGs) (*q* ≤ 0.05, DESeq2 with Benjamini-Hochberg procedure) were identified in the lower trachea. Of these, 27 DEGs (*q* ≤ 0.05, DESeq2 with Benjamini-Hochberg procedure) were found to be differential expressed when comparing the obese-mock and lean-mock groups and a total of 197 DEGs (*q* ≤ 0.05, DESeq2 with Benjamini-Hochberg procedure) were identified between the lean-IAV and lean-mock groups. (Figure 4A; Table S1). The highest number of DEGs, 292 (*q* ≤ 0.05, DESeq2 with Benjamini-Hochberg procedure) were identified between the obese-mock and obese-IAV groups, of which 272 (*q* ≤ 0.05, DESeq2 with Benjamini-Hochberg procedure) were uniquely associated with this comparison (Figure 4A).

Kyoto encyclopedia of genes and genomes (KEGG) enrichment analysis of the unique DEGs 4 dpi between lean-mock and lean-IAV revealed no significantly enriched pathways associated with immune response to viral infections (Figure 4B), whereas a KEGG enrichment analysis of DEGs between obese-mock and obese-IAV revealed multiple significantly enriched pathways associated with IAV infection, such as “antigen processing and presentation,” “Toll-like receptor signaling pathway,” “Influenza A,” and “NOD-like receptor signaling pathway” (Figure 4C). Several genes involved in viral sensing and interferon regulation including Toll-like receptor 7 (*TLR7*) (*q* = 0.03, DESeq2 with Benjamini-Hochberg procedure), DExD/H-box helicase 60 (*DDX60*) (*q* = 0.001, DESeq2 with Benjamini-Hochberg procedure), and several IRFs (*q* ≤ 0.05, DESeq2 with Benjamini-Hochberg procedure) as well as key ISGs such as interferon-induced protein with tetratricopeptide repeats (IFITs) (*q* ≤ 0.05, DESeq2 with Benjamini-Hochberg procedure), 2′-5′-oligoadenylate synthetase 2 (*OAS2*) (*q* = 0.04, DESeq2 with Benjamini-Hochberg procedure) and OAS-like (*OASL*) (*q* ≤ 0.01, DESeq2 with Benjamini-Hochberg procedure) were significantly upregulated in the obese-IAV group 4 dpi (Figure 4D). The RNA-seq findings were confirmed by microfluidic qPCR, showing consistent gene expression patterns across selected genes (Figure S1). Based on these enriched pathways between obese-mock and obese-IAV, a panel of 24 relevant genes (including reference genes) was selected for quantification by microfluidic qPCR in the nasal mucosa, upper trachea, and lung to study the innate antiviral immune response to infection at multiple respiratory sites (Table S2).

### Obesity induced limited changes in the respiratory tract in the absence of IAV infection

Across all examined parts of the respiratory tract, only the gene encoding heat shock protein 70-2 (*HSP70-2*), involved in cellular stress, inflammation, and metabolic regulation,^20^ was significantly upregulated (*p* = 0.03, Welch’s *t* test) in the lung in obese-mock compared to lean-mock (Table S3). Furthermore, proteomics analysis was performed to quantify protein levels in the lung, and no significant differences in protein abundance were observed between lean and obese pigs prior to infection, except for apolipoprotein E (APOE) (Table S4). Several innate antiviral genes, including *OAS2*, signal transducer and activator of transcription 1 (*STAT1*), and *IRF7* showed trends toward slightly lower expressions in all respiratory tissues examined with microfluidic qPCR in the obese-mock group compared to the lean-mock group, though these differences were not significant (Table S3).

**Figure 5:**
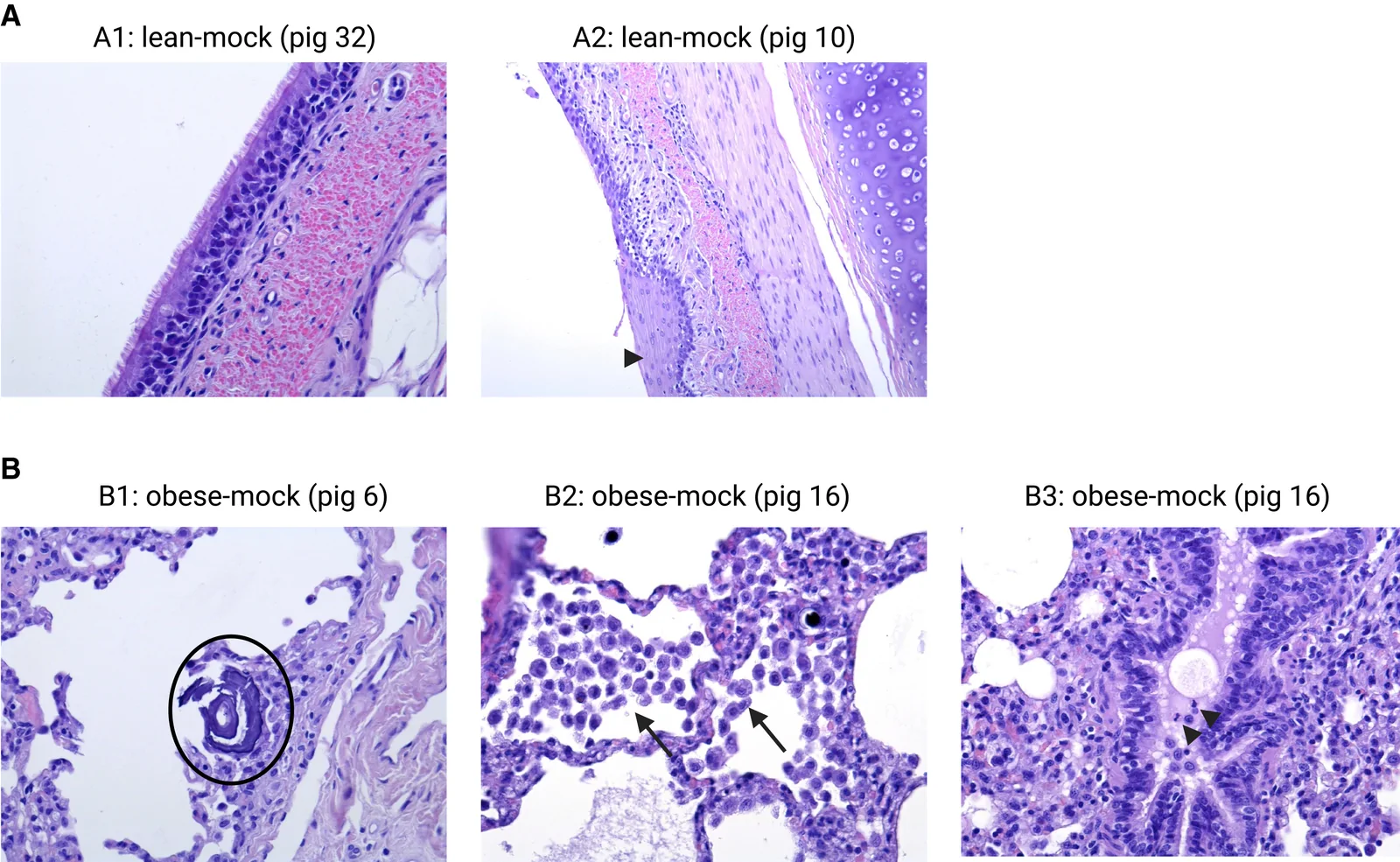
Histopathological changes in upper trachea and lungs in lean and obese mock pigs Representative images of the hematoxylin and eosin (H&E) staining of swine upper tracheal (A) and lung tissue (B). No consistent changes observed in either the lean-mock or obese-mock group of upper trachea (A1), one pig from lean-mock group with metaplastic squamous epithelium (arrowhead) (A2). Pulmonary mineralization in one pig of the obese-mock group (circle) (B1). Increased luminal infiltration of alveolar macrophages (arrows) and a few bronchioles with luminal infiltration of neutrophils (arrowhead) in one pig from the obese-mock group (B2 and B3). *n* = 4 (lean-mock), and *n* = 4 (obese-mock) individual animals.

Histopathological examination of the upper trachea showed no consistent changes in either the lean- or obese-mock groups (Figure 5A1). Focal metaplastic squamous epithelium was observed in one pig from the lean-mock group and one pig in the obese-mock group (Figure 5A2). No changes were observed in the lungs of the lean-mock group (data not shown). In the obese-mock group, one pig showed a focal pulmonary mineralization (Figure 5B1), and another obese-mock pig showed alveolar infiltration of pulmonary macrophages and neutrophils (Figures 5B2 and 5B3). No changes were observed in the lungs of the rest of the pigs from the obese-mock group (data not shown).

### Obesity was associated with changes in interferon-stimulation and immune activation responses to IAV infection in nasal mucosal and tracheal tissues

**Figure 6:**
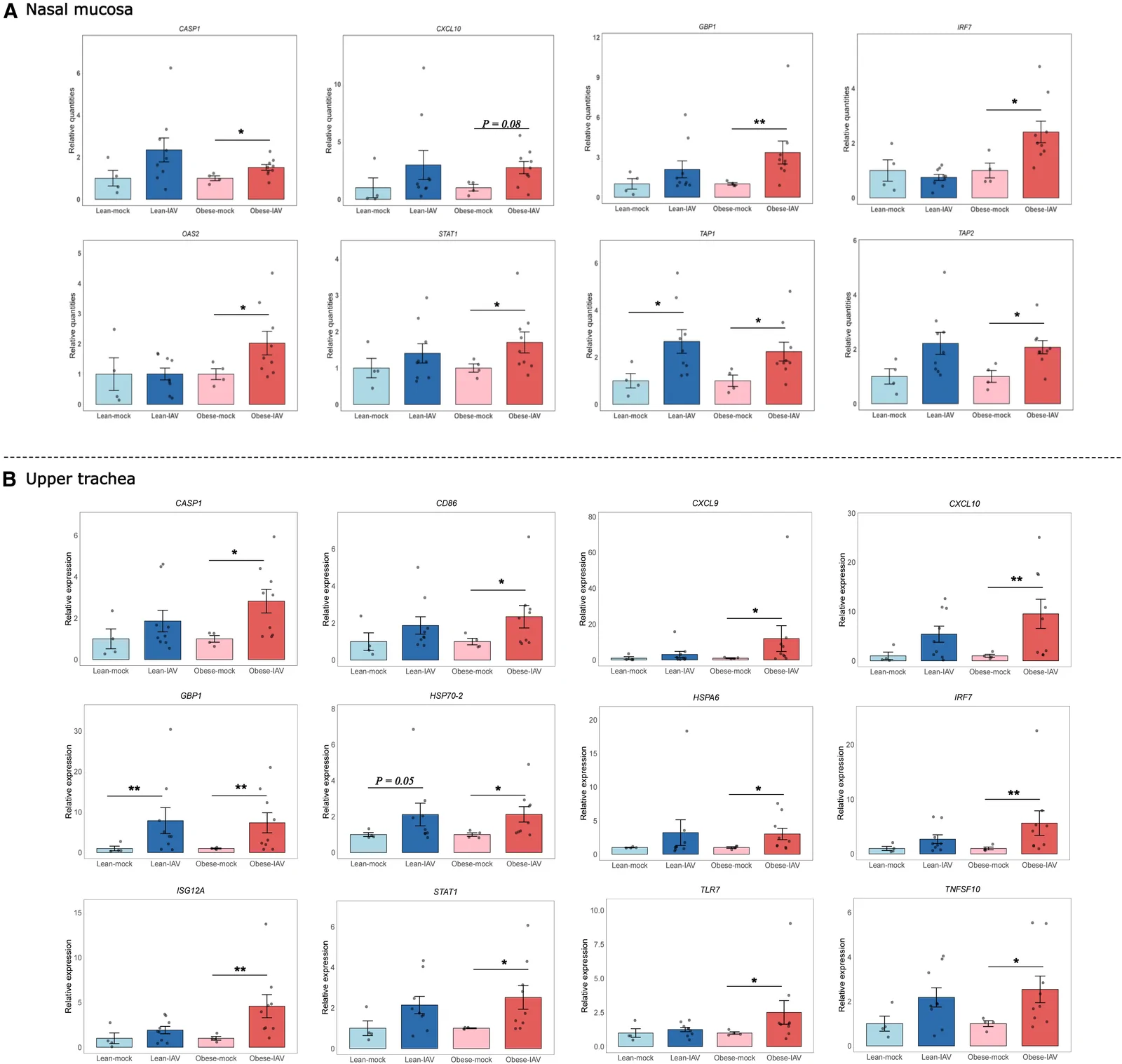
Expression of genes involved in virus recognition, immune responses, and inflammation was increased in nasal mucosa and upper trachea of obese pigs at 4 dpi Gene expression in (A) nasal mucosa, and (B) upper trachea are shown with a fold change of ± 1.5. Mean value ± SEM. Mock groups are set to 1; IAV groups are scaled to their respective mock groups (lean-IAV to lean-mock, obese-IAV to obese-mock). *n* = 4 (lean-mock), *n* = 4 (obese-mock), *n* = 9 (lean-IAV), and *n* = 9 (obese-IAV) individual animals. Statistics were performed using Welch’s *t* test. ∗*p* ≤ 0.05 and ∗∗*p* ≤ 0.01.

Genes involved in interferon-stimulation and anti-viral immune activation in nasopharyngeal swab samples, nasal mucosal tissues, upper trachea, and lung tissues were examined at 4 dpi (Figure 6; Figure S2; Table S5).

Upon infection, a classical innate antiviral immune response was seen in nasopharyngeal swabs samples of both obese and lean animals collected daily before and after infection (Figure S2). Several PRRs and ISGs showed increased expression following infection, with the highest expression detected at 1 and 2 dpi, before returning to baseline levels by day 4 post-infection. No difference was observed between the two diet groups (Figure S2).

In the underlying nasal mucosal tissue, the expression of several ISGs, including guanylate binding protein 1(*GBP1*) (*p* = 0.001, Welch’s *t* test), *OAS2* (*p* = 0.04, Welch’s *t* test), and *STAT1* (*p* = 0.04, Welch’s *t* test) transporter associated with antigen processing 1 (*TAP1*) (*p* = 0.05, Welch’s *t* test), and *TAP2* (*p* = 0.04, Welch’s *t* test) was significantly higher in the obese-IAV group compared to obese-mock on day 4 (Figure 6A). Additionally, expressions of *IRF7* (*p* = 0.03, Welch’s *t* test) and caspase 1 (*CASP1*) (*p* = 0.04, Welch’s *t* test) were significantly elevated in the obese-IAV group compared to obese-mock (Figure 6A). In contrast, in the lean-IAV group, only *TAP1* (*p* = 0.05, Welch’s *t* test) was significantly upregulated relative to lean-mock, although several other genes (*CASP1*, *CXCL10*, *GBP1*, and *TAP2*) showed a trend toward higher expression, albeit not significant (Figure 6A).

In the upper trachea, genes related to the type I interferon response, including *GBP1* (*p* = 0.01, Welch’s *t* test), *IRF7* (*p* = 0.001, Welch’s *t* test), *STAT1* (*p* = 0.01, Welch’s *t* test), *ISG12A* (*p* = 0.002, Welch’s *t* test), *CXCL9* (*p* = 0.01, Welch’s *t* test), and *CXCL10* (*p* = 0.005, Welch’s *t* test), were significantly upregulated in the obese-IAV group compared to the obese-mock group (Figure 6B). In addition, genes involved with virus recognition and innate immunity, such as *TLR7* (*p* = 0.05, Welch’s *t* test), *CASP1* (*p* = 0.008, Welch’s *t* test), and cluster of differentiation 68 (*CD68*) (*p* = 0.04, Welch’s *t* test), also showed significant upregulation. Genes associated with apoptosis and stress responses, including tumor necrosis factor-related apoptosis-inducing ligand 10 (*TNFSF10*) (*p* = 0.02, Welch’s *t* test), heat shock protein family A (Hsp70) member 2 (*HSP70-2*) (*p* = 0.01, Welch’s *t* test), and *HSPA6* (*p* = 0.02, Welch’s *t* test), were likewise significantly elevated in obese-IAV compared to obese-mock. In agreement with the nasal mucosal tissue only a few antiviral innate immune genes were found to be regulated in tracheal tissues of the lean-IAV group 4 days after IAV infection. *GBP1* (*p* = 0.02, Welch’s *t* test) was significantly upregulated in the lean-IAV group, while *HSP70-2* showed borderline upregulation. Although several other genes (e.g., *CD86*, *CXCL10*, *HSPA6*, *STAT1*, and *TNFSF10*) were expressed at slightly higher levels in the lean-IAV group compared to the lean-mock group, these differences were not significant (Figure 6B).

In the lung tissue, no genes were significantly regulated in either the obese-IAV or lean-IAV group compared to their respective control group 4 dpi (Table S5).

### Increased macrophage infiltration was seen in trachea and lung following IAV infection in obese minipigs

**Figure 7:**
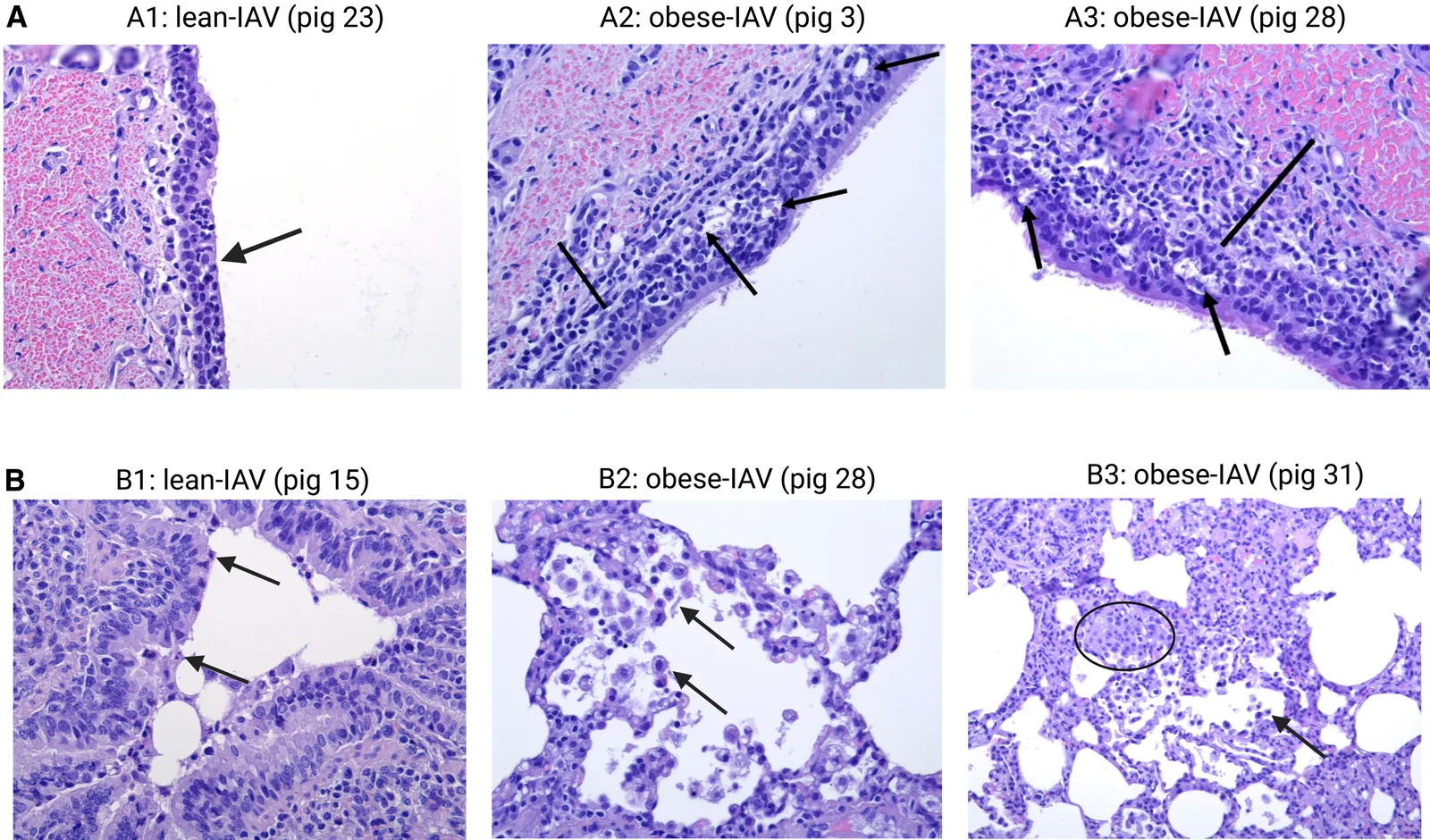
Histopathological changes in upper tracheal and lung tissue from lean and obese pigs after IAV infection Representative images of the hematoxylin and eosin (H&E) staining of upper tracheal (A) and lung (B). (A1) Loss of cilia in trachea in lean-IAV group (arrow). (A2 and A3) Tracheal epithelial vacuolization (arrows) along with infiltration of mononuclear cells in the submucosal layer (lines). (B1) Exudation of a few neutrophils in the bronchiole (arrows). (B2 and B3) Increased number of alveolar macrophages (circle and arrows) in the lungs. *n* = 9 (lean-IAV), and *n* = 9 (obese-IAV) individual animals.

Hematoxylin and eosin (H&E) staining was applied to assess whether obesity influence immune cell infiltration in upper trachea and lung tissues after IAV infection. Histopathological evaluation of upper trachea revealed loss of cilia in two out of four pigs in the lean-IAV group (Figure 7A1). In the obese-IAV group, two out of four pigs showed the presence of mononuclear cells in submucosa with vacuolization of the upper tracheal epithelium after IAV infection with no cilia loss (Figures 7A2 and 7A3).

Examination of the lung tissue showed exudation of a few neutrophils in bronchi and bronchioles 4 dpi in one pig from the lean-IAV group (Figure 7B1), and no changes were observed in the remaining three pigs of the lean-IAV group (data not shown). In the obese group, three out of four obese pigs showed presence of alveolar macrophages in the left cardiac lobe at 4 dpi (Figures 7B2 and 7B3).

## Discussion

This study examined how diet-induced obesity affects the innate antiviral immune response to IAV infection in the upper and lower respiratory tract in a Göttingen minipig model.^16^ We observed stronger antiviral and inflammatory responses in obese pigs compared to lean pigs, alongside an increased systemic inflammatory response to infection. To our knowledge, this is the first study to characterize how innate antiviral defenses, inflammatory genes, and associated pathways are regulated during IAV infection in the presence of obesity and meta-inflammation, using a highly translational large-animal model.

Despite obesity and systemic inflammation, no inflammation was observed in the respiratory tissues of mock-inoculated obese animals. Antiviral and inflammatory genes as well as protein expression patterns in the uninfected respiratory tissues were largely unchanged in obese-mock group compared to lean-mock pigs, which is in contrast to previous reports in respiratory tissues of obese uninfected mice and ferrets^21^^,^^22^ and *in vitro* infected human respiratory cells.^23^ This discrepancy may be explained by the small sample size (*n* = 4), timing of sampling, species- and model-specific responses of Göttingen minipigs, and the potentially different inflammatory phenotype induced by the high fat diet compared to other diets. However, the transcriptomic and proteomic data were concordant in the mock groups, as both gene expression and protein levels indicated minimal baseline inflammation. The agreement between gene and protein data suggests that obesity alone did not induce detectable inflammation in the respiratory tract prior to infection in a minipig model.

In agreement with the low expression of inflammatory genes and proteins in both the lean-mock and obese-mock groups, histopathological examination of the trachea revealed no detectable changes, and presence of immune cells was observed in only one of the four obese animals. In contrast, increased pulmonary immune cell infiltration has been reported in two mice studies where obesity was associated with increased number of neutrophils and macrophages in the lungs.^24^^,^^25^

All four respiratory tissue sites examined showed higher viral RNA load in the obese group at 4 dpi, although only significant in nasal mucosal tissue. These findings contradict previous studies in mouse lung tissue at 3 dpi^13^ and human nasopharyngeal aspirates^23^ where no significant differences in viral RNA load were observed between obese and lean individuals at 24- and 48-h post-infection. However, our results align with a mouse study showing higher viral titers in obese mice at 3 and 5 dpi.^15^ These divergent findings may highlight model-specific differences in obesity-driven immune alterations, differences in experimental systems, and sampling time points.

Having characterized viral RNA levels, we next examined the duration of innate immune activation after infection. In a previous porcine infection study from our group using the same virus strain (A/swine/Denmark/12687/2003 [H1N2]), we showed that viral RNA and the expression of innate antiviral immune genes (including DExD/H-box helicase 58 [*DDX58*], *TLR3*, MX dynamin-like GTPase 1 [*MX1*], and *ISG15*) peaked at 1 dpi and in some cases had declined or returned to baseline at 3 dpi in cross-bred large white × German Landrace pigs.^26^^,^^27^ This relatively rapid normalization of the response is consistent with the lean-IAV group in the present study, where only a limited number of genes were significantly regulated at 4 dpi. In addition, a similar early peak (between day 1 and 2) in innate antiviral immune genes were observed in the mucus isolated from the nasopharyngeal swab samples in the present study. The overall absence of a significant classic antiviral innate immune response in the respiratory tissue 4 dpi in lean pigs likely reflects a return of the immune system to baseline homeostasis following an earlier antiviral peak, consistent with our earlier findings.^26^^,^^27^

In contrast, IAV infection in obese pigs led to a more pronounced antiviral response still maintained at 4 dpi, with increased expression of innate antiviral genes (e.g., *OAS2*, *IRF7*, *STAT1*, *TAP1*, and *TAP2*) in both nasal mucosal and tracheal tissue. KEGG pathway analysis supported this observation, revealing enrichment of several antiviral and immune-related pathways at 4 dpi, including pathways such as antigen processing, Toll-like receptor signaling, and influenza A. This elevated response at 4 dpi may reflect altered viral clearance dynamics in obese minipigs, suggesting differences in the kinetics of antiviral responses in respiratory tissues compared to lean animals. Such a response may represent altered regulation of immune responses in obesity, potentially contributing to differences in viral clearance and tissue immune activation. Evidence from primary human nasal epithelial cells from obese individuals supports this interpretation, showing more pronounced inflammation and tissue injury following IAV infection compared to lean controls, with elevated levels of inflammatory mediators such as C–C motif chemokine ligand 20 (CCL20), plasminogen activator inhibitor-1 (PAI-1), interleukin 8 (IL-8), interleukin 1 beta (IL-1β), and IFN-β.^28^ The altered antiviral immune response observed by tracheal transcriptomics was accompanied by the presence of immune cell infiltration on day 4 after infection in two out of four of the obese pigs, whereas such infiltration was not observed in the lean group 4 dpi.

Histopathological examination of the lungs revealed immune cell infiltration in one pig in the lean-IAV group showing mild neutrophil exudation in the bronchi and bronchioles at 4 dpi, while alveolar macrophages were observed in three out of four obese-IAV pigs. No corresponding differences in immune gene expression were found in the lung of either the obese-IAV or lean-IAV group 4 dpi. This suggests that the observed cellular changes may not directly reflect changes of the transcriptional level at this time point, potentially reflecting differences in the temporal dynamics of cellular infiltration and gene expression. A previous study in a diet-induced obese mouse model, reported altered timing of mononuclear cell recruitment in the lungs after IAV infection, including delayed cell infiltration.^14^ Similar temporal differences have also been described in mouse studies where obesity influences the kinetics of immune cell infiltration.^13^^,^^15^^,^^22^ Together these studies, along with the present findings, suggest that obesity might be associated with altered timing of pulmonary immune responses following IAV infection. Importantly, histopathological analysis was performed on a subset of randomly selected animals (four pigs from each condition). While random selection was applied, this limited sample size should be taken into consideration when interpreting the histological findings. An increase in serum CRP, a key indicator of inflammation, following IAV infection in both lean and obese pigs relative to their mock infected counterparts indicates that influenza infection triggered an inflammatory response, although the magnitude of this response differed between the diet groups. The combination of high-fat diet and influenza yielded the highest absolute CRP concentrations, suggesting an enhanced overall inflammatory burden, supporting the notion that a high-fat diet may amplify inflammatory responses to viral infection. This is in line with other experimental models showing that high-fat diet or obesity exacerbate influenza-induced inflammation and pathology.^29^

In summary, this first study of airway responses to IAV in pigs with obesity-induced meta-inflammation reveals the complexity of immune dynamics in obesity, where the immune system may appear normal at baseline but respond differently under stress, including respiratory infection. Using a translational large-animal model, the findings show that obesity-associated meta-inflammation modulates antiviral and inflammatory responses during IAV infection, with differential gene expression patterns likely influenced by altered viral load and response kinetics in lean versus obese hosts. Although only minor differences in antiviral gene expression, protein abundance, and immune cell infiltration were observed in obese pigs prior to infection, IAV challenge in the obese context appears to be associated with differences in antiviral and inflammatory responses, as well as viral clearance efficiency. These results highlight how host immunity is reshaped in obesity with meta-inflammation and may help explain increased susceptibility and severity of respiratory viral infections in humans.^3^^,^^4^^,^^5^^,^^6^^,^^12^^,^^33^^,^^34^^,^^35^^,^^36^

### Limitations of the study

It is crucial to acknowledge that the antiviral and inflammatory response is influenced by multiple factors, including host species, sex differences, viral strain, time of sampling, and tissue sampling site. These variables may contribute to variability in immune responses across different studies and model systems, underscoring the complexity of host immune reactions during viral infections. To comprehensively characterize tissue-resident and infiltrating immune cells before and after infection in the context of meta-inflammation, single-cell transcriptomics would be a valuable approach to study local respiratory cell dynamics in greater detail. A further limitation of the present study is that tissue characterization was performed at a single post-infection time point (4 dpi). Consequently, the temporal dynamics of viral replication, immune cell recruitment, and inflammatory response could not be addressed. Future studies of obese pigs should include earlier time points to better characterize the kinetics of innate antiviral immunity and explore viral replication and evolution, as demonstrated in mouse and ferret studies.^30^^,^^31^^,^^32^ While viral RNA was higher in pigs with obesity 4 dpi it does not necessarily reflect the presence of infectious viruses, particularly at later time points when defective virus may accumulate. Measurement of infectious virus, such as TCID_50_ or plaque-forming units, would therefore have provided additional insight into viral replication and infectivity; however, this was not feasible in the present study due to suboptimal sample storage conditions. Finally, although transcriptomic analysis identified pathways and gene expression patterns consistent with altered antiviral and inflammatory responses in obesity, the proposed mechanistic interpretations are based on transcriptomic associations and require further functional validation to confirm their biological relevance.

While Göttingen minipigs are a relatively genetically homogeneous breed of pigs compared to conventional outbred pigs, they are not fully inbred, and biological variability between individuals remains evident. Such differences may reflect variability in the results shown and mirror the heterogeneity seen in human populations. Finally, the relatively small number of animals in the mock groups (*n* = 4 per group) represents a potential limitation, and including more controls in future studies could help further strengthen statistical power and confidence in the findings.

## Resource availability

### Lead contact

Requests for further information and resources should be directed to and will be fulfilled by the lead contact, Betina Lyngfeldt Henriksen (blyhen@dtu.dk).

### Materials availability

This study did not generate new unique reagents.

### Data and code availability

• RNA sequencing data have been deposited at Gene Expression Omnibus (GEO): GSE319516 and are publicly available as of the date of publication. Proteomics data have been deposited at ProteomeXchange Consortium via the PRIDE partner repository: PXD074506 and are publicly available as of the date of publication.
• All other data reported in this paper will be shared by the lead contact upon request.
• This paper does not report original code.
• Any additional information required to reanalyze the data reported in this paper is available from the lead contact upon request.

## Acknowledgments

The authors thank the technicians and students for their technical contributions in the laboratory. The authors further thank all staff involved in the animal experiment at Lindholm. The work presented in this article is supported by the Independent Research Fund Denmark, Technology and Production Sciences (grant no. 3105-00073B), and by the FluZooMark project funded by the 10.13039/501100009708Novo Nordisk Foundation (grant no. NNF19OC0056326).

## Author contributions

Conceptualization, K.S. and S.M.R.S.; animal experiment, K.S., S.M.R.S., L.L., and L.E.L.; methodology, B.L.H., S.M.R.S., P.M.H.H., and K.S.; analysis, B.L.H., S.M.R.S., L.B., C.K., S.W., and A.S.; visualization, B.L.H., L.B., and K.S.; writing – original draft, B.L.H. and S.M.R.S.; writing – review and editing, B.L.H., S.M.R.S., L.B., C.K., S.W., L.L., A.S., H.E.J., L.E.L., P.M.H.H., and K.S.; funding acquisition, K.S.; supervision, K.S. and P.M.H.H.; project administration, K.S.

## Declaration of interests

The authors declare no competing interests.

## STAR★Methods

### Key resources table

**Table:** 

REAGENT or RESOURCE	SOURCE	IDENTIFIER
**Antibodies**		

Rabbit anti-human CRP antibody	DAKO	A073
HRP-conjugated goat anti-rabbit IgG	DAKO	P217

**Bacterial and virus strains**		

Influenza A virus, A/swine/Denmark/12687/2003 (H1N2)	Statens Serum Institut (SSI), Denmark	N/A

**Chemicals, peptides, and recombinant proteins**		

cOmplete™ Mini EDTA-free protease inhibitor cocktail tablet	Roche	Cat: 04693159001
BCA Rapid Gold assay	Thermo Fischer Scientific	Cat: A55860
LysC	Wako	Cat: 125-05061
Trypsin	Promega	Cat: V5280

**Critical commercial assays**		

miRNeasy Mini Kit	Qiagen	Cat: 217604
SensiFast Probe No-ROX One-Step Mix kit	Meridian Bioscience	Cat: BIO-76005
QuantiTect Reverse Transcription Kit	Qiagen	Cat: 205314
TagMan PreAmp Master Mix	Applied Biosystems	Cat: 4384267

**Deposited data**		

RNA-seq data	This paper	GEO: GSE319516
Proteomics data	This paper	PRIDE: PXD074506

**Experimental models: Organisms/strains**		

Göttingen minipigs	Ellegaard Göttingen minipigs A/S	N/A

**Oligonucleotides**		

Primers and probe for IAV: RimF (5′-CTT CTA ACC GAG GTC GAA ACG-3′), RimR (5′-AGG GCA TTT TGG ACA AAK CGT CTA-3′) and MAprobeDL (5′-FAM CCC AGT GAG CGA GGA CTG CAG CGT BHQ-1-3′)	This paper	N/A
Primers for host gene expression see Tables S2, S9, and S10	This paper	N/A

**Software and algorithms**		

Dr. Tom Visualisation Solution	BGI Genomics	https://www.bgi.com/global/service/dr-tom
Spectronaut^TM^ (version 18.6)	BOPGNOSYS	https://biognosys.com/software/spectronaut/
Fluidigm Real-Time PCR Analysis software 4.7.1	Standard Biotools	https://standardbio.com/products-services/software/
GenEx7 Pro software	MultiD Analysis AB	https://multid.se/product/genex/

### Experimental model and study participant details

#### Animals

The study was approved by the Animal Experiments Inspectorate, Ministry of Food, Agriculture and Fisheries in Denmark (permit number 2016-15-0201-01022).

Castrated nine weeks old Göttingen Minipigs (*n* = 26) were randomized into two diet groups and fed a diet high in fat, fructose, and cholesterol (*n* = 13) (Ossabaw pig diet – 5B4L, TestDiet, Table S6) or standard minipig chow (SD, *n* = 13) (Mini-pig, Special Diets Services, Table S6) (Figure 1) with free access to water and were housed on a 12-h light/12-h dark cycle.^16^ The obese pigs were fed 2.5–3.0% of the group’s average body weight, with the ration adjusted weekly based on body weight measurements.

Ninety-nine days after initiation of the feeding (henceforth termed 0 dpi), pigs from both the obese(*n* = 9) and lean (*n* = 9) groups (designated obese-IAV and lean-IAV, respectively) were inoculated with 2 ml/nostril of 1.6x10^8^ TCID_50_/ml A/swine/Denmark/12687/2003 (H1N2) IAV using a mucosal atomization device (Mad Nasal Intranasal Mucosal Atomization Devices (Teleflex). Prior to inoculation, all pigs were sedated via intramuscular injection.^37^ Groups of obese (*n* = 4) and lean (*n* = 4) pigs (designated obese-mock and lean-mock, respectively) were inoculated with 2 ml/nostril virus-free growth medium. Blood and nasopharyngeal swab samples were collected daily from 0 to 4 dpi (Figure 1A). At 4 dpi, all 26 pigs were euthanized, and respiratory tissue samples (nasal mucosa, upper trachea, lower trachea, and lung) were collected (Figure 1B).

### Method details

#### RNA extraction

Total RNA from nasal mucosa, trachea, and lung tissue was extracted using a gentleMACS Dissociator (Miltenyi Biotec) for homogenization and miRNeasy mini Kit (Qiagen) for extraction according to the manufacturer’s protocol.

Total RNA was extracted from nasopharyngeal FLOQswab samples (COPAN) using the Oragene RNA RE-100 purification protocol (DNA Genotek) and the RNeasy Micro Kit (Qiagen). The nasopharyngeal samples were incubated in a 50°C water bath for one hour and 550 μL sample was aliquoted to a microcentrifuge tube. During elution the eluate was run through the membrane twice to enhance the RNA yield.

#### Quantification of IAV

An adapted version of an RT-qPCR assay was used to detect IAV by targeting the matrix (M) gene, employing the SensiFast Probe No-ROX One-Step Mix kit (Meridian Bioscience).^37^ The primers RimF (5′-CTT CTA ACC GAG GTC GAA ACG-3′) and RimR (5′-AGG GCA TTT TGG ACA AAK CGT CTA-3′) with the probe MAprobeDL (5′-FAM CCC AGT GAG CGA GGA CTG CAG CGT BHQ-1-3′) were used. Each reaction had a final volume of 25 μL and contained 3 μL of RNA template, 13 μL of SensiFAST Probe No-ROX One-Step Mix (Meridian Bioscience), 0.55 μL of RiboSafe RNase Inhibitor (Meridian Bioscience), 0.28 μL of reverse transcriptase (Meridian Bioscience), 7 μL of RNase-free water, and 2 μL of primer–probe mix consisting of 0.08 μL of each primer (0.4 μM final concentration), 0.06 μL of probe (0.3 μM final concentration), and 1.78 μL of RNase-free water. Amplification was performed on a Rotor-Gene Q platform (QIAGEN) with the following conditions: reverse transcription at 50°C for 30 min, initial denaturation at 95°C for 2 min, followed by 40 cycles of 95°C for 15 s, 55°C for 15 s, and 72°C for 20 s.

#### RNA-seq data analysis

RNA-seq libraries were constructed with a non-stranded PolyA selection method according to the manufacturer’s specifications (BGI Genomics).^38^ Clean reads were mapped to the pig genome (reference genome version GCF_000003025.6_Sscrofa11.1). BGI Genomics visualization tool Dr. Tom was used for data analysis and visualization.^39^

Approximately 2.2 billion clean reads were generated in the RNA-seq analysis of trachea with an average of 89.2 million (88.8-92.8 million) acquired for each sample. Across the samples, 98-98.87% of the clean reads were mapped to the porcine genome and 92.3-95.72% of the clean reads were uniquely mapped (mapped to a unique location on the reference genome) (Table S7). All DEGs identified and their expression levels can be found in Table S1. One sample of the lean-IAV group was degraded (RNA integrity of 2.4) and not included for the RNA-seq data analysis. The RNA sequencing data have been deposited in the Gene Expression Omnibus (GEO) under the accession number GSE319516.

#### cDNA synthesis, pre-amplification, and exonuclease treatment

Complementary DNA (cDNA) was synthesized in duplicates from 300 ng total RNA for nasopharyngeal swab samples and from 500 ng total RNA for respiratory tissues using QuantiTect Reverse Transcription Kit (Qiagen) according to the manufacturer’s protocol. Samples and –RT controls (cDNA synthesis without reverse transcriptase) were pre-amplified using TagMan PreAmp Master Mix (Applied Biosystems) according to the manufacturer’s protocol^16^ applying 19 cycles of amplification for respiratory tissue and 22 cycles for nasopharyngeal swabs.

#### High-throughput qPCR

qPCR analysis was performed using two panels targeting 96 and 24 genes, respectively, each including 3–5 candidate reference genes (Tables S8, S9, and S10). The 96-primer panel was used to validate RNA-seq results in the lower trachea and for the nasopharyngeal swab samples from 0-4 dpi, while the 24-primer panel was based on RNA-seq data and applied to nasal mucosa, upper trachea, and lung tissue at 4 dpi. The assays were performed on a BioMark real-time PCR platform (Standard Biotools) using 96.96 and 192.24 Dynamic Array Integrated Fluid Circuit chips, following the manufacturer’s specifications.

The qPCR efficiency and dynamic range for each primer pair were determined using serial dilutions prepared from separate pools for nasal mucosa, upper and lower trachea, and lung tissue. The qPCR data was inspected and processed using Fluidigm Real-Time PCR Analysis software 4.7.1 (Standard Biotools) and GenEx7 Pro software (MultiD Analysis AB). The quantification cycles (Cq) were corrected by the PCR efficiencies (80-120%). For each tissue, candidate reference genes were assessed for stability across all experimental groups using geNorm and NormFinder, and the most stable genes were used for normalization.^40^^,^^41^ Nasal mucosa: ribosomal protein L13a (*RPL13A*), peptidylprolyl isomerase A (*PPIA*); upper trachea: *RPL13A*, tyrosine 3-monooxygenase (*YWHAZ*); lower trachea: beta-2-microglobulin (*B2M*), *PPIA*, *YWHAZ*; lung tissue: *RPL13A*, *YWHAZ* and nasopharyngeal swab sample: actin beta (*ACTB*) and glyceraldehyde-3-phosphate dehydrogenase (*GAPDH*). Both lean-mock and obese-mock were scaled to 1, and the corresponding infectious groups were normalized relative to their respective mock controls.

#### Histopathology staining

Histological examination was performed on all eight mock-inoculated pigs as well as four randomly selected pigs from each of the IAV-inoculated groups. Tissue specimens from four pigs from each group were fixed in 10% neutral-buffered formalin, embedded in paraffin wax, sectioned into 3-4 μm and stained with hematoxylin and eosin (H&E).

#### C-reactive protein (CRP) quantification by ELISA

CRP serum concentration was determined by a indirect- ELISA using Nunc Maxisorp plates coated overnight at 4°C with CDP-coupled dendrimer (150–200 ng/mL). After washing and blocking with 5% skim milk, samples and standards were added in duplicate and incubated for 1 h at 37°C. Plates were subsequently incubated with rabbit anti-human CRP antibody (DAKO A073) followed by HRP-conjugated goat anti-rabbit IgG (DAKO P217). Signal development was performed using TMB substrate, stopped with 0.5 M sulfuric acid, and absorbance was measured at 490 nm with background correction at 650 nm.^42^ Lower limit of quantification was 1.42 mg/L.

#### Proteomics – sample preparation

The samples were homogenized in lysis buffer (6 M guanidine hydrochloride (GuHCl), 10 mM tris(2-carboxyethyl)phosphine (TCEP), 40 mM chloroacetamide (CAA), 50 mM 4-(2-hydroxyethyl)-1-piperazineethanesulfonic acid (HEPES), pH 8.5, with cOmplete™ Mini EDTA-free protease inhibitor cocktail) using a TissueLyser II (Agilent) and one steel bead twice for one minute at 1–30 Hz. The samples were incubated for 5 min at 95°C, and lysates were precipitated in acetone overnight at -20°C, centrifuged for 20 min at 4000x g at 4°C, and resuspended in lysis buffer. Protein concentration was determined with the BCA Rapid Gold assay (Thermo Fischer Scientific) and 20 ug were taken for digest. Samples were diluted 1:3 with 10% Acetonitrile, 50 mM HEPES pH 8.5, LysC (MS grade, Wako) was added in a 1:50 (enzyme to protein) ratio, and samples were incubated at 37°C for 4 hours. Samples were further diluted to 1:10 with 10% Acetonitrile, 50 mM HEPES pH 8.5. Trypsin (MS grade, Promega) was added in a 1:100 (enzyme to protein) ratio and samples were incubated overnight at 37°C. The samples were acidified to pH < 2 with 2% trifluoroacetic acid (TFA) and loaded onto EvoSep stage tips according to the manufacturer’s protocol.

#### Proteomics - analysis

Peptides were analysed using the pre-set ‘20 samples per day’ method on the EvoSep One instrument. Peptides were eluted over a 58-min gradient, and analysed with an Orbitrap Exploris instrument (Thermo Fisher Scientific) run in DIA mode with FAIMS ProTM Interface (ThermoFisher Scientific) with CV of -45 V. Full MS spectra were collected at a resolution of 120,000, with an AGC target of 300% or maximum injection time set to 50 ms and a scan range of 400–1350 m/z. The MS2 spectra were obtained in DIA mode in the orbitrap operating at a resolution of 15.000, with an AGC target 1000% or maximum injection time set to ‘auto’, a normalised HCD collision energy of 33. The isolation window was set to 20 m/z with a 1 m/z overlap and window placement optimization on. The cycle time for DIA scan was set to 3 s. MS performance was verified for consistency by running complex cell lysate quality control standards, and chromatography was monitored to check for reproducibility.

#### Proteomics – data analysis

The raw files were analyzed using Spectronaut^TM^^43^ (version 18.6) spectra were matched against the sus scrofa database (UP000008227). Dynamic modifications were set as Oxidation (M) and Acetyl on protein N-termini. Cysteine carbamidomethyl was set as a static modification. All results were filtered to a 1% FDR, and protein quantitation done on the M21 level. Only proteotypic peptides were included for the quantification, and protein groups were inferred by IDPicker. The mass spectrometry proteomics data have been deposited to the ProteomeXchange Consortium via the PRIDE partner repository with the dataset identifier PXD074506.

### Quantification and statistical analysis

Differences in viral RNA abundance were first tested for normality using the Shapiro–Wilk test, and homogeneity of variance was assessed with Levene’s test, after which an independent two-sample *t*-test was conducted. Mixed ANOVA was applied on serum CRP concentrations to evaluate the effect of time across the five-day sampling period. Post-hoc analyses were conducted using two-sided Welch’s *t*-tests, with *p*-values adjusted for multiple comparisons using the Benjamini-Hochberg procedure (α=0.05). Proteomics data was log_2_ transformed and checked for normality. A t-test was performed and corrected for multiple testing using Benjamini-Hochberg false discovery rate. RNA-seq data was normalized, and DEGs between groups were identified using DESeq2 with *p*-values adjusted for multiple comparisons using the Benjamini-Hochberg procedure. DEGs were defined as a *q*-value below 0.05 and with a fold change of ±1.5. qPCR data were log_2_ transformed prior to statistical analysis to meet normality assumptions. Welch’s *t*-test was conducted on qPCR results. Nasopharyngeal swab samples were analyzed by area under the curve (AUC) and Tukey Honest Significant Difference with *p*-values adjusted for multiple comparisons using the Benjamini-Hochberg procedure. Data are presented as mean ± standard error of mean (SEM) unless otherwise indicated in the corresponding figure legends. Exact sample sizes (n) and the statistical tests used for each experiment are provided in the relevant figure legends, where n represents the number of individual animals included in each analysis unless otherwise stated.
