# Supplementary material for: Obesity-associated meta-inflammation alters influenza A antiviral responses in Göttingen minipigs
Source: iScience. 2026 Aug 24;29(9):117056. doi: 10.1016/j.isci.2026.117056 (PMC13542486; doi:10.1016/j.isci.2026.117056)
Supplement: Supplementary file 1 [file mmc1.pdf]

iScience, Volume *29*

Supplemental information

Obesity-associated meta-inflammation alters influenza A antiviral responses in Göttingen minipigs

Betina Lyngfeldt Henriksen, Sofie Maiken Riisgård Starbæk, Louise Brogaard, Charlotte Kristensen, Siri Wolthoorn, Louise Lohse, Anders Stockmarr, Henrik Elvang Jensen, Lars E. Larsen, Peter Mikael Helweg Heegaard, and Kerstin Skovgaard

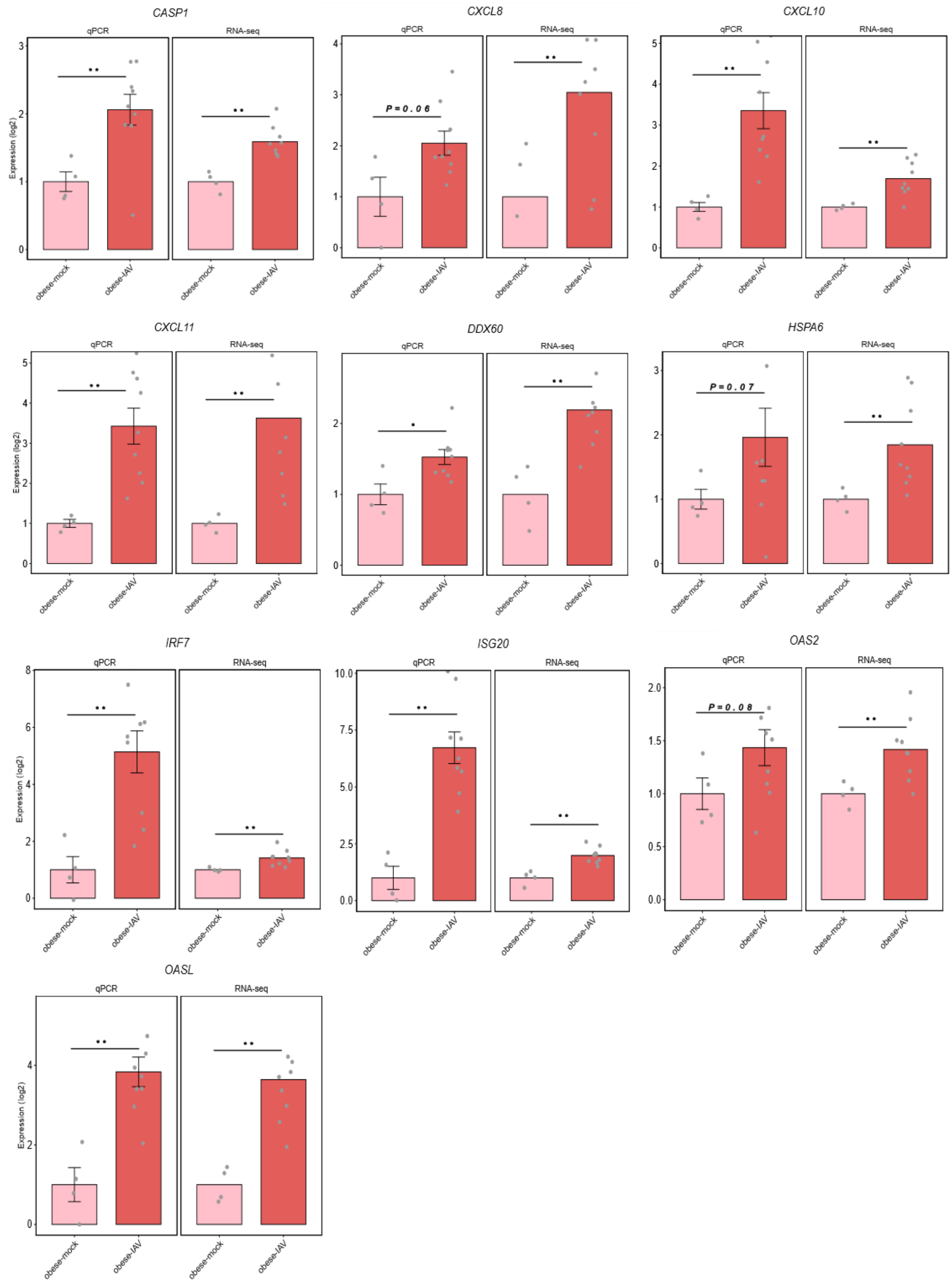

Figure S1. Validation of RNA-seq data with qPCR of lower trachea. *Validation of RNA-seq data with qPCR of antiviral immune genes only upregulated in the obese-IAV group. Log2 fold change levels estimated by RNA-seq and qPCR of selected significantly upregulated genes in the obese-IAV group compared to obese-mock group. Obese-mock was scaled to 1, and the obese-IAV group was normalized relative to obese-mock, for both qPCR and RNA-seq analyses. Mean value ± SEM. n = 4 (Lean-Mock), n = 4 (Obese-Mock), n = 9 (Lean-IAV), and n = 9 (Obese-IAV)*

*individual animals. RNA-seq data were normalized and DEGs were identified using DESeq2, with DEGs defined by Q-values < 0.05 and fold changes of ±1.5. qPCR statistics were performed using Welch's t-tests. *P ≤ 0.05, **P ≤ 0.01.*

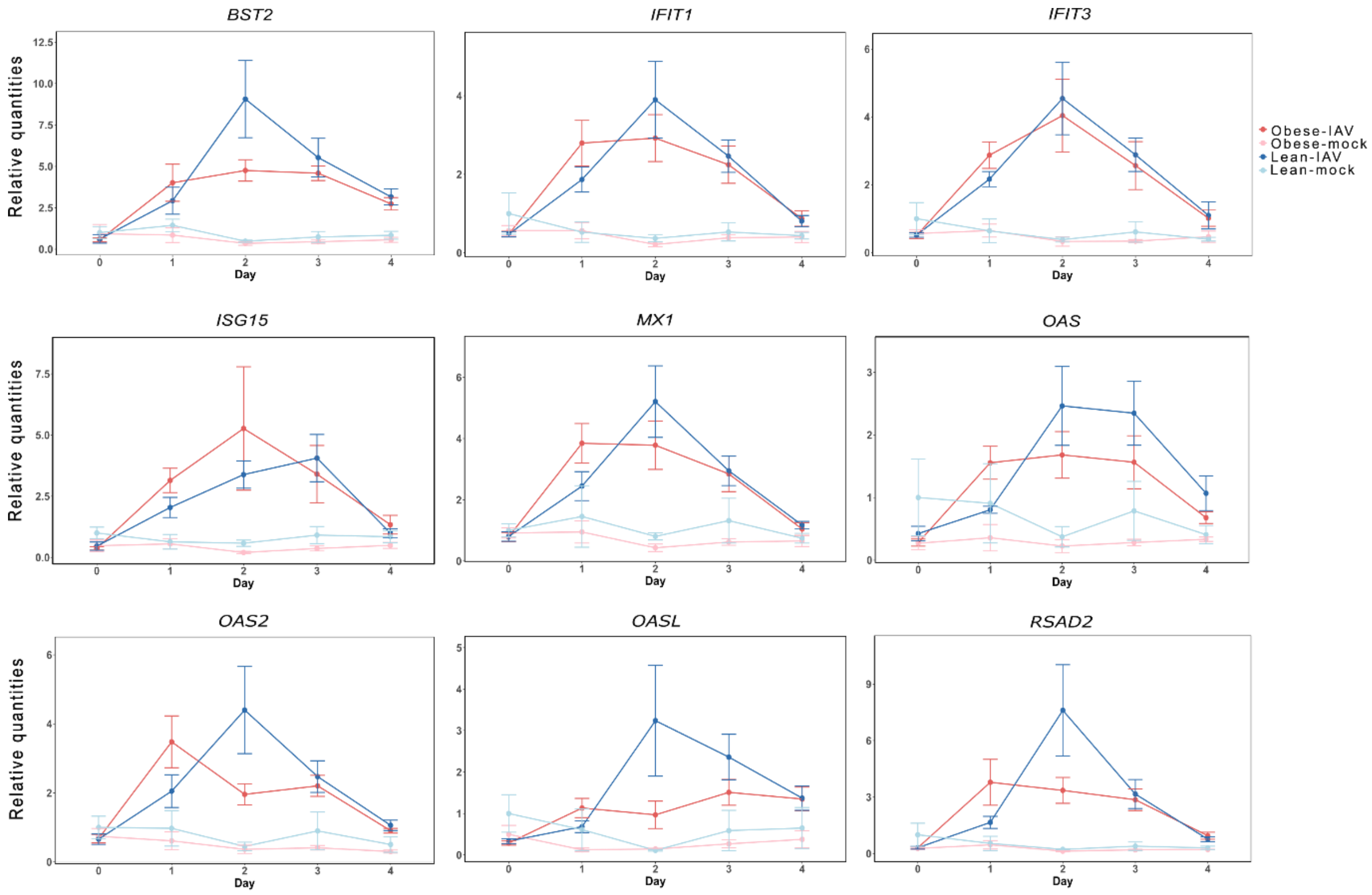

Figure S2. Nasopharyngeal swab samples collected over five consecutive days. *Statistical analysis was performed using area under the curve (AUC), showing no significant difference between the two IAV groups. The expression of each gene is scaled to the expression level in the lean-mock group on day 0. Mean value ± SEM. n = 4 (Lean-Mock), n = 4 (Obese-Mock), n = 9 (Lean-IAV), and n = 9 (Obese-IAV) individual animals. Statistics were analyzed by AUC and Tukey Honest Significant Difference with P-values adjusted for multiple comparisons using the Benjamini-Hochberg procedure.*

Table S1: Differentially expressed genes identified by RNA-seq analysis in lower trachea.

Comparison: obese-mock versus lean-mock.

**Gene ID**	**Gene Name**	**Log2 Fold Change**	**Qvalue**	**Expression**
397223	SLC22A6	21.175	9.04E-07	Up
110259967	LOC110259967	3.256	8.04E-09	Up
397628	ANGPTL4	2.537	0.006180742	Up
100152112	ABCA1	2.132	1.78E-10	Up
100620416	ABCG1	1.913	2.59E-12	Up
399527	CPT1A	1.149	0.036635029	Up
450213	RXFP4	0.988	0.021895264	Up
100512775	ST6GAL2	0.979	6.21E-06	Up
100627480	TFF3	0.915	0.026611467	Up
100739447	NALCN	0.763	0.001453461	Up
100626975	BHLHA15	0.713	3.11E-11	Up
100155108	THBS3	-0.606	0.013271632	Down
494561	HK2	-0.721	0.003426174	Down
396675	SREBF2	-0.835	1.47E-10	Down
100517694	FAM221B	-0.897	0.0049207	Down
100144446	HMGCR	-0.908	1.79E-05	Down
100625143	ASIC3	-0.910	0.0135685	Down
100152303	ACAT2	-1.117	9.43E-04	Down
100511161	PLA2G3	-1.252	7.27E-05	Down
403162	INSIG1	-1.288	5.43E-07	Down
100516518	FAM24B	-1.372	0.036226345	Down
100233203	STARD4	-1.440	0.026611467	Down
100156500	DPYS	-1.655	1.21E-04	Down
396801	LDLR	-1.888	1.18E-06	Down
100155736	LIPG	-1.890	2.71E-04	Down
396870	PPBP	-3.063	0.021895264	Down
100523745	LOC100523745	-6.135	1.62E-67	Down

Comparison: lean-IAV versus lean-mock

Gene ID	Gene Name	Log2 Fold Change	Qvalue	Expression
100736865	LOC100736865	10.347	0.010338	Up
397417	MT1A	3.203	2.11E-05	Up
100154311	TCHHL1	3.195	0.0330002	Up
100627361	RIPPLY1	2.720	0.0089525	Up
100518612	MFSD2A	1.993	0.0106107	Up
100526075	ALDH3A1	1.903	4.46E-05	Up
110261124	IFI6	1.810	0.015133	Up
100037920	MT1D	1.807	0.0312274	Up
100628141	MS4A7	1.776	0.0089335	Up
110256043	LOC110256043	1.763	0.0015909	Up
100154609	PER2	1.693	2.42E-24	Up
397102	AMBN	1.641	0.0102327	Up
100156780	SDF2L1	1.623	8.34E-06	Up
493186	SOCS3	1.568	0.019529	Up
100217376	ANGPTL7	1.561	0.0363084	Up
450213	RXFP4	1.428	1.70E-05	Up
100524723	CHAC1	1.344	0.0159401	Up
100518544	IFITM3	1.334	0.0167529	Up
100514482	AGR2	1.316	2.11E-05	Up
110255948	LGALS3BP	1.255	0.0139213	Up
100519581	GJB1	1.229	0.0016985	Up
100307052	SOCS1	1.216	0.0492166	Up
100144486	FOS	1.206	0.0330073	Up
100626975	BHLHA15	1.175	2.66E-09	Up
100525292	CTPS1	1.132	0.0103269	Up
100519082	LOC100519082	1.131	0.010338	Up
100627227	NPM3	1.113	0.0238558	Up
100514873	BHLHE40	1.109	0.0441688	Up
100155947	DERL3	1.060	6.82E-06	Up
100627898	PER3	1.011	0.0047166	Up
100155423	FKBP5	0.993	0.0095782	Up
100520025	CRELD2	0.991	0.0016919	Up
397052	SULT1E1	0.986	0.0100196	Up
100737352	ACKR2	0.986	0.0131946	Up
100517321	TSC22D3	0.977	4.68E-05	Up
110255272	LOC110255272	0.971	0.0128562	Up
100505460	MANF	0.939	0.0012687	Up
100524972	LOC100524972	0.936	0.0024552	Up
110260059	CYS1	0.929	0.0046476	Up
100519648	AZGP1	0.926	0.003747	Up
100155573	YDJC	0.918	0.0034919	Up
100521593	DNAJB9	0.910	5.42E-07	Up
100520131	OSR1	0.903	0.0174279	Up

100126278	AQP5	0.899	3.23E-04	Up
100515246	TPD52L1	0.887	0.0011831	Up
100624915	MCM5	0.885	0.028344	Up
100153104	POLH	0.859	3.57E-07	Up
100312961	ASNS	0.840	0.0418454	Up
100514361	PDSS1	0.838	0.0051075	Up
100625290	ZBTB16	0.836	0.0208961	Up
100516395	LPO	0.829	0.0211562	Up
100157816	MTUS2	0.824	0.0081772	Up
100513456	TSPAN4	0.794	2.19E-11	Up
100155615	AVPI1	0.787	0.0010318	Up
100516870	HYOU1	0.781	0.0010318	Up
100517749	FEN1	0.776	0.002524	Up
100135031	SLA-2	0.772	0.0456812	Up
100523323	NOX4	0.769	0.0198337	Up
100627480	TFF3	0.765	1.23E-04	Up
100627558	EMG1	0.762	0.0078247	Up
100522795	REV1	0.758	3.05E-21	Up
100522112	GCNT3	0.750	0.0047166	Up
100517750	CRY2	0.737	6.26E-04	Up
100624785	LOC100624785	0.733	0.0451865	Up
100515902	LOC100515902	0.728	0.0489317	Up
100524304	FARSA	0.715	2.54E-04	Up
100156419	HOMER2	0.713	0.0040707	Up
100155282	PLAC9	0.709	0.0082208	Up
100620715	WDR4	0.708	0.0492166	Up
100155706	KHDRBS3	0.693	0.0204423	Up
100154112	TIGAR	0.692	0.0139287	Up
100511101	TAC4	0.687	0.0040707	Up
100152857	CCDC167	0.683	0.0131946	Up
397081	KCNN4	0.679	0.0322555	Up
100621588	FICD	0.676	0.0363084	Up
100154166	DNAJC3	0.667	0.0168407	Up
100522203	PDIA4	0.665	4.65E-05	Up
407060	HSPA5	0.663	0.0099942	Up
100621392	GALE	0.655	0.0030972	Up
780426	CCND3	0.654	1.73E-09	Up
100515505	MPP2	0.650	0.0331678	Up
100529254	EIF4EBP3	0.647	0.0010318	Up
102157990	DHODH	0.646	0.0022176	Up
100522734	PREB	0.643	0.0024221	Up
100525039	MCRIP2	0.641	0.0094964	Up
110257107	MCM6	0.638	0.0477255	Up
100524525	POMGNT2	0.633	1.23E-04	Up
100525876	LOC100525876	0.632	9.04E-05	Up
100158162	ATP10A	0.631	0.03174	Up

100521487	WEE1	0.630	1.23E-04	Up
654406	AKR1CL1	0.626	0.0352179	Up
100151836	SELENOS	0.617	8.17E-06	Up
100523046	C2H19orf24	0.616	3.33E-04	Up
100515229	PHTF2	0.611	0.002524	Up
100526092	CDK2AP2	0.607	0.0091639	Up
100518264	PIGY	0.606	0.0225492	Up
101154685	PYURF	0.606	0.0225492	Up
100519708	SH2B2	0.597	0.0428103	Up
100621140	YARS	0.597	0.0286214	Up
100152894	DNAJB11	0.597	0.0131047	Up
100155141	EEF1E1	0.595	0.017849	Up
595107	NIP7	0.588	0.0174041	Up
414385	SRM	0.587	0.0087986	Up
100520161	LRRN3	-0.589	0.0277546	Down
100512602	IGSF3	-0.594	0.0235235	Down
110255248	ZNF23	-0.599	0.0122839	Down
397488	GHR	-0.604	0.0069181	Down
100511086	SYT3	-0.607	0.0492253	Down
110261428	BMF	-0.609	0.0047166	Down
100520165	TMEM164	-0.611	0.0258686	Down
100523290	CYFIP2	-0.611	0.0487362	Down
397471	MMP14	-0.620	0.0077763	Down
100520318	TUB	-0.629	0.028344	Down
100626628	GIPR	-0.630	0.0093189	Down
100512979	GRAMD1B	-0.632	0.0109544	Down
100627921	PORCN	-0.632	0.010338	Down
100512589	ZNF608	-0.633	0.0454302	Down
100625833	DSG2	-0.638	0.0444573	Down
102158480	KIF3C	-0.643	0.0069181	Down
100514238	ERICH3	-0.645	0.0441688	Down
100512575	ARHGEF9	-0.646	0.0384785	Down
100522330	SLC7A8	-0.647	0.040013	Down
100519138	RETSAT	-0.651	0.0094964	Down
100513513	SLC16A2	-0.660	0.0097961	Down
100523837	PRR5L	-0.672	0.0026976	Down
106505308	PCDH9	-0.673	0.0047824	Down
397059	ITGA2B	-0.690	0.0048154	Down
397387	THRA	-0.691	0.009897	Down
100519724	MAP6	-0.693	0.0421799	Down
100170845	LPIN1	-0.702	0.0233902	Down
100519287	KLHL13	-0.704	0.0477578	Down
100521892	PTX4	-0.708	0.0412674	Down
100520441	NIPAL1	-0.709	0.0121077	Down
100157918	PLEKHS1	-0.717	8.54E-04	Down
100624829	APH1B	-0.722	2.61E-04	Down

100525059	HHIP	-0.739	0.0050408	Down
100511336	PROM1	-0.777	0.0084826	Down
100156973	L3MBTL1	-0.806	0.041887	Down
110258064	CCDC187	-0.814	0.0494862	Down
100157049	PSD	-0.820	0.047068	Down
100512515	AFF2	-0.820	2.88E-04	Down
100154280	SCML4	-0.828	0.0441688	Down
100521009	SLC4A8	-0.843	0.0314937	Down
102161907	CROCC2	-0.881	0.0367084	Down
100152653	ART4	-0.918	0.010338	Down
100158140	CFAP46	-0.920	0.0362086	Down
100520976	SLC13A5	-0.924	0.0476883	Down
100620608	LEXM	-0.939	0.0287112	Down
100517547	BCO2	-0.966	0.0411719	Down
100511543	SYTL5	-0.976	0.0049697	Down
100737771	CDKL2	-0.996	0.0089525	Down
397433	KCNB1	-1.016	0.0043685	Down
100522421	LOC100522421	-1.025	0.0110224	Down
100521893	NPAS2	-1.033	3.23E-04	Down
100514167	ZNF19	-1.060	0.0050123	Down
100519689	LRP2	-1.065	0.015133	Down
100517738	KCP	-1.083	5.49E-04	Down
110259328	LOC110259328	-1.095	0.0391681	Down
100286800	BPI	-1.101	9.72E-04	Down
102164126	ZNF510	-1.134	0.0016603	Down
100511845	TXLNB	-1.173	0.002023	Down
110258749	APLNR	-1.231	0.0040707	Down
100153591	SMIM10	-1.232	0.008339	Down
100515456	TRIL	-1.279	0.0174041	Down
397193	GUCY2C	-1.300	0.0186413	Down
102165484	LOC102165484	-1.310	0.0352179	Down
100312970	ELOVL6	-1.317	0.003304	Down
397154	CHGB	-1.340	0.0198337	Down
100037286	ARNTL	-1.384	1.07E-04	Down
100302018	CLDN15	-1.391	0.0071145	Down
106507829	COLCA1	-1.396	0.0174041	Down
100518270	C9H1orf186	-1.422	0.0019597	Down
397538	ME1	-1.456	0.0287112	Down
100525616	AOX2	-1.472	0.0010318	Down
100303611	LCN6	-1.568	0.0121747	Down
110257816	LOC110257816	-1.578	0.0480067	Down
100152957	EPB42	-1.627	0.0398711	Down
414373	CCR3	-1.650	0.0347696	Down
399532	FABP3	-1.730	0.0131946	Down
100520309	ABCD2	-1.922	0.0490648	Down
100126283	AQP7	-1.939	0.0388012	Down

100144303	ACE2	-2.001	0.0016603	Down
102161780	LOC102161780	-2.031	0.0118805	Down
100134983	TUBB1	-2.132	0.0016603	Down
397660	ADIPOQ	-2.234	0.04531	Down
100270723	RBP7	-2.539	0.0109544	Down
553951	CCL20	-2.555	0.0398711	Down
100156756	ACVR1C	-2.656	0.0174041	Down
641347	SLC34A2	-2.687	0.0250094	Down
100158011	LOC100158011	-2.709	0.0013662	Down
396971	ALOX15	-2.751	5.49E-04	Down
102164536	AHSP	-2.926	0.0010318	Down
100157564	CEBPE	-2.977	0.0238558	Down
100520680	LOC100520680	-3.232	4.68E-05	Down
100511431	CRB2	-3.592	0.0078452	Down
100154756	TREML1	-4.316	0.0010449	Down
100621400	LOC100621400	-23.898	1.37E-10	Down

Comparison: obese-IAV versus obese-mock

Gene ID	Gene Name	Log2 Fold Change	Qvalue	Expression
100153042	TGM3	6.445	1.80E-04	Up
100524951	ACOD1	4.765	0.00882583	Up
595119	OASL	4.580	6.49E-05	Up
100523745	LOC100523745	4.551	0.01438896	Up
110259942	ZG16B	4.151	0.00553984	Up
494019	CXCL10	4.139	6.02E-04	Up
100169744	CXCL11	4.109	0.00131999	Up
100517436	AOC1	3.738	0.01733166	Up
100155195	LOC100155195	3.325	0.00429256	Up
397644	CD180	3.241	0.01675776	Up
448855	ISG20	3.239	2.00E-05	Up
100151938	GBP1	3.231	0.01238075	Up
397417	MT1A	3.150	0.00479893	Up
100154248	IFIT3	2.926	0.02871066	Up
100515875	BATF2	2.895	0.0010453	Up
100135681	CXCL9	2.883	0.00248482	Up
100155467	IFIT2	2.822	0.01307102	Up
100522045	HAVCR2	2.650	3.35E-04	Up
100512797	GVIN1	2.648	0.02292866	Up
110255360	LOC110255360	2.620	0.0199086	Up
396906	HSPA6	2.607	0.03665564	Up
100158037	DDX60	2.602	0.0012934	Up
100125829	MUC13	2.525	2.76E-10	Up
100158219	FAM26F	2.505	0.01532078	Up
100156210	ETV7	2.445	0.00301255	Up
100511370	HSH2D	2.356	6.23E-04	Up
595128	OAS2	2.350	0.0389136	Up
100517427	LOC100517427	2.344	0.01854037	Up
100038026	TNFSF13B	2.323	0.01023301	Up
100153038	IFIT1	2.300	0.04768124	Up
100515259	XDH	2.297	0.03431352	Up
397443	HPD	2.266	0.02908358	Up
100514753	UBE2L6	2.146	0.01564391	Up
100153948	PARP14	2.125	0.00300721	Up
100037289	IRF7	2.119	0.01564391	Up
574058	CD274	2.116	0.01733166	Up
110261124	IFI6	2.101	0.0010453	Up
397508	SELE	2.082	4.31E-04	Up
100523310	LOC100523310	2.080	0.02799574	Up
396611	IRF1	2.074	0.00783524	Up
100153902	ISG12(A)	2.069	4.17E-04	Up
110256385	RTP4	2.069	0.00355172	Up
102159947	XAF1	2.056	0.01023301	Up

100623615	LY6D	2.048	0.00133876	Up
100625050	EPSTI1	2.044	0.03547449	Up
100037944	CHI3L2	2.040	0.03181875	Up
100519877	IDO1	2.007	0.01720748	Up
100628141	MS4A7	2.005	0.01833151	Up
100515390	WARS	1.985	0.02369536	Up
100517204	TFEC	1.968	0.02206246	Up
397319	CASP1	1.960	4.35E-04	Up
110255361	LOC110255361	1.940	0.030783	Up
396880	CXCL8	1.895	0.04883596	Up
100625890	SLFN11	1.877	0.02889276	Up
100525175	PLAC8	1.863	0.00726704	Up
100623872	CMPK2	1.856	0.04761115	Up
396777	USP18	1.793	0.02398019	Up
654294	PSMB9	1.793	0.02686749	Up
100738704	TNIP3	1.780	0.00646064	Up
397031	CD163	1.760	0.03194779	Up
100155736	LIPG	1.757	0.01507487	Up
100620339	CSF2RA	1.742	0.00613729	Up
100514958	SLAMF8	1.742	0.00554887	Up
733649	TAP1	1.711	0.03370594	Up
100152279	GPR84	1.695	0.00919831	Up
100038025	CLEC7A	1.682	1.60E-04	Up
100519098	LOC100519098	1.680	0.04883596	Up
406191	TNFSF10	1.655	0.04883596	Up
100524766	P2RY13	1.655	0.03635583	Up
100522887	LOC100522887	1.655	0.03233791	Up
100525523	IFI44	1.636	0.01539886	Up
733692	IL15RA	1.625	0.04602684	Up
106505631	LOC106505631	1.623	0.03011006	Up
100520459	DTX3L	1.553	0.03431352	Up
100512849	P2RY6	1.550	0.03530413	Up
396972	LGALS9	1.543	0.030783	Up
445467	C4A	1.537	0.00884656	Up
100135667	NLRC5	1.527	0.02100199	Up
100626247	LOC100626247	1.526	0.01496029	Up
396655	STAT1	1.518	0.02141519	Up
100625290	ZBTB16	1.517	0.04045942	Up
100155423	FKBP5	1.499	0.03657159	Up
397441	CD86	1.448	0.02148188	Up
100154624	CLCA4	1.392	8.53E-04	Up
100519082	LOC100519082	1.362	0.01852265	Up
106504168	FBXO39	1.339	0.03431352	Up
396975	PSMB8	1.330	0.02956427	Up
100519390	PARP9	1.327	0.03431352	Up
733650	TAP2	1.311	0.0389136	Up

100516832	B3GALT5	1.310	6.02E-04	Up
396648	HSP70.2	1.310	0.02660618	Up
448981	C2	1.234	0.01206996	Up
733625	SFN	1.229	6.66E-04	Up
100037296	TLR7	1.203	0.03530413	Up
100514901	BCL2L14	1.200	0.02983401	Up
100152823	TTLL11	1.187	0.00526013	Up
100156073	LOC100156073	1.170	0.01507487	Up
100302538	TRIM21	1.166	0.03547019	Up
100157298	SAMSN1	1.166	0.03370594	Up
100519843	MLC1	1.156	0.01909401	Up
100520491	LOC100520491	1.111	0.01064183	Up
100738193	BCL3	1.098	0.04873014	Up
106507531	C1RL	1.082	9.98E-04	Up
100737047	SDR42E2	1.081	0.02329608	Up
110255988	MILR1	1.077	0.02619075	Up
100524972	LOC100524972	1.067	0.02372971	Up
397033	gene-B2M-2	1.040	7.79E-05	Up
100152236	CPNE9	1.036	0.0237545	Up
100153090	CTSS	1.021	0.00974263	Up
110255236	B2M	0.961	0.00145871	Up
100156894	ASPHD2	0.941	4.63E-11	Up
780415	IRF9	0.937	0.0080567	Up
100520808	ARHGAP15	0.919	0.04933484	Up
100622289	BSPRY	0.906	0.01012066	Up
100135031	SLA-2	0.898	0.01349031	Up
397139	FUT2	0.897	0.00307629	Up
100048931	HSPH1	0.897	0.01067529	Up
100521564	ARAP2	0.815	0.00882583	Up
100037293	SLA-1	0.812	0.04768124	Up
100037288	SLA-3	0.811	0.0097811	Up
100154609	PER2	0.795	0.03194779	Up
100626261	C13H3orf52	0.782	0.03181875	Up
100516870	HYOU1	0.773	0.03881054	Up
100738623	C8H4orf19	0.758	0.03657159	Up
100525936	DAPP1	0.740	0.03902352	Up
100511890	HSPA8	0.702	0.00560832	Up
397271	AIF1	0.700	0.01963616	Up
396741	DNAJB1	0.692	0.00131413	Up
100522657	AEN	0.667	0.0237545	Up
100519508	IKBKE	0.655	0.04449583	Up
100520380	CRY1	0.649	3.26E-04	Up
407060	HSPA5	0.640	5.76E-04	Up
100520422	SLC22A5	0.614	0.0423699	Up
100154166	DNAJC3	0.608	0.02956427	Up
768107	RGN	-0.598	0.04026569	Down

100524737	LOC100524737	-0.602	0.00389381	Down
100514009	LOC100514009	-0.611	0.02956427	Down
100516472	KIAA1755	-0.614	0.01602951	Down
100155219	TMED3	-0.614	0.04471935	Down
397511	NTRK3	-0.625	0.01842295	Down
100153715	TMEM44	-0.632	0.01532078	Down
100152844	SDR39U1	-0.647	0.02100199	Down
110255661	SHISA4	-0.647	0.0165607	Down
100517738	KCP	-0.685	0.03233791	Down
397640	RGS5	-0.693	0.03846389	Down
654291	SLC2A4RG	-0.696	0.03316563	Down
106508722	METTL26	-0.724	0.02214748	Down
110256708	LOC110256708	-0.736	0.0415812	Down
100519796	COPZ2	-0.762	0.02373504	Down
100515870	TYRO3	-0.800	0.00382797	Down
102164926	RHBDL1	-0.813	0.00726704	Down
406190	DNAJC6	-0.815	0.04102914	Down
100519549	EPHB2	-0.817	0.00526013	Down
100517448	CD99L2	-0.834	0.02329608	Down
494020	LMCD1	-0.836	0.00131413	Down
100520647	ANKRD55	-0.837	0.03325928	Down
449001	BAMBI	-0.840	0.02686749	Down
100513642	FAM69B	-0.847	0.0037827	Down
100620551	LXN	-0.851	0.0235254	Down
102164126	ZNF510	-0.854	0.01557122	Down
100522008	SEMA6A	-0.863	0.02292866	Down
100624044	PODXL2	-0.886	0.00974263	Down
100517574	MAPK8IP1	-0.898	0.0415812	Down
100519539	KLHL3	-0.907	9.18E-04	Down
100127154	LMO3	-0.922	0.03530413	Down
100513981	SYT17	-0.924	0.03167309	Down
100156195	CIART	-0.924	0.02292866	Down
110255210	LOC110255210	-0.931	0.01508252	Down
100286741	FSCN1	-0.933	0.04883596	Down
100153016	RERGL	-0.950	0.04761115	Down
100627698	LRRN1	-0.972	0.01169299	Down
100156043	CIB2	-0.987	0.02517002	Down
100154528	ATP13A5	-0.987	0.00560832	Down
100513315	CDK15	-1.005	0.03292982	Down
100738638	OCIAD2	-1.032	0.04883596	Down
100621076	NR1D2	-1.038	2.00E-05	Down
100216312	GP9	-1.049	0.00300721	Down
100156959	OLFM3	-1.059	0.01493713	Down
100626514	CHST2	-1.068	0.00299754	Down
100125830	KCNAB1	-1.086	0.03233791	Down
100625743	GAS2	-1.094	0.01284728	Down

106507828	LOC106507828	-1.104	0.01733166	Down
100520490	GLI1	-1.120	0.04515841	Down
100154847	CARMIL3	-1.125	0.0477924	Down
100518589	KIAA1210	-1.128	0.0415812	Down
100524747	DBP	-1.175	5.64E-04	Down
100523847	MAMSTR	-1.200	9.05E-04	Down
100512775	ST6GAL2	-1.202	9.05E-04	Down
100521763	SNAP91	-1.217	0.00546976	Down
100157228	ADAM12	-1.222	0.01023301	Down
100621879	SLC6A20	-1.263	0.00546976	Down
110258749	APLNR	-1.268	0.00485552	Down
100621701	LOC100621701	-1.288	0.01067529	Down
100526005	FEZ1	-1.291	0.01363356	Down
100523198	HEPACAM	-1.318	0.02292866	Down
100512885	CARNS1	-1.319	0.04761115	Down
100511992	NRIP2	-1.324	0.0023732	Down
100515912	TMPRSS5	-1.350	0.02956427	Down
100515642	PRRG3	-1.358	0.0415812	Down
100518861	CYGB	-1.369	0.00504729	Down
641342	NR1D1	-1.373	8.53E-04	Down
100622808	SLC26A1	-1.464	0.01551768	Down
100155151	CRABP2	-1.557	0.03846522	Down
100524882	PRSS12	-1.586	0.04626816	Down
100515947	SYNGR3	-1.586	0.03947891	Down
100621324	HSPA2	-1.605	0.01909516	Down
100152849	EMID1	-1.606	0.02398019	Down
100153933	NES	-1.614	0.03431352	Down
100622587	FNDC5	-1.632	0.00358297	Down
100521800	NCMAP	-1.656	0.02799574	Down
503661	ADORA2A	-1.668	0.04626816	Down
100624965	THSD7B	-1.707	0.04768124	Down
100519925	SEMA5B	-1.717	0.04053875	Down
100518190	MYCN	-1.752	0.03846389	Down
493772	NPPC	-1.774	0.03657159	Down
100739720	STMN4	-1.869	0.0415812	Down
100152168	FRMD5	-2.038	0.03547449	Down
100511825	UGT8	-2.041	0.04026569	Down
100513510	SCRN1	-2.061	0.01963616	Down
102163410	SHISA8	-2.071	0.01652205	Down
100624660	SH3TC2	-2.077	0.01733166	Down
100525073	ADAM11	-2.104	0.01514868	Down
100514320	DCLK3	-2.113	0.01854037	Down
100520197	VAT1L	-2.143	0.04939387	Down
100626060	SYN2	-2.145	0.03431352	Down
100737053	PPP2R2C	-2.188	0.01356128	Down
100270723	RBP7	-2.268	0.0389136	Down

100511522	C9H11orf87	-2.278	0.01733166	Down
110261233	FOXD2	-2.297	0.03642401	Down
100154381	GAS2L3	-2.306	0.02292866	Down
100523255	KANK4	-2.426	0.0017596	Down
100520760	B3GAT1	-2.440	0.01575504	Down
397497	FGF1	-2.443	0.01148698	Down
100144521	EGFL8	-2.453	0.01067529	Down
100157330	CLSTN2	-2.491	0.01317725	Down
100627126	RELN	-2.504	0.01115967	Down
100514836	PLPPR5	-2.570	0.01358277	Down
100518721	SLC25A48	-2.576	0.02890039	Down
397123	MT3	-2.576	0.01081958	Down
100155686	SORCS1	-2.578	0.01023301	Down
100520483	LRRTM1	-2.591	0.0165607	Down
397358	SLC9A3	-2.597	0.00646064	Down
100620948	GNG3	-2.634	0.019841	Down
396637	UCHL1	-2.703	0.00244678	Down
100156725	GLDN	-2.738	0.04768124	Down
100525721	LOC100525721	-2.749	0.00225049	Down
100516491	CHRNB2	-2.797	0.02613911	Down
100151989	CHRNA3	-2.845	0.02956427	Down
110256114	PMP22	-2.891	0.03007435	Down
100144495	RAB33A	-2.903	0.02979441	Down
100513608	PLEKHB1	-2.926	5.76E-04	Down
100520832	LOC100520832	-3.005	0.02950437	Down
110259967	LOC110259967	-3.030	6.34E-09	Down
100157319	SDR16C5	-3.037	0.04768124	Down
100152434	PRPH	-3.037	0.04327497	Down
396846	ZP2	-3.118	0.04808357	Down
100155358	SPTBN5	-3.156	0.04768124	Down
100627757	AATK	-3.328	0.01508252	Down
100521617	MAL	-3.410	0.02373504	Down
100145890	SFRP4	-3.425	0.01206996	Down
100511808	JAKMIP2	-3.430	0.01148698	Down
541593	FXYD2	-3.527	0.01854037	Down
100516166	CAMK2B	-3.544	0.01652205	Down
100626921	NFASC	-3.582	0.00646064	Down
641350	SNCA	-3.614	0.01397679	Down
396881	FCN2	-3.636	0.01720748	Down
100505406	DHH	-3.724	0.01675776	Down
100157279	POU3F2	-3.781	0.04272044	Down
100294682	CLDN19	-3.824	9.05E-04	Down
100524828	PEX5L	-3.973	0.00213693	Down
100626438	PRX	-3.974	0.00208611	Down
100157152	KCNQ3	-4.015	0.00485368	Down
100158245	ABCC8	-4.044	9.50E-04	Down

100156768	STMN2	-4.211	0.02889276	Down
110258341	TMEM179	-4.439	0.04761115	Down
100624679	NKAIN2	-4.461	0.03808694	Down
100511991	SMCO3	-4.532	0.01720748	Down
100156492	NEFH	-4.539	0.02306824	Down
100737002	TAGLN3	-4.607	0.01073654	Down
100519324	ETNPPL	-4.625	0.0037827	Down
397029	PLP1	-4.635	0.00429256	Down
100624431	GPR37L1	-4.709	0.0032836	Down
100623023	MLIP	-4.823	4.79E-04	Down
100525215	GJC3	-4.967	0.02956427	Down
100521224	NEFL	-4.988	0.00646064	Down
100513599	SYT1	-5.268	0.00182136	Down
100517071	DRP2	-5.481	6.53E-04	Down
414286	MBP	-6.025	3.26E-04	Down
397595	GCG	-6.297	0.01064183	Down
100153273	MPZ	-6.674	1.05E-04	Down
100628124	HAPLN2	-6.791	0.02329608	Down
100151890	PMP2	-7.587	3.30E-05	Down

Table S2. qPCR primer sequences for genes studied in nasal mucosa, upper trachea, and lung.

Gene name	Forward primer	Reverse primer	Efficiency (%)		
			Nasal mucosa	Upper trachea	Lung
CASP1	GAAGGACAAACCCAAGGTGA	TGGGCTTTCTTAATGGCATC	0.99	1.07	1.10
CD86	CATCGTCTGTGTCCTGCAAC	CACAGGTGGCTTTGCATCTA	1.14	1.09	1.06
CTSS	GTGAAAAACAGCTGGGGACT	TGGGTAAGAGGGATAATTAGCAA	0.96	1.06	1.12
CXCL10	TTCGCTGTACCTGCATCAAG	CAACATGTGGGCAAGATTGA	1.10	1.05	1.14
CXCL8	GTGGACCCCACTGTGAAAAT	CAACCTTCTGCACCCACTTT	0.97	1.02	1.11
CXCL9	AGCAGTGTTGCCTTGCTTTT	ATGCAGGAACAACGTCCATT	1.12	1.07	1.07
GBP1	TATCTTGAGACGCCCAGGAA	CCCACAGACTCCTTGGACTT	1.05	1.07	1.17
HSP70-2	CAAGATCACCATCACCAACG	TCGTCCTCCGCTTTGTACTT	1.12	1.14	1.17
HSPA6	CCAGTTGGCAGAGAAAGAGG	GGAGAAGATGGGACGACAAA	1.10	1.07	1.04
IKBKE	CATGAGACCAGGAACCACCT	AAAGCCTCAGCCAGATCCTT		1.03	1.12
IRF7	GCTCCCCACACTACACCATC	TCCAACTTCACCAGGACGA	1.18	1.09	1.11
IRF9	CATTCAGACTTGGGGAGCAG	AAAGGGGCCTCAGTGGTAAC			
ISG12A	GCCTGGCTTCCAAGATACTG	AGCCCTGCTGAAGGAAGTC	1.17	1.11	1.19
OAS2	GATAATTCGTGCTGGCACCT	GATGGTCCAGGTGACTCGTT	1.07	1.09	1.10
PPIA	CAAGACTGAGTGGTTGGATGG	TGTCCACAGTCAGCAATGGT	1.08	1.10	1.16
RPL13A	ATTGTGGCCAAGCAGGTACT	AATTGCCAGAAATGTTGATGC	1.16	1.13	1.13
SLA-1	GGGTCCCCACTCCCTAAG	ACGTAGCCGACTTCGATGA		1.15	
SLA-3	CCCTCGTTCTGCTGCTCT	CGGCGGTGTAGAAATAGCTC			
STAT1	CACGAAGGTGATGAACATGG	TCTTTCAGTTGCAGGTGTCG	1.10	1.03	1.09
TAP1	CCCCTTCCTGAATATGAGCA	CAGGCCATAGGCAATGTTTT	0.92	1.08	1.18
TAP2	GAGCATCACTACCTGCACCA	AGCTCTTCAGCCCATAAGCA	1.02	1.04	1.19
TLR7	GGAAATAGCATCAGCCAAGCTC	TTCCAGGTTGCGTAGCTCTT	1.08	1.10	1.08
TNFSF10	TACAGTTTCAGAAAAGCAACAAGG	GTGAGCAGCCACTCTCTGTG	1.05	1.04	1.06
YWHAZ	GCTGCTGGTGATGATAAGAAGG	AGTTAAGGGCCAGACCCAAT	1.15	1.09	1.10

Table S3. Relative expression of genes involved with virus recognition, immune response and inflammation in the obese-mock group compared to the lean mock group detected by qPCR in the lung. Data presented as group means ± SEM, with lean-mock group (n=4) scaled to 1 and the obese-mock group (n=4) shown as relative expression. *P* ≤ 0.05 in bold.

	Nasal mucosa			Trachea			Lung		
	lean-mock	obese-mock	*P-value*	lean-mock	obese-mock	*P-value*	lean-mock	obese-mock	*P-value*
CASP1	1.00 ± 0.32	1.48 ± 0.15	0.23	1.00 ± 0.42	0.81 ± 0.11	0.85	1.00 ± 0.40	0.24 ± 0.06	0.1
CD86	1.00 ± 0.18	1.53 ± 0.29	0.33	1.00 ± 0.41	0.94 ± 0.14	0.7	1.00 ± 0.41	0.51 ± 0.06	0.46
CTSS	1.00 ± 0.33	2.27 ± 0.37	0.17	1.00 ± 0.40	1.22 ± 0.29	0.46	1.00 ± 0.52	0.17 ± 0.05	0.28
CXCL10	1.00 ± 0.74	1.94 ± 0.48	0.15	1.00 ± 0.66	0.75 ± 0.20	0.6	1.00 ± 0.42	0.79 ± 0.12	0.93
CXCL8	1.00 ± 0.30	1.49 ± 0.51	0.54	1.00 ± 0.30	1.09 ± 0.22	0.64	1.00 ± 0.40	0.87 ± 0.34	0.9
CXCL9	1.00 ± 0.69	2.22 ± 0.23	0.14	1.00 ± 0.72	0.47 ± 0.09	0.82	1.00 ± 0.24	1.05 ± 0.29	0.93
GBP1	1.00 ± 0.34	1.38 ± 0.11	0.3	1.00 ± 0.50	0.94 ± 0.09	0.56	1.00 ± 0.16	2.19 ± 0.81	0.22
HSP70-2	1.00 ± 0.29	1.11 ± 0.45	0.98	1.00 ± 0.10	1.09 ± 0.09	0.57	1.00 ± 0.39	6.22 ± 1.24	**0.03**
HSPA6	1.00 ± 0.52	1.49 ± 0.71	0.51	1.00 ± 0.04	1.09 ± 0.12	0.68	1.00 ± 0.32	12.90 ± 5.62	0.2
IKBKE				1.00 ± 0.14	0.82 ± 0.08	0.46			
IRF7	1.00 ± 0.34	0.50 ± 0.12	0.36	1.00 ± 0.33	0.63 ± 0,.14	0.46	1.00 ± 0.14	0.74 ± 0.20	0.32
ISG12A				1.00 ± 0.51	0.26 ± 0.05	0.46	1.00 ± 0.81	0.86 ± 0.34	0.32
OAS2	1.00 ± 0.47	0.67 ± 0.11	0.87	1.00 ± 0.51	0.77 ± 0.51	0.88	1.00 ± 0.48	0.46 ± 0.12	0.47

SLA1				1.00 ± 0.15	1.81 ± 0.65	0.99			
STAT1	1.00 ± 0.23	1.13 ± 0.11	0.52	1.00 ± 0.31	0.81 ± 0.02	0.9	1.00 ± 0.31	1.54 ± 0.59	0.48
TAP1	1.00 ± 0.26	1.40 ± 0.29	0.39	1.00 ± 0.14	0.81 ± 0.22	0.42	1.00 ± 0.39	1.75 ± 0.31	0.21
TAP2	1.00 ± 0.25	1.39 ± 0.26	0.38	1.00 ± 0.25	0.82 ± 0.06	0.75	1.00 ± 0.33	1.69 ± 0.31	0.28
TLR7	1.00 ± 0.38	2.63 ± 0.48	0.1	1.00 ± 0.27	0.78 ± 0.06	0.69	1.00 ± 0.11	2.58 ± 1.24	0.29
TNFSF10	1.00 ± 0.37	1.53 ± 0.14	0.2	1.00 ± 0.29	0.75 ± 0.09	0.69	1.00 ± 0.36	0.91 ± 0.30	0.9

Table S4: List of proteins identified in the lung proteomics analysis between obese-mock and lean-mock, including protein name, UniProt accession, log2 fold change, and Benjamini–Hochberg adjusted p-value. Proteins with fold change ≥1.5 and adjusted p-value ≤0.05 are considered significantly differentially expressed between obese-mock and lean-mock. Adjusted P ≤ 0.05 in bold.

Gene name	UniProt accession	Fold Change (log2)	Adjusted P-value
APOE	F1RM45	-11.17	**0.00781546**
APOA4	O46409	-2.41	0.08689860
MYH10	F1SSA6	-0.58	0.58727123
CLASRP	A0A286ZYA8	7.52	0.87591338
TSTD1	A0A4X1VRY9	1.51	0.89739469
RAB21	A0A287A4W6	0.76	0.91611035
ALB	P08835	-0.72	0.91673254
THRAP3	A0A4X1VYM7	-2.69	0.91776473
CCDC43	F1RQZ4	2.94	0.91904610
LOC100624077	A0A287BEY3	-2.27	0.91917102
RASIP1	I3LIC8	0.51	0.91999698
PRKCB	K9J4L8	0.82	0.92032085
ADI1	I3LEI8	0.41	0.92040068
SEC14L3	A0A286ZQ63	0.92	0.92155096
ANXA5	F2Z5C1	0.46	0.92178566
SORD	A0A287AT21	-2.81	0.92234530
ACADL	P79274	0.60	0.92243971
SOWAHD	F1RUA2	1.48	0.92268958
CENPV	A0A5G2R844	1.00	0.92281379
RPL39	K7GP63	1.05	0.92297981
SERPINH1	A0A286ZRU9	-0.15	0.92345972
ANK1	F1SE30	0.59	0.92378194
	A0A075B7J0	-0.21	0.92392987
KRT85	F1SGI3	-3.31	0.92413898
GNA13	A0A287A853	1.99	0.92427338
PGLS	A0A287APK0	0.74	0.92430854
MYG1	F1SFR1	1.74	0.92433984
YWHAB	A0A8W4F6F6	0.60	0.92434213
SPATS2L	A0A287ANT4	2.64	0.92447739
HARBI1	F1SIA2	0.81	0.92450855
MRPS34	A0A287AVS9	0.50	0.92468619
U2AF2	A0A481APG3	-0.48	0.92488559
	A0A8D0STA6	3.04	0.92496826
ARHGEF17	F1SUT6	3.40	0.92512744
UQCRFS1	F1RNZ1	2.33	0.92520839
PFKP	F1RX49	0.71	0.92523764
SRSF2	Q06A98	0.50	0.92530912
GP9	B7TY22	-2.37	0.92533944
RPS28	Q6QAT1	-0.43	0.92547050
RETN	Q6QR67	0.43	0.92560135
DDRGK1	A0A287BPF3	2.55	0.92561705

RAB14	Q52NJ6	0.79	0.92573354
DENR	A0A4X1UG52	0.53	0.92584745
SYNPO	A0A286ZLL9	0.32	0.92588979
	A0A5G2QXT5	0.78	0.92591530
LAMC2	F1S662	0.55	0.92616107
CHMP4A	A0A4X1SRC3	1.96	0.92619366
UBE4A	A0A5G2QH63	-0.92	0.92623056
RCC2	F1SUQ8	0.75	0.92623969
HSPA8	F1S9Q1	-0.70	0.92630183
HNRNPH1	I3L816	0.55	0.92639940
	A0A287AEW5	2.22	0.92641152
ATP5F1E	K7GS22	0.82	0.92653025
CORO1B	F1RUX1	0.63	0.92675472
COX5A	F1SJ34	-0.56	0.92677671
PCNP	A0A8D1GJK2	0.59	0.92703674
GAA	F1RZ82	-2.43	0.92716557
ATP5PF	P13618	0.80	0.92724211
EFNB2	A0A4X1U8S9	0.92	0.92725368
ERAP1	K7GLU6	-0.74	0.92750541
UBQLN2	A0A286ZWA5	2.92	0.92758691
ARPC5	A0A8X9AEQ6	0.69	0.92777497
ARPIN	A0A287B5T1	0.58	0.92780305
TAGLN2	A0A4X1VV05	0.43	0.92788056
MYH9	A0A8W4FCU2	0.42	0.92794845
PTGS1	A0A287ANF9	-0.91	0.92795997
SOD3	I3LUD1	0.23	0.92797578
THY1	A0A287B2P9	-0.69	0.92803872
PIR	K7GKW6	-0.52	0.92808299
HBB	P02067	-0.44	0.92845592
PSMA7	A0A287AT18	0.54	0.92860437
LSM6	A0A8W4FJI1	1.16	0.92865245
BLVRB	I3LQH7	-0.47	0.92871312
DYNLT3	I3LUI9	1.10	0.92872540
GRPEL1	A0A287AD92	-0.68	0.92879372
NDUFB9	K7GSE5	2.72	0.92894319
WDR1	K9IVR7	0.63	0.92903692
VTN	P48819	-2.23	0.92904230
ATP6V1C1	A0A287AXI2	-0.57	0.92909224
STIP1	A0A8D1UVZ9	0.54	0.92914191
SLA-DRA1	Q85ZW4	1.02	0.92917667
RASA3	A0A287A546	0.59	0.92917766
APOA1	K7GM40	-0.80	0.92919522
ABHD14B	A0A287A5T1	0.34	0.92924551
DTD1	F1SBH1	0.97	0.92927825
IVD	A0A8D0UIS6	0.59	0.92934343
PSMA5	F2Z5K2	0.56	0.92942696

KANK2	A0A287B2K8		0.61	0.92956993
AHNAK	A0A287A608		0.57	0.92963283
COLGALT1	A0A287AXP9		-0.35	0.92963795
	A0A287ANX9		-2.70	0.92971129
LOC110258309	A0A8D1APX5		-2.18	0.92984339
AKR1A1	P50578		0.46	0.92991153
RPL18A	A0A287APR1		0.33	0.92996791
PPP2R5E	A0A4X1WDN4		0.79	0.93012441
PLCG2	F1S475		-2.24	0.93020892
LRPAP1	F1S8M9		0.49	0.93040425
LOC100525613	A0A8W4FHM5		0.49	0.93044806
S100A4	F1SFV7		2.38	0.93048981
COBLL1	A0A286ZK31		-2.26	0.93050063
ECHDC3	F1RUP0		2.52	0.93060232
CHUK	F1S8V5		-1.96	0.93067223
COL18A1	A0A286ZIL9		-0.91	0.93076564
RPL28	A0A287AJT7		-1.07	0.93076876
PUS1	A0A8D0NHD1		2.55	0.93082349
PLEKHF2	A0A287ASA9		0.84	0.93102545
LY6G6F	A0A1L6ZA08		-2.14	0.93106111
LOC110259265	A0A287BIA5		0.77	0.93117555
PSMD9	A0A286ZLP7		0.80	0.93123146
MATN1	I3L5Q7		-2.72	0.93134965
PPP2R1A	P54612		0.45	0.93136456
SHANK3	A0A287ASF0		0.80	0.93146256
VPS29	A0A5G2QIE0		-0.54	0.93156958
KRT35	F1S0K7		-2.98	0.93164863
TPR	F1S300		-0.52	0.93189501
HSPB6	A0A287AQR8		-0.52	0.93197871
CNPY2	A0A4X1W9B8		2.24	0.93205286
RPL37A	A0A4X1UMQ6		0.65	0.93208957
GNG2	A0A4X1WDD8		0.84	0.93213760
PTPN12	A0A287BKD5		1.28	0.93225034
DNAJA2	F1RP05		0.61	0.93234406
PHF5A	A0A286ZJW8		-0.51	0.93246236
CTSZ	A5GFX7		-0.70	0.93258789
TUBB1	A5GFX6		-0.65	0.93273563
VAT1	I3L9V2		-0.50	0.93305686
LOC100624149	A0A4X1T8G7		-3.05	0.93322659
VIM	P02543		0.59	0.93330805
FETUB	F1SFI6		1.21	0.93424522
NDUFA2	F1RGE3		1.99	0.93444828
EIF3A	F1S4P6		0.51	0.93473271
ISG15	A0A287AB58		0.66	0.93477160
HOPX	Q8MJD6		0.45	0.93525086
SRSF9	F1RJJ0		-2.19	0.93546496

LOC110258312	A0A287AFE3	-2.09	0.93626145
LOC106504545	F1SCC9	-0.41	0.93637638
LOC110259073	A0A286ZMJ2	2.54	0.93669845
RBBP4	A0A5K1VU58	0.59	0.93740855
PPL	F1RK90	0.47	0.93750598
BLOC1S3	A5A769	1.66	0.93761121
GIT1	F1RN87	0.95	0.93763236
RPS11	F2Z4Y8	0.38	0.93782332
CEBPB	E5Q8C6	1.06	0.93840754
EPX	F1RSB4	0.54	0.93866725
ACTN4	F1RI39	0.43	0.93874413
ACTR5	F1SDX1	-2.34	0.93889468
RPL27	A1XQU5	-0.52	0.93924650
PKM	A0A286ZI36	-0.32	0.93925197
HNRNPDL	A0A286ZYE7	0.61	0.93931197
RPS12	P46405	0.28	0.93946101
PDXK	A0A286ZIG4	0.60	0.93947775
CDK6	K7GT60	-2.54	0.93949388
AK4	B1NI70	-0.62	0.93953074
NUDT5	A0A480XE17	0.51	0.93982316
PLVAP	A0A287AU22	-0.60	0.93995201
TTLL3	B5APU9	0.22	0.94009073
LAP3	P28839	-0.71	0.94012813
PRDX6	Q9TSX9	0.37	0.94015041
EIF4B	A0A287AT83	-1.98	0.94041553
SLC25A24	F6Q4L6	0.54	0.94047797
SOD1	P04178	0.32	0.94052380
APMAP	A0A287A6K7	0.30	0.94068062
IFIT3	F1SCY2	0.75	0.94105933
NHLRC2	F1S5H7	-0.66	0.94113030
TKT	A8U4R4	0.53	0.94113427
COMMD5	A0A4X1UP09	-0.44	0.94114080
NDUFA8	F1SLR1	0.44	0.94115892
CAP1	F1SMC0	-0.39	0.94133307
FGB	P14477	0.66	0.94142039
TACSTD2	F1S7A4	1.71	0.94162266
LOC100738684	A0A286ZJU5	0.53	0.94162558
TPM2	A0A4X1VY40	0.42	0.94164216
KRT1	F1SGG3	0.67	0.94173402
CNRIP1	A0A5G2QNN7	-1.52	0.94198626
NUP62	A0A287AZ57	0.52	0.94199606
LOC100514282	A0A286ZJ89	-2.33	0.94219144
DNAJB4	A0A8D1UUV8	3.76	0.94239932
EMD	A0A0U2ETD0	0.34	0.94241257
KCTD12	A0A8W4FMQ4	0.38	0.94241956
RPL12	A0A4X1U700	-0.73	0.94243592

HSPB3	A0A4X1SMX6	-2.33	0.94264403
PSMD8	A0A287B5Q7	-0.28	0.94265320
CASKIN2	A0A287BC09	0.89	0.94265874
NSD2	A0A287A8W1	0.87	0.94270235
LRATD2	F1RRR7	2.37	0.94271992
DDT	A0A8W4FG18	0.71	0.94274660
PPIL1	A0A4X1STN7	0.97	0.94278884
C1QC	A0A286ZSJ7	-0.35	0.94278977
FMO3	I3LS94	0.56	0.94280225
GNAQ	Q2PKF4	0.57	0.94287606
LOC396685	F1SCD0	-0.38	0.94291315
H4	P62802	0.43	0.94306610
SNX12	F2Z596	0.57	0.94309366
PDCD5	F1RNX2	-2.04	0.94310507
GPD1L	A0A8D0R9P0	-0.36	0.94319266
RRAGC	A0A287ASH4	0.63	0.94324153
COX6A1	A0A8D1LI22	0.43	0.94327253
CAV1	A0A286ZW86	0.85	0.94336847
CDKN2AIP	F1RT10	0.81	0.94339116
CNPY4	A0A287BQG2	2.40	0.94349511
VPS35	A0A5G2R4Q7	1.27	0.94349907
BICD2	A0A287AJT8	2.10	0.94352961
CRKL	A0A4X1UYQ0	0.33	0.94353962
FKBP4	A0A5G2QVN7	0.67	0.94376168
RPS8	F2Z5F5	0.45	0.94381077
SRM	A0A480SJI2	-0.76	0.94393656
CBX1	F1RWH1	-3.85	0.94398407
PA2G4	I3LVF5	-0.51	0.94404453
NIT2	F1SKY2	1.92	0.94416677
AGER	K7GQN4	0.79	0.94418400
SNTB2	I3LDU9	0.37	0.94423811
SUMO2	A0A8D1JI91	0.78	0.94433422
MX1	P27594	-1.66	0.94434313
CPNE1	F1S4V8	-0.36	0.94438569
GPLD1	F1RUE4	-0.58	0.94440440
EIF3F	A0A287ARI1	0.36	0.94451198
PSMC1	F2Z5J1	-0.37	0.94458737
TRIP6	I3LCI1	1.51	0.94460830
FMOD	F1S6B5	0.46	0.94468616
RANGAP1	A0A5G2R6N4	0.63	0.94497634
MAPRE2	F1SAJ9	0.74	0.94499668
GMPR	F1RUJ4	-1.96	0.94501169
COL4A3	F1SNP0	0.45	0.94504665
PPP1R3D	F1RJ12	-2.38	0.94511406
UBE2V2	I3L6T2	0.72	0.94511613
CYBA	A0A287BG06	2.78	0.94536009

ASPN	F1SUE4	0.46	0.94553050
SGCD	A0A5K1VFF8	-3.90	0.94553961
KRT82	F1SGI8	-3.32	0.94554844
RPL13A	A0A8D0MLF8	0.65	0.94565801
ACTBL2	A0A287A4R1	-0.26	0.94578756
CHCHD2	F1RIU9	0.80	0.94591216
RPSA	Q4GWZ2	-0.46	0.94601859
ABRACL	F1S6Z3	-0.64	0.94636616
EIF3C	A0A287AVR0	0.46	0.94637223
RNASE4	P15468	1.17	0.94663493
WDR82	F2Z5U3	2.16	0.94677535
CLEC3B	F1SRC8	0.47	0.94711897
SETX	A0A480HN89	1.93	0.94716851
RRAS	F1RHW4	2.93	0.94751306
ALDH3B1	I6L6E1	3.35	0.94769411
INMT	A0A287B0Z8	0.43	0.94772637
LRRC47	A0A287AYY7	-0.34	0.94774931
APOC2	F6PSJ3	-3.03	0.94783805
PPP1R9B	F1RT57	1.06	0.94814216
SBDS	F1RJJ3	0.51	0.94855011
SNRPF	A0A4X1SJX5	1.25	0.94876038
RBM17	F1RUL1	-0.57	0.94884433
IRF2BPL	A0A8W4FEM4	0.89	0.94886725
LOC100621639	I3LK01	-3.21	0.94923739
	A0A8W4FPQ3	0.51	0.94948776
H3-3A	Q71LE2	0.68	0.94995101
MACF1	A0A287AEG1	1.06	0.95000284
SCRN3	A0A8D1VMH1	3.13	0.95001385
EIF5	A0A287A7Q7	1.20	0.95010567
LYZ3	P12069	-4.51	0.95057533
TGM2	A0A8D1Z1H8	-0.53	0.95077209
FKBP3	I3LR51	0.83	0.95088862
ApoN	Q68RU1	0.48	0.95125333
VPS28	A0A4X1UQN2	-0.56	0.95148972
NDUFA5	A0A287BAW0	-3.88	0.95180562
PRKAR2A	P05207	-0.29	0.95313919
SSRP1	A0A286ZSH4	3.59	0.95345268
HDHD2	F1RZX9	0.66	0.95352947
DCLK1	A0A286ZLR2	1.02	0.95368252
FGG	I3LJW2	-0.29	0.95371052
CAV2	Q2QLE2	0.73	0.95436502
COL5A1	F1S021	2.79	0.95441512
RABGAP1	I3LBD0	-1.80	0.95447910
ILF2	K7GS49	2.91	0.95494521
DHX9	K7GND3	0.53	0.95509630
PTGDS	Q29095	2.26	0.95518429

LSM2	A5A8V8	0.70	0.95522381
NEK9	F1S2Q4	0.40	0.95531848
MGAM	A0A287A042	1.33	0.95563459
RNF114	Q6J1I8	-0.25	0.95598885
SEPTIN1	F1RG60	-1.41	0.95604477
TXNDC12	A0A481A893	-0.70	0.95613410
COL6A1	I3LS72	0.38	0.95630822
GNB3	F1SLU8	-0.54	0.95635943
AGPS	A0A287BCV1	0.54	0.95670861
QARS1	M3TYW5	-0.75	0.95684062
PRX	A0A287AUV6	0.31	0.95685962
ACTR3	F2Z5D2	0.54	0.95726779
SRRT	I3LI05	0.70	0.95766942
RPL11	Q29205	-0.67	0.95768100
EPDR1	A0A4X1UPP6	0.64	0.95785751
DES	P02540	-0.54	0.95796977
RHOB	Q06AT8	0.38	0.95806811
STX11	F1S731	0.26	0.95846498
	A0A8W4FGT8	-1.70	0.95861958
LOC100736951	F1SNU4	2.59	0.95870654
LAMA5	A0A8W4FFH5	0.38	0.95870893
GNPDA1	A0A287AHC2	0.28	0.95901229
KIF5B	F1RWC5	0.53	0.95955470
KRT34	F1S0K4	1.61	0.95959534
TAGLN3	A0A286ZKF7	-1.59	0.95962020
RPS27A	A0A287AZA7	0.46	0.95975736
LA-DRB-d	Q31072	0.43	0.95983471
	A0A8W4FEZ7	0.41	0.95983545
NID1	F1RGY5	-0.54	0.95993921
PACS1	A0A287BMC4	1.28	0.95996988
ADCY3	F1SDL2	0.52	0.96006331
CHMP1A	I3LU89	-0.48	0.96010195
BLMH	F1RN71	0.78	0.96012028
PDPR	A0A287AAG7	0.69	0.96020765
ARPC1A	A0A287BN59	0.62	0.96038395
PFN2	A0A5G2QQU9	0.47	0.96046124
THOC7	F1SGF4	0.26	0.96052759
HSD17B10	K7GP28	-0.67	0.96056831
CHMP6	F1RZY6	3.01	0.96067292
RAB1B	Q06AU7	-0.33	0.96082821
STAT2	O02799	0.40	0.96085690
CLPTM1L	A0A287B9L7	0.24	0.96086690
FBLN1	A0A286ZLN2	-0.49	0.96089050
EHD2	I3LD72	-0.27	0.96095229
UFL1	F1RY04	0.38	0.96096329
RPS9	A0A4X1VNN9	-0.41	0.96107579

SEPHS1	A0A286ZVF7	0.98	0.96113618
PRKAR2A	A0A287AEK2	-0.34	0.96119111
SNRPD1	A0A4X1VMY1	1.14	0.96119330
PSMD7	A0A287AEH0	0.79	0.96162900
EIF3D	A0A4X1U4W6	-0.37	0.96176546
	A0A8W4FHY5	2.58	0.96184876
DNAJA1	F1SE73	0.85	0.96281261
MITD1	F1STE3	-2.11	0.96285212
ATOX1	D0G0C7	0.35	0.96296305
FMO1	P16549	0.64	0.96303913
CFL2	Q5G6V9	-0.21	0.96305350
ARPC2	F1SS01	-0.28	0.96321878
MMP9	A0A287AZD5	0.73	0.96327295
INF2	A0A286ZUJ0	-0.65	0.96341729
PIP4K2A	F1RVI6	-0.22	0.96372997
GARS1	A0A8W4F705	-0.25	0.96373074
HCFC1	A0A8W4FGM9	0.42	0.96383327
HDLBP	A0A8W4FKC7	0.46	0.96385763
	A0A8W4FPY6	0.21	0.96439972
PGAM1	A0A287AJQ2	0.28	0.96464419
MYCBP	F2Z5W0	3.64	0.96468082
BANF1	F1RU33	0.32	0.96470323
GNB2	F2Z4Z8	-0.26	0.96486607
FRG1	F1SES4	1.02	0.96488518
SF3B1	A0A287AV35	0.36	0.96501141
ATP6V1F	A0A8D1H0W9	-0.25	0.96505033
SRSF5	A0A286ZX74	0.25	0.96517670
PR39	P80054	0.54	0.96538228
PRKAR2B	A0A286ZNQ7	-0.28	0.96551935
PLG	P06867	-0.47	0.96558315
MCRIP1	A0A287AWN1	1.07	0.96587773
RPL34	Q29223	-0.14	0.96632650
RPL9	I3LP78	-1.55	0.96658437
HSPA5	A0A287BIL8	-0.31	0.96661639
DAP	A0A286ZQE9	-0.49	0.96664616
CCDC197	A0A287A4J4	-0.29	0.96680830
UQCRB	A0A286ZPR8	-0.34	0.96682415
RPL30	A0A4X1TXJ8	-0.40	0.96692718
CCDC124	F1S924	0.21	0.96692857
	A0A075B7H9	0.16	0.96714208
NCK2	B6E314	0.35	0.96719829
TNFRSF6	Q56VC2	-0.38	0.96721516
CBR2	A0A287BT95	-1.12	0.96736989
RBM14	A0A287B401	0.29	0.96751826
S100A12	P80310	0.28	0.96757152
APOC3	P27917	-2.51	0.96790114

PCBP1	A0A8W4FAT6	-0.33	0.96791789
CDC42	Q007T2	0.24	0.96794053
RPS15A	A0A287A014	0.29	0.96800568
RANBP1	A0A287BA60	-0.48	0.96808163
IPO5	A0A5G2RIC0	-0.57	0.96813011
TIMM13	A0A8W4FNS1	0.43	0.96822371
PEBP1	A0A4X1TTH9	0.72	0.96833894
H1-0	A0A287BQW3	0.34	0.96845354
OTUB1	C5H0C6	0.28	0.96847166
UPF1	A0A286ZYJ8	0.58	0.96852341
SSBP1	A0A287A6V6	1.25	0.96945754
KRT2	A0A287BHY5	-0.22	0.96961767
LMAN1	F1SMS8	0.35	0.96981578
RPLP2	F1RYZ0	1.32	0.96985705
LMOD1	A0A286ZWL1	0.30	0.97047363
	F1S9H7	-1.21	0.97090337
ACAA2	D0G0B3	0.22	0.97097207
KRT5	F1SGG6	0.27	0.97108748
LMNB1	F1RKM0	0.32	0.97112463
S100A8	C3S7K5	0.96	0.97121082
QSOX1	F1S682	0.33	0.97123700
FAM98A	F1S408	1.99	0.97140696
C5H12orf57	A0A287A018	0.54	0.97146313
CRIP2	A0A5G2QBE4	0.23	0.97162462
PSTPIP2	A0A4X1UTD4	1.27	0.97169630
NOLC1	F1S8T1	0.22	0.97170080
PPBP	P43030	-3.40	0.97189829
HNRNPK	A0A287B646	0.43	0.97192803
GMPR2	F1SGN8	1.29	0.97217099
TARDBP	A0A8D0UXS6	2.91	0.97235290
DCTN1	A0A286ZRC7	0.39	0.97245954
CEL	I3LTK9	0.10	0.97254401
HNRNPH2	A0A287A9H4	-3.45	0.97268204
TIMM9	F1SSK4	0.89	0.97311659
PGP	A0A287BDT8	0.65	0.97332765
AP1M2	I3LE99	8.22	0.97344819
CROCC2	A0A287A2P1	5.13	0.97365882
P3H1	A0A287BT04	3.24	0.97367881
	A0A8W4FCL7	-0.55	0.97439565
RPS17	Q6QAP7	0.59	0.97452716
	A0A8W4FJA1	0.58	0.97481580
FSCN1	C0LZL0	0.57	0.97485610
LOC100519675	A0A4X1TPT1	-0.67	0.97487502
POSTN	I3LDM1	3.29	0.97495779
SLC4A1	K7GR72	-0.21	0.97500564
A2M	A0A5G2Q8I9	-0.13	0.97544606

	A0A8W4FMA6	-1.35	0.97557345
NUDC	A0A287B8R2	0.70	0.97585554
PCOLCE	I3LEE6	1.00	0.97587912
IGHM	A0A287ALC1	-0.43	0.97650314
LTBP4	A0A287BGV1	-0.23	0.97657887
	I3LFU3	1.07	0.97728225
ARCN1	A0A5G2QF14	0.96	0.97730191
SPAG8	A0A8W4FDW8	0.20	0.97731640
SMARCC1	A0A8D0WZ72	3.14	0.97744996
ALDOA	A0A286ZYX8	0.37	0.97748489
SFTPA1	F1SER3	0.63	0.97756841
NDUFA4	I3LJI1	0.92	0.97778503
SAP18	A0A480ULF1	-0.24	0.97807069
FGF2	A0A287BGK8	0.70	0.97855953
RPS27L	A0A4X1TYR3	0.92	0.97860857
MIEN1	A0A4X1UGL0	0.28	0.97871593
PRPF8	F1RHH8	0.50	0.97874196
ZFPL1	F1RQT4	-4.56	0.97883924
ANKRD44	A0A287B787	0.27	0.97897937
PITHD1	I3L9H4	-1.47	0.97937937
ITCH	A0A8D1VEE4	-0.23	0.97978646
FTL	P19133	-3.12	0.97979738
LANCL1	A0A8D1RD34	0.65	0.97982174
RPL6	Q2YGT9	0.48	0.97991508
SELENOF	A1Z623	-0.46	0.98017140
GATD1	F1RYY7	0.84	0.98048588
CD248	K7GPF5	-0.71	0.98061762
APOA2	A0A481BCM9	-1.11	0.98069817
CLINT1	A0A287BKR6	0.85	0.98074943
COL6A3	A0A287BPF4	0.80	0.98094510
VAC14	F1S3E8	-0.87	0.98122590
PRELP	F1S6B4	0.04	0.98145054
MYG1	A0A287A0Y1	3.42	0.98162849
POGLUT2	F1RLQ5	-6.97	0.98171991
SERPINC1	A0A5G2QUE0	-0.23	0.98188273
SAR1A	Q52NJ3	-0.32	0.98200670
DARS1	A0A4X1SYV7	0.32	0.98201129
G6PD	A0A8W4FNL6	0.61	0.98203958
	A0A8W4FE04	-0.22	0.98204967
COMMD3	A0A4X1TSS3	0.44	0.98220930
EZR	F1SB42	0.75	0.98238988
ARPC3	B5APU8	-2.55	0.98250520
H2AC21	A0A4X1SJ31	0.21	0.98279140
GSN	A0A286ZIC1	0.68	0.98286941
NDUFS5	F1SV23	0.28	0.98329179
PGLYRP1	A0A286ZI97	0.47	0.98334424

PDLIM2	A0A8D1S2M8	0.32	0.98336092
	A0A5G2QBU3	-3.69	0.98340106
TMEM74	K7GT14	-0.22	0.98347852
LAMB2	A0A287AWV5	0.80	0.98348458
EEF2	A0A4X1VS57	0.60	0.98350063
PSMD6	F1SGF1	0.58	0.98369696
MYDGF	M3UZ63	-0.17	0.98392902
TLN1	A0A287BAK1	-0.18	0.98407506
MIF	P80928	0.21	0.98409526
NDUFB10	I3LDC3	-0.12	0.98415725
UBE2Z	I3LSU8	0.17	0.98417688
LXN	I3LN84	0.23	0.98432301
CRYAB	Q7M2W6	0.19	0.98432620
RPS14	A0A287BHE7	0.07	0.98440002
RPL18	Q95342	-0.23	0.98443230
COX5B	Q5S3G4	0.15	0.98453864
PPP1R12A	A0A5G2QU87	3.95	0.98470113
NUDCD2	A0A8D1TE97	0.87	0.98492814
UAP1L1	A0A287AAG0	0.57	0.98495376
EIF3J	F1SN05	-1.63	0.98510974
NEK7	A0A4X1SPG3	-1.03	0.98538220
UMPS	A0A5G2RBV3	-0.19	0.98541734
RPN1	A0A480VU46	0.16	0.98551652
LGALS13	B7U2G5	1.29	0.98551982
RPS4X	A0A287B771	-0.16	0.98555436
LOC100525573	I3LJT0	0.56	0.98570189
RPL36	A0A8W4FBD1	0.71	0.98574593
FERMT3	F1RQ01	-0.19	0.98588350
ARPC5L	A0A5K1VDW0	0.65	0.98590673
WDR26	F1S8S6	-1.07	0.98591642
THBD	A0A8W4FEQ7	-0.81	0.98595442
SSB	F1S1V1	-0.20	0.98615656
PUS7	F1SB38	0.32	0.98635577
CAVIN1	A0A286ZZ97	0.85	0.98654871
PFDN2	F1S192	0.24	0.98657107
RAB24	F1S3C2	0.52	0.98659557
LOC110255312	A0A287AEL2	0.83	0.98667511
NELFB	A0A287BM68	2.30	0.98671548
CCAR2	F1RMA3	0.13	0.98676601
PAFAH1B3	F1RGI2	-0.18	0.98682152
TPP1	I3L812	0.34	0.98683249
RPS18	P62272	-0.22	0.98687402
KCNE2	A0A4X1TSJ2	-0.15	0.98705615
PALLD	F1RIW3	0.47	0.98725484
PARK7	Q0R678	0.23	0.98739542
MC2R	Q8HYN8	-3.34	0.98741331

AZU1	P80015	0.25	0.98751954
NEDD8	A0A287AE63	0.37	0.98756944
PSMC2	A0A4X1VCL1	0.10	0.98759102
COTL1	A0A287AJU1	0.16	0.98764460
AAMDC	A0A4X1UBD1	-0.31	0.98765938
GAPDH	P00355	0.21	0.98767719
H1-3	A0A5G2QML3	0.07	0.98768418
TMSB15A	I3LAC8	-1.10	0.98778840
SUSD2	A0A8W4FGK0	0.14	0.98786467
DUSP28	I3L5M7	3.24	0.98790846
NIBAN1	K7GRN9	0.67	0.98802947
POGLUT3	F1SV18	-0.30	0.98805000
SMARCA5	A0A4X1VHH1	0.23	0.98811773
CLIC3	A0A287A988	0.21	0.98811926
BCAP31	F1S2A8	-0.12	0.98822579
BCAS2	I6L631	0.25	0.98826626
RCN1	F1SGP8	-0.08	0.98844449
TXNDC9	F1STE6	0.24	0.98851230
RAB5C	A0A4X1U6G7	-0.17	0.98853723
COL6A2	I3LQ84	0.16	0.98855282
PABPN1	A0A287ASR3	-1.06	0.98857773
BAG3	F1S415	0.06	0.98867278
	A0A8W4FKJ9	-0.78	0.98869139
VARS1	A0A287BPW4	0.17	0.98873614
ANXA11	F1S2E2	0.16	0.98883818
RPL23	P62831	0.05	0.98884554
CAVIN3	F1RMM0	-0.13	0.98898369
KRT10	I3LDS3	0.20	0.98913197
SRGN	I3LKI1	-1.07	0.98920269
PFDN1	A0A287BED1	-0.42	0.98933999
PLCD1	A0A8W4FCN0	1.03	0.98934705
STRAP	A0A4X1VAU7	-0.18	0.98943551
ABCB10	F1RG41	0.29	0.98945388
PRDX4	F1SQ01	0.24	0.98953461
ADIRF	A0A287ACN2	0.73	0.98955133
TIMM10	A0A4X1VQG5	0.12	0.98957561
TFAM	Q5D144	0.35	0.98996725
H1-2	A0A8W4FCQ5	0.16	0.99013564
CACYBP	F1S710	-1.04	0.99055164
MAPK14	A0A5G2R9K2	0.23	0.99060105
PODN	F1S762	-0.17	0.99063616
KDM3B	F1RH75	0.63	0.99078302
LCP2	F1RRX4	0.31	0.99082345
SIGLEC1	A7LCJ3	-0.10	0.99095669
RUFY1	A0A5G2QEU1	0.48	0.99100661
HOXA5	F6Q7H3	0.32	0.99106865

UBE2V1	A0A8D0V9S7	-0.17	0.99108415
	A0A287ARG2	1.11	0.99117186
SERPINB10	F1SMW8	0.85	0.99124870
VPS25	A0A287AAM6	-0.19	0.99125996
TNS2	F1SGG0	1.04	0.99127490
NIBAN2	A0A287BEJ3	0.09	0.99133819
APPL1	B5LX40	-0.32	0.99140428
C7	F1SMJ1	0.18	0.99144666
ATP2B3	A0A5K1VCX5	-5.31	0.99148846
POLR2A	I3LJR4	0.20	0.99170130
RRBP1	I3LGC2	0.84	0.99175055
CERT1	I3LGX5	0.21	0.99177392
TMPRSS11D	A0A8D1FUI0	-0.27	0.99195488
DSG3	I3LU54	0.82	0.99210541
MAVS	A0A5K1V3V4	1.04	0.99211007
	A0A8W4FNF1	-0.12	0.99212465
DDX17	A0A287BEA5	-0.84	0.99214571
FN1	A0A286ZY95	0.11	0.99215910
BGN	A0A5K1UKC5	0.20	0.99225039
RPL13	I3LSD3	0.16	0.99232027
PURB	A0A287A4H2	-0.21	0.99240252
RPS25	A0A4X1SLJ1	0.29	0.99241471
PPP1R12A	A0A5G2R8E1	0.24	0.99258717
NDUFA13	A0A4X1U1Y7	-0.62	0.99259264
PDIA3	F6QA08	-0.09	0.99265871
YWHAG	F2Z4Z1	-0.13	0.99271562
DOCK1	A0A287A2N5	0.05	0.99283937
LOC100739611	A0A8X9AEY3	0.05	0.99296363
FN3KRP	F1RZZ3	-0.03	0.99300377
KPNB1	F1RWJ5	0.03	0.99308446
CFP	A0A5G2QHH9	-0.03	0.99323346
LMNA	Q3ZD69	1.10	0.99323473
TES	Q2QLE3	-0.03	0.99330317
TRMT112	F1RQP1	0.30	0.99339255
PRKG1	Q3YJM5	0.94	0.99341688
AVIL	I3LI80	-0.74	0.99342086
ATP1B1	P05027	0.75	0.99344629
PELO	F2Z5N9	-0.46	0.99359136
NOSTRIN	A0A5G2QEY8	0.75	0.99366104
LSM3	F2Z5S4	0.04	0.99372099
SRSF3	A0A4X1SUA0	0.10	0.99385637
RPL23A	A0A286ZL51	0.02	0.99397759
NAPB	A0A5G2QLD1	0.02	0.99399762
SSH3	A0A287B639	0.81	0.99399839
NDRG2	A0A287ACT9	3.95	0.99404067
MCCC2	A0A5G2QSH7	-0.03	0.99409984

MRC2	F1RRU7	-0.22	0.99416376
HNRNPM	A0A287AR13	-0.13	0.99420840
YWHAE	A0A287A2R9	-0.01	0.99429428
CLTB	A0A287BG47	-0.93	0.99437465
RAB23	A0A286ZTT8	0.12	0.99477169
SH3BGRL	F1S1R4	0.14	0.99492762
NHERF2	B6EAV5	0.91	0.99496080
	A0A5G2QZ41	1.32	0.99497186
H2AZ1	A0A8X9AEL2	2.63	0.99498944
RPL11	A0A5G2QRK2	0.05	0.99503333
UBAC1	A0A5G2QSM5	0.03	0.99503545
ACLY	A0A8D0NPF0	0.71	0.99507186
NFKB1	A0A287AVR3	-0.17	0.99517179
RPL38	A0A287AE36	0.57	0.99527102
SUMF2	F1RIU4	-0.30	0.99540771
LCP1	A0A287ANV7	0.14	0.99544337
NCF1	F1RJL1	1.43	0.99549160
LOC106504547	A0A287AGW0	0.74	0.99552219
RPL21	P49666	-0.04	0.99578402
IFIT5	A0A8D0WX68	0.04	0.99583765
CA1	A0A287AI92	0.16	0.99587276
PFN2	I3L660	0.14	0.99594961
TRIM25	A0A5G2QSW4	0.86	0.99613581
RBM8A	I3LI59	0.07	0.99614430
C4BPA	F1S0J1	-3.66	0.99621435
IGBP1	K7GQ35	0.03	0.99633463
ZNF22	A0A287B2Z6	3.69	0.99637471
CYB5R3	P83686	0.44	0.99661824
CAPN5	F1SUK6	0.04	0.99671793
PSMC4	A0A4X1SRU3	-0.04	0.99674921
HSPD1	A0A8W4FMW6	0.97	0.99679622
KANK3	F1SA52	0.73	0.99682954
UBE2M	A0A287BNE4	0.02	0.99688764
COX4I2	A0A287A440	-0.10	0.99692009
UBE2L3	B8Y648	-0.04	0.99703231
PTMS	A0A287B883	-0.02	0.99707671
NUP35	A0A480QBL1	0.12	0.99716151
HBB	A0A5G2QRW3	-0.09	0.99719941
HIP1R	F1REX8	0.50	0.99735804
ATP6V0D1	A0A5G2QDQ1	-0.01	0.99766792
NUP88	A0A287A9U8	0.02	0.99772389
RPS16	Q29201	0.04	0.99774480
BAG2	A0A287AV76	-0.01	0.99782380
SULT1C4	I3LC84	-0.02	0.99791531
RIDA	A0A286ZNH3	-0.01	0.99807810
WAS	A0A286ZTG3	1.00	0.99818530

DPY30	F2Z5F7	-0.13	0.99818607
TBC1D10B	F1RG61	4.17	0.99833385
	A0A287AMU3	1.76	0.99837239
EFHD2	F1SUW3	0.04	0.99852049
SGSH	F1RZ89	1.62	0.99856217
RPRD1A	A0A287B672	0.16	0.99857867
IQGAP1	A0A8D1IV93	-0.01	0.99862606
HSPG2	A0A286ZHV7	-0.08	0.99877326
H1-10	A0A8W4FQL0	0.86	0.99887127
DDAH2	A0A287A5S7	-0.13	0.99896447
NES	I3LNY6	0.04	0.99896798
CSTB	Q29290	0.15	0.99902734
SUPT16H	F1S8K5	0.01	0.99904695
EHD4	I3LDA5	-0.07	0.99907007
	I3LN83	-0.08	0.99912585
LIMA1	B0KYV5	0.15	0.99913583
XIAP	A0A8D0XQ50	0.06	0.99925636
LOC100737030	A0A287AHM5	-0.01	0.99930938
COPB1	F1S982	0.00	0.99937276
LOC100512873	I3LS74	1.27	0.99943618
EIF4EBP2	A0A286ZLA1	-0.01	0.99946517
DYNLL1	D9U8D1	-0.09	0.99948828
OLFML3	H6UWK6	0.90	0.99957409
METTL3	F1S8J8	1.07	0.99958592
WASHC4	F1SG40	0.00	0.99960987
RPS3A	A0A4X1SK65	0.05	0.99962233
ETHE1	F1RMV5	-0.01	0.99970679
ANXA4	P08132	0.20	0.99977784
TF	P09571	0.05	0.99978853
APRT	F1S6K5	0.08	0.99990737
RSU1	F1RW98	-0.01	1.00000000
HLA-DQA	P15980	3.67	1.00000000
GNG12	B9TRW9	0.00	1.00000000
IL1RN	Q29056	0.12	1.00000000
THBS1	F1SS26	-0.06	1.00000000
ESD	Q9GJT2	1.10	1.00000000
HCK	A0A287BSA9	0.25	1.00000000
PAG1	F1RXA1	-0.15	1.00000000
GSTO1	Q9N1F5	-0.62	1.00000000
GLRX	P12309	0.31	1.00000000
LOC100739080	F1SGS2	1.24	1.00000000
GGH	A0A286ZMB2	-3.76	1.00000000
KRT18	A0A5G2RBD3	-0.01	1.00000000
SUB1	A0A8D0WDE3	0.71	1.00000000
RETSAT	F1SVB2	-0.04	1.00000000
GYPC	A0A8D0WGG1	4.32	1.00000000

CLIP2	A0A286ZX08	0.71	1.00000000
MVP	I3LQ79	0.05	1.00000000
APOB	A0A287AG13	-7.36	1.00000000
GLO1	A0A8D0PDF1	-0.04	1.00000000
PTPN23	A0A287AFP0	1.86	1.00000000
UBA1	A0A5K1U589	0.74	1.00000000
HSPD1	A0A4X1VDY5	0.03	1.00000000
AK3	A0A4X1W814	0.04	1.00000000
EVPL	F1RW07	0.79	1.00000000
ACO2	P16276	0.07	1.00000000
CKAP4	F1SPP8	0.34	1.00000000
CLIC4	A0A287ADH9	-0.76	1.00000000
EIF2S2	A0A4X1T4S6	1.14	1.00000000
AHNAK	A0A286ZP76	-0.31	1.00000000
C2H11orf68	F1RU27	0.04	1.00000000
SFTPB	A7MAK5	1.21	1.00000000
PFDN6	F1RZT2	0.77	1.00000000
CDC40	F1RSY9	-0.31	1.00000000
COG2	F1RG46	5.55	1.00000000
OAS1	Q29599	0.37	1.00000000
HSD17B2	A0A287BB42	0.75	1.00000000
FIS1	A0A4X1UIB2	-0.16	1.00000000
CASP7	K9J6J4	-4.84	1.00000000
AHCY	Q710C4	-0.06	1.00000000
PRTN3	A0A8D0VTQ5	0.08	1.00000000
LOC100155195	A0A287B571	0.19	1.00000000
CDH11	F1RFU7	3.15	1.00000000
PDXK	O46560	0.11	1.00000000
SPTB	A0A4X1UE92	0.59	1.00000000
SMU1	A0A4X1UMT8	1.26	1.00000000
PDE12	F1SGK9	4.39	1.00000000
AP2A1	F1RH60	-0.97	1.00000000
	A0A287ATT2	0.41	1.00000000
ETF1	F2Z505	-0.06	1.00000000
LSM8	A0A4X1W3A0	-0.06	1.00000000
SEC62	I3LND2	-0.13	1.00000000
OSTF1	Q8MJ49	0.08	1.00000000
CBR3	F1SGX4	0.16	1.00000000
MOGS	K7N7E7	0.44	1.00000000
GZMB	A0A288CG12	-4.55	1.00000000
ARPC1B	B5APU6	-0.09	1.00000000
ING1	A0A287BKR1	-0.08	1.00000000
FABP4	O97788	-0.06	1.00000000
RPL4	F1SJJ5	-0.06	1.00000000
CGGBP1	A0A287ARL7	-0.29	1.00000000
HNMT	A0A287AA45	0.19	1.00000000

DCPS	Q8MIZ3	-0.38	1.00000000
ATP5IF1	Q29307	0.12	1.00000000
AKR1B1	P80276	-0.16	1.00000000
PGRMC2	A0A8W4FA00	-0.09	1.00000000
CLIC2	A0A4X1T8K6	-0.05	1.00000000
TSFM	A0A5G2QFS9	-0.64	1.00000000
VDAC1	Q9MZ16	-0.19	1.00000000
LOC110255331	A0A286ZVQ0	-0.09	1.00000000
CMPK2	A0A5G2RG89	0.41	1.00000000
PDLIM1	F1SC51	1.21	1.00000000
NCCRP1	I3LR13	-0.10	1.00000000
YWHAH	F2Z4Y1	0.04	1.00000000
NDUFA12	F1SQP4	-0.11	1.00000000
PLXNB2	A0A8W4FCM1	3.07	1.00000000
RPL32	Q6QAT0	0.07	1.00000000
IYD	A0A287BQ51	0.52	1.00000000
PSMB4	A0A287B088	0.11	1.00000000
DYNC1H1	A0A8W4FNQ7	-0.12	1.00000000
ABI3BP	A0A5G2QUX0	3.54	1.00000000
CNN1	Q08092	0.05	1.00000000
PSMD11	F1RKW8	0.32	1.00000000
MESD	F1RIE0	0.78	1.00000000
NAPRT1	M3TYR7	5.29	1.00000000
EVL	A0A480KZG7	0.08	1.00000000
IGF2BP2	K7GKD3	0.31	1.00000000
HNRNPM	A0A287BL93	0.03	1.00000000
GBP1	A0A287BSF6	-3.72	1.00000000
TMSB10	P21753	0.56	1.00000000
PRDX5	A0A4X1V8R7	0.09	1.00000000
CFD	P51779	-0.11	1.00000000
BMP2K	I3LT15	0.14	1.00000000
MRPL12	A0A4X1U6S6	0.74	1.00000000
APCS	O19063	3.22	1.00000000
LSM12	A0A8W4FFA0	3.15	1.00000000
CAT	A0A4X1SSR6	0.25	1.00000000
ITIH1	A0A287B3S3	-0.06	1.00000000
PHPT1	P59083	0.06	1.00000000
CNBP	A0A8D0UBE2	1.12	1.00000000
CLTA	A0A287BFJ2	0.07	1.00000000
DDAH1	A0A287BBK0	0.78	1.00000000
BIN2	I3L7Y9	-3.66	1.00000000
PTGR3	A0A287A6F5	-0.04	1.00000000
LMAN2	F1S3D5	-0.54	1.00000000
MYO1C	A0A286ZL61	1.07	1.00000000
GPI	P08059	0.68	1.00000000
UBA2	F1RNU6	-0.16	1.00000000

LOC110255360	F1RMQ6	-0.08	1.00000000
LOC396684	F1SCC7	-0.03	1.00000000
ARHGDIB	A0A4X1V4G4	-0.09	1.00000000
BUB3	A0A4X1SFI9	0.99	1.00000000
CS	P00889	0.08	1.00000000
ITGA1	A0A5G2R836	-0.12	1.00000000
DPYSL3	F1RLZ7	1.18	1.00000000
CPI17	O18734	-0.14	1.00000000
TMED10	A0A286ZV95	-0.02	1.00000000
TXNDC5	A0A287ARZ1	-0.08	1.00000000
FTH1	A0A287AL10	-0.13	1.00000000
CRIP2	A0A287A2M4	0.06	1.00000000
ITGA3	F1RT62	0.06	1.00000000
CSPG4	F1SJ86	1.05	1.00000000
KRT7	A0A287AG48	0.88	1.00000000
RNF20	A0A4X1VEK1	0.41	1.00000000
PSMD14	F1RPQ3	0.83	1.00000000
CREB1	A0A8D1W8I5	-0.18	1.00000000
LOC100522887	F1SV36	1.18	1.00000000
PODXL	F1SNF3	0.09	1.00000000
TPPP	A0A286ZLY5	1.03	1.00000000
RHOA	I3LVS7	-0.13	1.00000000
CRELD2	F1RXR2	-3.61	1.00000000
GOT1	P00503	0.91	1.00000000
EEF1AKMT1	A0A287BEE7	5.10	1.00000000
RDH14	F1SCT9	-4.63	1.00000000
ABCA1	F1SP55	-2.63	1.00000000
SERPIND1	A0A481BLI9	-1.50	1.00000000
DIAPH1	A0A5G2QRI9	0.75	1.00000000
CST3	Q0Z8R0	1.13	1.00000000
PPM1F	F1RL04	0.58	1.00000000
GC	A0A287AHK1	-6.02	1.00000000
	A0A5G2R996	0.81	1.00000000
PPID	F1RTY6	0.78	1.00000000
EPPK1	A0A8W4FFH6	0.80	1.00000000
HTRA4	I3L7K4	-2.98	1.00000000
EXOC3	A0A480TWJ1	1.15	1.00000000
CHMP4B	A0A4X1T3F8	0.85	1.00000000
gag	A8NBY5	0.59	1.00000000
BLOC1S1	A0A4X1W434	1.66	1.00000000
CCT8	I3LCA2	0.90	1.00000000
RPS19	F1RGG1	3.24	1.00000000
POLR2B	I3LGP4	1.27	1.00000000
PFKL	A0A480K104	1.49	1.00000000
	A0A287B5T7	0.45	1.00000000
ELAVL1	D3K5N7	0.88	1.00000000

H3C15	A0A286ZJQ0	0.74	1.00000000
CA2	F1RXC2	0.96	1.00000000
ARHGDIA	E7EI20	0.81	1.00000000
WNT9B	A0A287BJB4	5.36	1.00000000
XPO5	F1RRJ4	1.10	1.00000000
IDI1	A0A4X1T1T4	1.55	1.00000000
RPL13A	Q95307	1.18	1.00000000
CD68	F1ST28	-2.30	1.00000000
TNXB	K7GRK7	1.45	1.00000000
GMPPB	A0A8X9AEI1	0.88	1.00000000
ATP5MK	A0A4X1TQQ5	1.53	1.00000000
UBR4	F1SUQ5	1.02	1.00000000
SF3B4	F1SDG0	-7.76	1.00000000
NAGK	A0A480KE10	-1.84	1.00000000
CNDP2	F1SNL7	1.30	1.00000000
TMSB4	Q95274	0.95	1.00000000
	A0A287A4Q2	0.91	1.00000000
TBX2	F8SIP4	0.80	1.00000000
MTREX	F1SLL6	3.15	1.00000000
SKP1	A0A287ARU4	1.00	1.00000000
LPCAT1	F1S029	-4.50	1.00000000
C2H19orf53	F1SD77	0.82	1.00000000
SFPQ	A0A287A4F2	0.84	1.00000000
KRT19	A0A480J3V8	0.99	1.00000000
TUFM	F1RFI1	0.61	1.00000000
CRYM	F1RPC8	1.97	1.00000000
PZP	I3LQ17	-0.55	1.00000000
PSAP	P81405	-0.81	1.00000000
STK38L	I3LN61	4.25	1.00000000
ALDH1L2	F1SG41	-0.90	1.00000000
LOC100127131	A0A5G2RB77	1.81	1.00000000
FMO2	F1S6B6	0.71	1.00000000
ACTR6	A0A5G2QXQ2	-5.10	1.00000000
SEC61B	A0A4X1SH59	0.90	1.00000000
DERPC	A0A286ZRS9	1.00	1.00000000
	F1STC3	2.14	1.00000000
SPIN1	A0A286ZUJ2	1.03	1.00000000
RGCC	F1RJ89	0.90	1.00000000
PURA	A0A5G2RGK0	-0.73	1.00000000
H2AX	A0A5G2QMX0	0.99	1.00000000
DNAJB1	A0A4X1V5G4	5.55	1.00000000
BPGM	A0A286ZQ31	0.83	1.00000000
EIF1	F2Z553	-5.16	1.00000000
COL4A5	A0A287A007	0.57	1.00000000
DSP	F1RW75	1.23	1.00000000
NDUFS6	F1S031	1.83	1.00000000

ERGIC1	A0A8D0U9W6	1.07	1.00000000
NOP10	A0A4X1UUF9	0.92	1.00000000
RPL31	P62901	-2.94	1.00000000
EMC8	A0A5G2R2S6	3.38	1.00000000
CHD4	F1SLR5	1.89	1.00000000
YARS1	I3L804	1.02	1.00000000
TIMM8A	F1S1L1	1.27	1.00000000
FKBP1A	A0A5G2R9F7	1.23	1.00000000
NEBL	A0A8W4FA88	0.77	1.00000000
DHX15	F1S5A8	0.82	1.00000000
ECHS1	A0A287AA21	1.12	1.00000000
GLB1	I3LGI9	-1.59	1.00000000
RAB5A	Q06AU6	-0.96	1.00000000
AKAP6	I3L6S3	1.23	1.00000000
SLA-DQβ	P15982	4.99	1.00000000
AKAP12	A0A287A3A3	1.31	1.00000000
ITIH1	Q29052	-0.77	1.00000000
LOC100525572	A0A5G2QZG3	1.66	1.00000000
CD177	F1RMW7	0.64	1.00000000
MATN4	F1SDQ7	5.31	1.00000000
COX4I1	I3LER5	4.73	1.00000000
DCN	Q9XSD9	1.34	1.00000000
WDR13	I3LPL0	0.81	1.00000000
PRPS2	K7GL74	-1.31	1.00000000
RPL7A	A0A287BM53	1.42	1.00000000
ALDOA	A0A287B8F3	0.35	1.00000000
KHDRBS1	I3LP11	-0.68	1.00000000
CARHSP1	A0A4X1VPW4	0.98	1.00000000
FASN	I3LCW1	1.24	1.00000000
BASP1	A0A287ALA0	1.51	1.00000000
COL3A1	A0A286ZQ85	4.86	1.00000000
MARCKSL1	A0A287B4N8	2.12	1.00000000
RING1	A5D9P0	1.56	1.00000000
APOR	Q03472	-1.92	1.00000000
LRRC57	F1SI25	0.88	1.00000000
FLOT1	Q767L6	0.94	1.00000000
PLCB3	F1RQ08	1.23	1.00000000
HINT1	F1RKI3	1.78	1.00000000
BST2	A0A287A9J8	1.06	1.00000000
CAVIN2	I3LDR9	0.91	1.00000000
TMX1	A0A5G2QYQ1	5.12	1.00000000
HNRNPF	F1RG16	1.31	1.00000000
YAP1	A0A286ZNK4	1.18	1.00000000
GNG5	A0A287B6W0	1.29	1.00000000
USP9X	A0A287ACW6	1.23	1.00000000
SORBS3	F1RMA6	1.20	1.00000000

APEX1	A0A4X1UV69		1.04	1.00000000
RRAD	A0A286ZSB2		0.88	1.00000000
CMBL	A0A287A6V9		1.15	1.00000000
CLEC14A	F1SHL0		5.99	1.00000000
ALAD	A0A287BGW4		1.94	1.00000000
LY75	I3L5Y3		4.88	1.00000000
OLFML1	A0A286ZY90		1.13	1.00000000
FABP3	O02772		0.90	1.00000000
VAMP8	A0A8W4FA01		0.91	1.00000000
TEKT2	F1SV60		1.76	1.00000000
LUM	F1SQ09		1.42	1.00000000
	A0A8W4FGK7		1.56	1.00000000
CD59	O62680		0.79	1.00000000
WDR81	M3VHA7		0.55	1.00000000
DHRS7	F1SSI2		1.48	1.00000000
NME1	A0A287AS29		1.47	1.00000000
LAMB3	F1SF45		1.25	1.00000000
GFER	F1RFB9		5.15	1.00000000
LOC100621260	I3LMQ4		1.34	1.00000000
SNCG	A7YX24		1.53	1.00000000
AFG3L2	A0A8W4F7C8		2.30	1.00000000
SBSPON	A0A480WBM1		1.58	1.00000000
DCXR	A0A286ZQ44		1.26	1.00000000
CLIC1	A0A4X1VDN4		-0.44	1.00000000
NDUFV1	A0A4X1SZP7		-0.31	1.00000000
CRISP3	I3LVL5		1.52	1.00000000
WDR18	F1S6R5		6.28	1.00000000
	A0A287BAB5		1.45	1.00000000
LAMA4	F1RZM4		0.16	1.00000000
PECR	A0A4X1UM42		-0.97	1.00000000
NDUFA7	A0A8W4F7N8		0.97	1.00000000
CTSD	P00795		-1.94	1.00000000
LOC100520491	A0A286ZNW8		5.80	1.00000000
TPPP3	A0A4X1UMD5		1.37	1.00000000
GET3	A0A8D1SZ54		-1.61	1.00000000
OGN	A0A0H5ANC0		1.06	1.00000000
LYVE1	I3LTD0		1.45	1.00000000
ANP32A	A0A8D1TT21		0.69	1.00000000
PPP1R7	A0A8D0TG57		0.95	1.00000000
ARHGAP4	F1S291		-5.62	1.00000000
NDUFS4	A0A4X1SN37		-1.04	1.00000000
CRABP1	B3F0B7		1.40	1.00000000
LIMS1	A0A287BTD0		5.71	1.00000000
PARP1	I3LDH3		-0.49	1.00000000
SPR	A0A480ZCU6		0.71	1.00000000
RRAS2	A0A287A328		1.01	1.00000000

SULT1A3	I3LLX8	1.72	1.00000000
CYC1	A0A287AZF9	2.10	1.00000000
S100A9	K7GME6	1.67	1.00000000
MARCKS	A0A287BRL8	1.19	1.00000000
CD44	K7GM14	1.10	1.00000000
CAD	I3LLT2	-0.56	1.00000000
SCGB1A1	A0A4X1VM49	1.71	1.00000000
LAMA3	F1SBB3	4.31	1.00000000
COL6A3	A0A286ZVG7	1.09	1.00000000
COPS2	A0A5K1ULP5	0.83	1.00000000
KLKB1	A0A8D1M4K5	-1.35	1.00000000
LTA4H	F1SQR8	0.86	1.00000000
DTX3L	I3LRN0	7.76	1.00000000
HMCN1	A0A287BDY5	1.20	1.00000000
LBR	M3VH35	1.20	1.00000000
EIF5B	F1STE8	1.48	1.00000000

Table S5. Relative expression of antiviral genes in respiratory tissues following IAV infection. Mean value ± SEM. Mock groups are set to 1; IAV groups are scaled to their respective mock groups (lean-IAV to lean-mock and obese-IAV to obese-mock). P ≤ 0.05 is shown in bold.

A. Nasal mucosal tissue

Gene	lean-mock	lean-IAV	*P-value*	obese-mock	obese-IAV	*P-value*
CASP1	1.00 ± 0.32	2.35 ± 0.54	0.125	1.00 ± 0.10	1.51 ± 0.13	**0.038**
CD86	1.00 ± 0.18	1.38 ± 0.29	0.733	1.00 ± 0.19	1.20 ± 0.13	0.524
CTSS	1.00 ± 0.33	1.68 ± 0.36	0.354	1.00 ± 0.16	1.28 ± 0.12	0.271
CXCL10	1.00 ± 0.74	2.98 ± 1.19	0.149	1.00 ± 0.25	2.75 ± 0.50	**0.079**
CXCL8	1.00 ± 0.30	1.40 ± 0.38	0.668	1.00 ± 0.34	1.77 ± 0.74	0.504
CXCL9	1.00 ± 0.69	4.01 ± 1.80	0.147	1.00 ± 0.11	1.42 ± 0.19	0.142
GBP1	1.00 ± 0.34	2.09 ± 0.60	0.226	1.00 ± 0.08	3.35 ± 0.81	**0.001**
HSP70-2	1.00 ± 0.29	0.53 ± 0.08	0.174	1.00 ± 0.41	0.73 ± 0.15	0.685
HSPA6	1.00 ± 0.52	0.57 ± 0.16	0.803	1.00 ± 0.48	0.56 ± 0.19	0.429
IKBKE						
IRF7	1.00 ± 0.34	0.75 ± 0.10	0.784	1.00 ± 0.23	2.41 ± 0.37	**0.029**
IRF9						
ISG12A						
OAS2	1.00 ± 0.47	1.00 ± 0.18	0.642	1.00 ± 0.16	2.03 ± 0.37	**0.043**
SLA-1						
SLA-3						
STAT1	1.00 ± 0.23	1.40 ± 0.25	0.371	1.00 ± 0.10	1.70 ± 0.27	**0.042**
TAP1	1.00 ± 0.26	2.67 ± 0.47	**0.050**	1.00 ± 0.21	2.24 ± 0.38	**0.047**
TAP2	1.00 ± 0.25	2.22 ± 0.38	0.086	1.00 ± 0.19	2.07 ± 0.23	**0.042**
TLR7	1.00 ± 0.38	2.88 ± 0.89	0.162	1.00 ± 0.18	1.19 ± 0.16	0.534
TNFSF10	1.00 ± 0.37	1.59 ± 0.36	0.330	1.00 ± 0.09	1.49 ± 0.21	0.074

B. Upper tracheal tissue

Gene	lean-mock	lean-IAV	*P-value*	obese-mock	obese-IAV	*P-value*
CASP1	1.00 ± 0.42	1.86 ± 0.49	0.255	1.00 ± 0.14	2.82 ± 0.54	**0.008**
CD86	1.00 ± 0.41	1.87 ± 0.44	0.194	1.00 ± 0.15	2.34 ± 0.57	**0.040**
CTSS	1.00 ± 0.40	1.25 ± 0.25	0.468	1.00 ± 0.24	2.04 ± 0.34	0.058
CXCL10	1.00 ± 0.66	5.40 ± 0.54	0.082	1.00 ± 0.27	9.53 ± 2.79	**0.005**
CXCL8	1.00 ± 0.30	1.95 ± 0.53	0.327	1.00 ± 0.20	1.92 ± 0.43	0.338
CXCL9	1.00 ± 0.72	3.11 ± 0.56	0.208	1.00 ± 0.20	11.92 ± 6.82	**0.014**
GBP1	1.00 ± 0.50	7.85 ± 0.02	0.023	1.00 ± 0.10	7.34 ± 2.33	**0.009**
HSP70,2	1.00 ± 0.10	2.12 ± 0.60	0.053	1.00 ± 0.09	2.13 ± 0.40	**0.013**
HSPA6	1.00 ± 0.04	3.22 ± 0.81	0.282	1.00 ± 0.11	3.02 ± 0.80	**0.019**
IKBKE	1.00 ± 0.14	1.13 ± 0.14	0.651	1.00 ± 0.10	1.11 ± 0.15	0.752
IRF7	1.00 ± 0.33	2.70 ± 0.75	0.102	1.00 ± 0.23	5.64 ± 2.13	**0.008**
IRF9						
ISG12A	1.00 ± 0.51	1.91 ± 0.39	0.262	1.00 ± 0.18	4.58 ± 1.21	**0.002**
OAS2	1.00 ± 0.51	3.00 ± 0.81	0.111	1.00 ± 0.22	3.19 ± 1.03	0.069
SLA-1	1.00 ± 0.15	1.01 ± 0.30	0.402	1.00 ± 0.36	0.68 ± 0.14	0.979
SLA-3						
STAT1	1.00 ± 0.31	2.15 ± 0.40	0.099	1.00 ± 0.02	2.52 ± 0.55	**0.011**
TAP1	1.00 ± 0.14	2.30 ± 0.58	0.109	1.00 ± 0.27	3.18 ± 1.04	0.079
TAP2	1.00 ± 0.25	1.82 ± 0.38	0.167	1.00 ± 0.07	1.97 ± 0.45	0.065
TLR7	1.00 ± 0.27	1.25 ± 0.15	0.420	1.00 ± 0.08	2.51 ± 0.82	**0.050**
TNFSF10	1.00 ± 0.29	2.18 ± 0.41	0.117	1.00 ± 0.11	2.54 ± 0.57	**0.016**

C. Lung tissue

Gene	lean-mock	lean-IAV	*P-value*	obese-mock	obese-IAV	*P-value*
CASP1	1.00 ± 0.40	0.93 ± 0.25	0.886	1.00 ± 0.25	2.33 ± 0.59	0.120
CD86	1.00 ± 0.41	0.47 ± 0.08	0.288	1.00 ± 0.12	1.43 ± 0.25	0.294
CTSS	1.00 ± 0.52	0.47 ± 0.13	0.604	1.00 ± 0.27	3.53 ±1.24	0.151
CXCL10	1.00 ± 0.42	0.87 ± 0.16	0.877	1.00 ± 0.15	2.03 ± 0.60	0.352
CXCL8	1.00 ± 0.40	0.65 ± 0.12	0.752	1.00 ± 0.39	0.65 ± 0.11	0.804
CXCL9	1.00 ± 0.24	1.29 ± 0.33	0.823	1.00 ± 0.28	1.64 ± 0.74	0.838
GBP1	1.00 ± 0.16	0.65 ± 0.11	0.060	1.00 ± 0.37	1.03 ± 0.33	0.912
HSP70-2	1.00 ± 0.39	3.77 ± 1.72	0.339	1.00 ± 0.20	1.37 ± 0.30	0.645
HSPA6	1.00 ± 0.32	8.46 ± 4.14	0.086	1.00 ± 0.44	3.19 ± 1.36	0.232
IKBKE						
IRF7	1.00 ± 0.14	0.99 ± 0.38	0.214	1.00 ± 0.28	1.88 ± 0.35	0.257
IRF9						
ISG12A	1.00 ± 0.81	1.12 ± 0.44	0.359	1.00 ± 0.40	1.08 ± 0.37	0.641
OAS2	1.00 ± 0.48	0.40 ± 0.10	0.196	1.00 ± 0.26	1.68 ± 0.51	0.597
SLA-1						
SLA-3						
STAT1	1.00 ± 0.31	0.63 ± 0.06	0.401	1.00 ± 0.38	0.90 ± 0.22	0.821
TAP1	1.00 ± 0.39	1.46 ± 0.42	0.782	1.00 ± 0.18	0.55 ± 0.12	0.056
TAP2	1.00 ± 0.33	0.97 ± 0.17	0.927	1.00 ± 0.25	0.69 ± 0.14	0.338
TLR7	1.00 ± 0.11	1.15 ± 0.29	0.808	1.00 ± 0.48	0.69 ± 0.12	0.783
TNFSF10	1.00 ± 0.36	0.48 ± 0.09	0.210	1.00 ± 0.33	0.86 ± 0.18	0.785

Table S6. Diet compositions. The percentages are of total energy content.

Diet components	Standard diet (SD)	High fat, fructose, and cholesterol diet (5B4L) (HFFC)
Carbohydrates (%)	74.6	40.8
Protein (%)	18.6	16.2
Fat (%)	6.8	43
Fructose (%)	< 5.5	17.8
Cholesterol (ppm)	~0	20045
Choline (ppm)	784	668

Table S7: Reads mapping to reference genome (Sus_scrofa_NCBI_GCF_000003025.6_Sscrofa11.1).

Pig	Total clean reads (M)	Total clean bases (Gb)	Clean reads Q20 (%)	Total mapping (%)	Unique mapping (%)
104	89.07	8.91	98.11	98.79	95.32
108	88.93	8.89	97.92	98.2	94.95
111	89.2	8.92	96.09	98.25	94.11
113	88.92	8.89	98.01	98.74	94.98
124	88.93	8.89	98.03	98.51	94.62
125	88.79	8.88	97.78	98.33	94.82
127	89.16	8.92	96.07	98.29	93.47
130	89.01	8.9	98.06	98.65	94.74
135	88.86	8.89	98.26	98.54	95.26
148	92.8	9.28	96.63	98.31	94.51
149	89.2	8.92	96.03	98.17	94.56
156	88.88	8.89	97.95	98.35	95.29
157	89.06	8.91	98.09	98.65	94.97
432	88.85	8.88	98.1	98.8	95.45
435	89.09	8.91	98.09	98.87	94.32
448	88.91	8.89	98.05	98.47	93.93
454	89.06	8.91	98.29	98.79	95.41
457	88.79	8.88	98.04	98.69	95.39
460	89.07	8.91	98.21	98.77	95.08
461	89.16	8.92	98.07	98.56	94.56
465	89.06	8.91	96.84	98.57	94.77
466	89.05	8.9	97.16	98	92.3
474	89.08	8.91	98.03	98.8	95.72
486	89.03	8.9	98.04	98.43	94.26
487	89.03	8.9	97.04	98.83	94.04

Table S8. qPCR primer sequences for genes studied in lower trachea. Newly designed primer assays were designed in two duplicates indicated by (A) and (B) after the gene name.

Gene name	Forward primer	Reverse primer	Efficiency (%)
AMCF-II (A)	CAGAAGTGATAGCCACCCTGA	TTCCCACTGTCCAACATTTTC	107
AMCF-II (B)	CAGTGATCTGCAGGTGATCC	TTGGGTCCAGACAGACTTCC	112
B2M	TGAAGCACGTGACTCTCGAT	CTCTGTGATGCCGGTTAGTG	111
BST2 (A)	GTGGGTCTGCTGGTGTCTCT	TTGACATTCCTGCTGTGCTC	114
BST2 (B)	GGAGATGGAGAAGACGCAAA	CCTCCTGCAGCTTCTGATTC	111
CAPN14 (A)	GCAAACCACCTCTCTTCAGC	GGATACTCACGGTGCCAGAT	-
CAPN14 (B)	GGCCCTACTGGATCTTAATGC	AACATCAGCTGTTTCCACAGG	-
CASP1	GAAGGACAAACCCAAGGTGA	TGGGCTTTCTTAATGGCATC	119
CCL2	CTTCTGCACCCAGGTCCTT	CGCTGCATCGAGATCTTCTT	109
CCL20	CTGCAGCAAGTCAGAAGCAG	GCTGTGTGAAGCCCATGATA	107
CD36	GCCTATCCTCTGGCTTAATGAG	AACATCCCCACACCAACACT	109
CMPK2 (A)	CCAGAGAGGAGGTTGAGCTG	CATCCGCTGGTAGGACACTT	109
CMPK2 (B)	GGATGCCACAGGTAAAACCA	CAGGTGGCGACTTTAGGAGA	102
CXCL10 (A)	CCCACATGTTGAGATCATTGC	GCTTCTCTCTGTGTTCGAGGA	111
CXCL10 (B)	TTCGCTGTACCTGCATCAAG	CAACATGTGGGCAAGATTGA	111
CXCL11	CACCCAAGTAACAACTGTGACAA	TTGGGATTTAGGCATCTTCG	99
CXCL2	GAAGATGCTAAACAAGAGCAGTG	AGCCAAATGCATGAAACACA	114
CXCL8 (A)	TTGCCAGAGAAATCACAGGA	TGCATGGGACACTGGAAATA	113
CXCL8 (B)	GAAGAGAACTGAGAAGCAACAACA	TTGTGTTGGCATCTTTACTGAGA	117
DDX58 (RIG-I)	TTGCTCAGTGCAATCTGGTC	CTTCCTCTGCCTCTGGTTTG	104
DDX60	TCTGGACCATGTCACTCTGC	TGGCATTTTTCTTCCTTGGT	109
DHX58	GAGTACCAGGCCAAGATCCA	CTTCTGCTGCCGACTCTTCT	-
ETV7	TAAGAAGGAACCTGGGCAGA	CCTCGTCCTGGATGGTCTT	106
FTO	TTGGCGATACCCCTTCAC	CTCGGTGGGTGGAACTAAAC	103
GAPDH	ACCCAGAAGACTGTGGATGG	AAGCAGGGATGATGTTCTGG	113
HERC5	AGCCATCGTTGGAAGACTTG	TTCCAAAGTCGTCGTCTTCA	107
HSPA6 (A)	CCAGTTGGCAGAGAAAGAGG	GGAGAAGATGGGACGACAAA	92
HSPA6 (B)	CACAACCAGTTGGCAGAGAA	GGAGAAGATGGGACGACAAA	108
IFI6	AAGGCGGTATCGCTCTTCTT	GAGCTGCTGTTGTCCTCAGA	108
IFIH1 (MDA5) (A)	CAGTGTGCTAGCCTGCTCTG	GCAGTGCCTTGTTTCCTCTC	108
IFIH1 (MDA5) (B)	TCGGATTTTGGAACTCAACC	TCTTTGCGATTTCCGTCTCT	113
IFIT1 (A)	ACTGCAGCCCAACAGTCTTT	TGATTTGCAGCTCCATTCTG	109
IFIT1 (B)	GGCCATTTTGTCTGAATGCT	TCAGGGCAAAGAGAGCCTTA	111
IFIT2 (A)	GGACACGCTCTCCATCATTT	ACTGACCAGCTTGCACACAG	103
IFIT2 (B)	CAATCATGAGTGAGACCACAAAG	CCAAGTTCCAGGTGAAATGG	105
IFIT3 (A)	ACTGCAGCCCAACAGTCTTT	TGATTTGCAGCTCCATTCTG	109
IFIT3 (B)	GAACAGCCCTTCAGGCATAG	TCCATTTCCTCAGTGCCTTC	114
IFITM1	CACCACGGTGATCACCATCC	GCACCAGTTCAGGAAGAGGG	119
IFITM3	ACCACGGTGATCAACATCCG	AGCACCAGTTCATGAAGAGGG	116
IFNB (A)	AGCACTGGCTGGAATGAAAC	TCCAGGATTGTCTCCAGGTC	-
IFNB (B)	AACCGTCATTAAGACTATCCTTGTG	CTCCTCCATGATTTCCTCCA	109
IFNL1	ATGGGCCAGTTCCAATCTC	CTGCAGCTCCAGTTCTTCAGT	109
IL10	TACAACAGGGGCTTGCTCTT	GCCAGGAAGATCAGGCAATA	109

IL17C (A)	GGATCCAGTGCCAGGTTCT	TGTCCACACGGTATCTCCAG	-
IL17C (B)	GCTGCATCAGCACCAAGAC	CAGCAGGCTCTGGGTCAG	-
IL18	CTGCTGAACCGGAAGACAAT	TCCGATTCCAGGTCTTCATC	107
IL18	CAATTGCATCAGCTTTGTGG	TCCAGGTCCTCATCGTTTTC	101
IL1B	CCAAAGAGGGACATGGAGAA	GGGCTTTTGTTCTGCTTGAG	109
IL1RN	TGCCTGTCCTGTGTCAAGTC	TGCCTGTCCTGTGTCAAGTC	99
IL6	CCTCTCCGGACAAAACTGAA	TCTGCCAGTACCTCCTTGCT	106
IRF7 (A)	GTGTGCTCCTGTACGGGTCT	CTGCAGCAGCTTCTCTGTGT	109
IRF7 (B)	GCTCCCCACACTACACCATC	TCCAACTTCACCAGGACGA	96
ISG15	AGTTCTGGCTGACTTTCGAGG	GGTGCACATAGGCTTGAGGT	119
ISG20 (A)	CGAGGCTGACCTGCACTACT	CCAACACACTGTCCTGGATG	119
ISG20 (B)	AGATCCTGCAGCTCCTGAAA	TGCTCATGTTCTCCTTCAGC	114
LEPR (A)	CTGCTCTGGAAGCCTCTGAT	TGGCGGGAAGTATGATGTTT	103
LEPR (B)	GCTCAGAATCCCTTCCTCTGT	CCCCACTCCTTCCATGAATA	119
LGALS9 (A)	ATCCCATGATCCCACCTATG	GATGCTCTTGGAGGGGTACA	112
LGALS9 (B)	CAGAGCTTCTTGGTGTGGAT	AAATAGGTGCTGACCGTCCA	110
MUC5B	GGACAACTGCACGGAGTACC	CCTTTTTCCGGAGGATGC	115
MX1 (A)	GCCGAGATCTTTCAGCACCT	CGGAGGATGAAGAACTGGATGA	117
MX1 (B)	CCATCATTAAGAAGCAGGTCAGTG	AACATCTGTGAAGGCGAGCC	111
MX2	ACCAAGGGCCTGAATATGCT	ACGGGCTGTACAGGTTGTTC	118
NFIL3 (A)	GTCAACCGGTCCTCCAGTAA	TCCCCATCAGATGACTTTCC	111
NFIL3 (B)	TGGTTAAAGTCCCGGAAGTG	TCGAAGGCTTCCACTTTGAT	108
OAS1	AAGAAACCCAGGCCTGTGATTC	TAGTGCCCCTTCTACCAGCT	117
OAS2	GATAATTCGTGCTGGCACCT	GATGGTCCAGGTGACTCGTT	108
OASL	TGCGACTGGTAAAACACTGG	CCCAGGCATAGATGGTCAGT	117
POMC (A)	CCGAGAAGAAGGACGAAGG	CTTCTCGGAGGTCATGAAGC	114
POMC (B)	TAACTTGCTGGCGTGCATC	GTTCTCGGTCAGCGGTTG	-
PPARGC1A	TGCTGTGGGGATGTCTCATA	CTGCCAACAGCTTCTCCTTC	100
PPIA	CAAGACTGAGTGGTTGGATGG	TGTCCACAGTCAGCAATGGT	113
RELA	CGCAAAAGGACCTATGAGACC	GATAGGGCTGGGGAGCTG	-
RPL13A	ATTGTGGCCAAGCAGGTACT	AATTGCCAGAAATGTTGATGC	117
RSAD2	AAAGACGTGTCCTGCTTGGT	GCCCGTTTCTACAGTTCAGG	104
SOCS3	GCTGGTGCATCACTACATGC	CGACGAGGAGGGTTCAGTC	109
TMPRSS11D	TTGAGTGGAAGAATTGAATCTATGA	GACAACATGAGCTCTCACGAA	103
TMPRSS2	ATGGGCTACCGGAATAGCTT	TGTTGGCACTTTTGTTCAGC	114
TRIM25	ACAAGGAATCACCACGAAGC	CAGACGCCCTTTATCAGCTC	104
USP18	CAATGACTCCAATGTCTGTTGG	AGCGGAAGCTGTGATTTCC	109
XAF1 (A)	ATTCCAGCTCCTGAAAGCAA	GGACAGCATCTACCCAGGTG	107
XAF1 (B)	CACCAAGAGGCACAAACAAA	GGATGTCATAGGCTGCATCA	115
YWHAZ	GCTGCTGGTGATGATAAGAAGG	AGTTAAGGGCCAGACCCAAT	115
ZBP1	CCCAGAGGATGCAGAGCTT	GGTTATCAAATGCCAGGTGC	109

Table S9. qPCR primer sequences for genes studied in nasopharyngeal swabs. Newly designed primer assays were designed in two duplicates indicated by (A) and (B) after the gene name.

Gene name	Forward primer	Reverse primer	Efficiency (%)
ACTB	CTACGTCGCCCTGGACTTC	GCAGCTCGTAGCTCTTCTCC	108
ASNS (A)	CCGGATCCTTTTTGACAATG	TGATAAAAGGCAGCCAATCC	101
ASNS (B)	GGTCCAGATGCATTTCGTTT	CATTCCAAACAGCTGGTCAA	97
BST2 (A)	GTGGGTCTGCTGGTGTCTCT	TTGACATTCCTGCTGTGCTC	104
BST2 (B)	GGAGATGGAGAAGACGCAAA	CCTCCTGCAGCTTCTGATTC	100
C1QBP (A)	GGGGACAAAGCTTTTGTTGA	CCAGACATCTTGGGGAGAGA	98
C1QBP (B)	GCTGGAAGTGAATGGGACAG	GTGGGATGCTGTTGTTGATG	107
CASP1	GAAGGACAAACCCAAGGTGA	TGGGCTTTCTTAATGGCATC	114
CCL2	CTTCTGCACCCAGGTCCTT	CGCTGCATCGAGATCTTCTT	103
CCL8	GCCAGATTCAGTCTCCATCC	AGGGGATCTTTCCATTGACC	106
CD163	CACATGTGCCAACAAAATAAGAC	CACCACCTGAGCATCTTCAA	
CD200R1 (A)	CGTGGCTATCACCTCCAAGT	TTGCACACCACAGTTCCATT	
CD200R1 (B)	AATGGAACTGTGGTGTGCAA	AGTGACACAGTCCCCCTCAG	110
CD55 (A)	TGTCCGACTCCTGGAGAACT	AGATGGATGCGCCAAATAAC	97
CD55 (B)	AATGTCCGACTCCTGGAGAA	GGATGCGCCAAATAACAAGT	97
CH25H (A)	GTACCGGACCTTCCACAAGA	AAGTAGCTCCCCGACACTCA	109
CH25H (B)	TATGACTTCCCCTGGTCCAC	GTAGGTCGTGGTGTGTCACG	115
CXCL10 (A)	CCCACATGTTGAGATCATTGC	GCTTCTCTCTGTGTTCGAGGA	101
CXCL10 (B)	TTCGCTGTACCTGCATCAAG	CAACATGTGGGCAAGATTGA	92
CXCL11	CACCCAAGTAACAACTGTGACAA	TTGGGATTTAGGCATCTTCG	107
CXCL9	TTAAACAATTTGCCCCAAGC	TGTTTGATCCCCATTCTTCA	102
DDX58/RIGI	TTGCTCAGTGCAATCTGGTC	CTTCCTCTGCCTCTGGTTTG	92
EIF2AK2	AGAACTTCTTCACATATGTCGCAC	ATACATCTAAGACGCCAGCCTT	100
EIF4G1 (A)	GAAGAAATCCAAGGCCATCA	GAAGAGCAGTGAGGGTGAGG	103
EIF4G1 (B)	TCATCAGCTGCTGGAACATC	GTGGACCCCACTGTGAAAAT	100
GAPDH	ACCCAGAAGACTGTGGATGG	AAGCAGGGATGATGTTCTGG	92
HPRT1	ACACTGGCAAAACAATGCAA	TGCAACCTTGACCATCTTTG	88
IFI44	ATTGCTCACTCACGTGGACA	GCTTGAGTTTCACAGGCACA	97
IFIT1	ACTGCAGCCCAACAGTCTTT	TGATTTGCAGCTCCATTCTG	92
IFIT3	GAACAGCCCTTCAGGCATAG	TCCATTTCCTCAGTGCCTTC	101
IFITM1	GCTTTCGCCTACTCCGTGA	CCAGGATCAGAGCCCAGATG	
IFITM3	ACCACGGTGATCAACATCCG	AGCACCAGTTCATGAAGAGGG	
IFNA	CATGAGATGCTCCAGCAGAC	AGCTGCTGATCCAGTCCAGT	
IFNAR	TTGTGTCAAAGCCAGAGCAC	TTCCTGGTTTGGTTTTCTCG	103
IFNB	AACCGTCATTAAGACTATCCTTGTG	CTCCTCCATGATTTCCTCCA	101
IFNG	AAGAATTGGAAAGAGGAGAGTGAC	TCACATCCATGCTCCTTTGA	103
IFNL3	CCTGGAAGCCTCTGTCATGT	TCTCCACTGGCGACACATT	99
IL12A	CCACCTGGACCATCTCAGTT	CAGCAGATTTTGGGAGTGGT	95
IL15	CGTCATTTTGCAAGAGTCCA	TGGACGATAAACTGCTGTTTGC	92
IL18	CAATTGCATCAGCTTTGTGG	TCCAGGTCCTCATCGTTTTC	91
IL1A	TGTGCTAAATAACCTGGATGAGG	GGTTCGTCTTCGTTTTGAGC	
IL1B	TCTCTCACCCCTTCTCCTCA	GACCCTAGTGTGCCATGGTT	102

IL1RN	TGCCTGTCCTGTGTCAAGTC	GTCCTGCTCGCTGTTCTTTC	85
IL2	CCAAGCAGGCTACAGAATTG	TTTGCTGAGTCAGAGTTTTTGC	
IL6	CCTCTCCGGACAAAACTGAA	TCTGCCAGTACCTCCTTGCT	105
IL8	GAAGAGAACTGAGAAGCAACAACA	TTGTGTTGGCATCTTTACTGAGA	
IRF1	TGAAGCTGCAACAGATGAGG	CTTCCCATCCACGTTTGTCT	109
IRF2	GATGCTGCCCTTATCTGAGC	TGTGCTTCACTCTGTCTTCCTT	107
IRF3	GCTACACCCTCTGGTTCTGC	GAGACACATGGGGACAACCT	111
IRF7	GCTCCCCACACTACACCATC	TCCAACTTCACCAGGACGA	111
IRF9	ATCAAGTGTCCCTGGAGCAT	CAGTGTCACCTGGAACTTGG	114
ISG15	AGTTCTGGCTGACTTTCGAGG	GGTGCACATAGGCTTGAGGT	105
ISG20	AGATCCTGCAGCTCCTGAAA	TGCTCATGTTCTCCTTCAGC	116
LY96	CAGTAAAGGTTGAGCCCTGTG	TTTGCGCATTGGTAAAGTCA	
MAVS	ATCTCGCTGACGAAGTGTCC	GCCTCAGCAGGAGTAGATGG	108
MDA5 / IFIH1	CAGTGTGCTAGCCTGCTCTG	GCAGTGCCTTGTTTCCTCTC	95
MX1	CCTCCACAGAACTGCCAAG	GCAGTACACGATCTGCTCCA	117
MYD88	CCAGACTAAGTTTGCACTCAGC	AGGATGCTGGGGAACTCTTT	98
NFKB	CCCTGTGAAGACCACCTCTC	ATCCCGGAGCTCGTCTATTT	118
NLRP3	GACTTTCCAGGAGTTCTTTGCTG	CCTGGTTTACAAGGCCAAAG	
NOD2	GAAAGTCCTGAAGCTGTCCAAC	CCAGACTTCCAGGATGGTGT	
OAS	AAGAAACCCAGGCCTGTGATTC	TAGTGCCCCTTCTACCAGCT	115
OAS2 (A)	GATAATTCGTGCTGGCACCT	GATGGTCCAGGTGACTCGTT	104
OAS2 (B)	TTTAATTCGCCTGGTGAAGC	GCAGCTCCAAGGCATACTTC	114
OASL (A)	TGGTACCTGAAGTACGTGAAAGC	TACCCACTTCCCAGGCATAG	
OASL (B)	TGCGACTGGTAAAACACTGG	CCCAGGCATAGATGGTCAGT	103
PABP1 (A)	GCCTTTGACTGCTTCCATGT	GTAGGGTGCATGGCTTGAAT	107
PABP1 (B)	CAAGCCAGTACGCATCATGT	TGAATATGTTGCCCACTCCA	110
PAF1 (A)	GGGAACCAGTTTGTGGCTTA	ACACATCATCTGGCGCATAA	92
PAF1 (A)	TTTGAGGATGCCCAGAAATC	AAGACAGGCATGACCTCCAC	96
PPIA	CAAGACTGAGTGGTTGGATGG	TGTCCACAGTCAGCAATGGT	
RNASEL	GGAGAGCCGTTACAGGACC	TCAGATGTTCCAGGTTGCAGT	95
RSAD2	AAAGACGTGTCCTGCTTGGT	GCCCGTTTCTACAGTTCAGG	97
SFTPA1	ACCTTCTTGTTGCTGGCAGT	CAGGGTCTCCTTTGATTCCA	
SFTPB (A)	GAGAATGGCTACCGCAAGAC	CACTGCTGTTCTCTGCCTGA	
SFTPB (B)	CACTCAGGCAGAGAACAGCA	CGAACTGCTCACACTTTTGC	
STAT1	CACGAAGGTGATGAACATGG	TCTTTCAGTTGCAGGTGTCG	104
STAT2	TTTGCCCCATGATCTGAGACAC	ACGTTGGTGTTCTGGCTAGC	86
TLR3	ATTGTGCAAAAGATTCAAGGTG	TCTTCGCAAACAGAGTGCAT	
TLR7	GGAAATAGCATCAGCCAAGCTC	TTCCAGGTTGCGTAGCTCTT	117
TMPRSS2 (A)	ATGGGCTACCGGAATAGCTT	TGTTGGCACTTTTGTTCAGC	97
TMPRSS2 (B)	GTGATTTCCCACCCGAACTA	GTTGAAGGTCATTGGCGTCT	92
TRIM22 (A)	CTGGAAGGGAAGAGCAACTG	TGTTGGCCACATGCCTATTA	104
TRIM22 (B)	ATGCTGAAGGACGAGGAGAA	GAGATGAGCTCTCCCACCAG	
TRIM25 (A)	ACAAGGAATCACCACGAAGC	CAGACGCCCTTTATCAGCTC	115
TRIM25 (B)	GGTGGAACTTGCCCTGACTA	GCTTCGTGGTGATTCCTTGT	109

Table S10. qPCR primer sequences for genes studied in nasal mucosa, upper trachea, and lung.

			Efficiency (%)		
Gene name	Forward primer	Reverse primer	Nasal mucosa	Upper trachea	Lung
CASP1	GAAGGACAAACCCAAGGTGA	TGGGCTTTCTTAATGGCATC	0.99	1.07	1.10
CD86	CATCGTCTGTGTCCTGCAAC	CACAGGTGGCTTTGCATCTA	1.14	1.09	1.06
CTSS	GTGAAAAACAGCTGGGGACT	TGGGTAAGAGGGATAATTAGCAA	0.96	1.06	1.12
CXCL10	TTCGCTGTACCTGCATCAAG	CAACATGTGGGCAAGATTGA	1.10	1.05	1.14
CXCL8	GTGGACCCCACTGTGAAAAT	CAACCTTCTGCACCCACTTT	0.97	1.02	1.11
CXCL9	AGCAGTGTTGCCTTGCTTTT	ATGCAGGAACAACGTCCATT	1.12	1.07	1.07
GBP1	TATCTTGAGACGCCCAGGAA	CCCACAGACTCCTTGGACTT	1.05	1.07	1.17
HSP70-2	CAAGATCACCATCACCAACG	TCGTCCTCCGCTTTGTACTT	1.12	1.14	1.17
HSPA6	CCAGTTGGCAGAGAAAGAGG	GGAGAAGATGGGACGACAAA	1.10	1.07	1.04
IKBKE	CATGAGACCAGGAACCACCT	AAAGCCTCAGCCAGATCCTT		1.03	1.12
IRF7	GCTCCCCACACTACACCATC	TCCAACTTCACCAGGACGA	1.18	1.09	1.11
IRF9	CATTCAGACTTGGGGAGCAG	AAAGGGGCCTCAGTGGTAAC			
ISG12A	GCCTGGCTTCCAAGATACTG	AGCCCTGCTGAAGGAAGTC	1.17	1.11	1.19
OAS2	GATAATTCGTGCTGGCACCT	GATGGTCCAGGTGACTCGTT	1.07	1.09	1.10
PPIA	CAAGACTGAGTGGTTGGATGG	TGTCCACAGTCAGCAATGGT	1.08	1.10	1.16
RPL13A	ATTGTGGCCAAGCAGGTACT	AATTGCCAGAAATGTTGATGC	1.16	1.13	1.13
SLA-1	GGGTCCCCACTCCCTAAG	ACGTAGCCGACTTCGATGA		1.15	
SLA-3	CCCTCGTTCTGCTGCTCT	CGGCGGTGTAGAAATAGCTC			
STAT1	CACGAAGGTGATGAACATGG	TCTTTCAGTTGCAGGTGTCG	1.10	1.03	1.09
TAP1	CCCCTTCCTGAATATGAGCA	CAGGCCATAGGCAATGTTTT	0.92	1.08	1.18
TAP2	GAGCATCACTACCTGCACCA	AGCTCTTCAGCCCATAAGCA	1.02	1.04	1.19
TLR7	GGAAATAGCATCAGCCAAGCTC	TTCCAGGTTGCGTAGCTCTT	1.08	1.10	1.08
TNFSF10	TACAGTTTCAGAAAAGCAACAAGG	GTGAGCAGCCACTCTCTGTG	1.05	1.04	1.06
YWHAZ	GCTGCTGGTGATGATAAGAAGG	AGTTAAGGGCCAGACCCAAT	1.15	1.09	1.10
